# Investigating the energy crisis in Alzheimer disease using transcriptome study

**DOI:** 10.1038/s41598-019-54782-y

**Published:** 2019-12-06

**Authors:** S. Akila Parvathy Dharshini, Y.-h. Taguchi, M. Michael Gromiha

**Affiliations:** 1 Department of Biotechnology, Bhupat and Jyoti Mehta School of Biosciences, Indian Institute of Technology Madras, Chennai, 600036 Tamilnadu India; 2 Department of Physics, Chuo University, Kasuga, Bunkyo-ku, Tokyo 112-8551 Japan

**Keywords:** Computational biology and bioinformatics, Genome informatics

## Abstract

Alzheimer disease (AD) is a devastating neurological disorder, which initiates from hippocampus and proliferates to cortical regions. The neurons of hippocampus require higher energy to preserve the firing pattern. In AD, aberrant energy metabolism is the critical factor for neurodegeneration. However, the reason for the energy crisis in hippocampus neurons is still unresolved. Transcriptome analysis enables us in understanding the underlying mechanism of energy crisis. In this study, we identified variants/differential gene/transcript expression profiles from hippocampus RNA-seq data. We predicted the effect of variants in transcription factor (TF) binding using *in silico* tools. Further, a hippocampus-specific co-expression and functional interaction network were designed to decipher the relationships between TF and differentially expressed genes (DG). Identified variants predominantly influence TF binding, which subsequently regulates the DG. From the results, we hypothesize that the loss of vascular integrity is the fundamental attribute for the energy crisis, which leads to neurodegeneration.

## Introduction

Alzheimer disease (AD) is one of the most common neurodegenerative disorders, which impacts more than 47 million people globally with a steady increasing mortality rate^1^. This disease is initiated in the hippocampus neurons located in the medial temporal lobe and spreads to the cortical regions of the brain^2^. The selective vulnerability of the specific neuronal subset is a common factor for most neurodegenerative disorders and this selectively vulnerable neuron requires high energy to preserve the functional property, such as firing rate, ion homeostasis and synaptic transmission^3^. Therefore, disruption in energy metabolism leads to energy demand that eventually results in functional abnormality and cellular stress.

Metabolic stress is reported to be the dominant factor for selective vulnerability with a reduction in glycolytic and oxidative phosphorylation enzymes in AD^4,5^. Based on genomic and transcriptomic studies, Wang *et al*.^3,6^ showed that hippocampus neurons are significantly susceptible to oxidative stress due to upregulation of reactive oxygen species (ROS) and downregulation of antioxidant enzymes. This metabolic stress and ROS affects the mitochondrial DNA that further impairs mitochondrial morphology and stimulates the formation of inner mitochondrial transition pore (MTP), which leads to apoptosis and aberration in the electron transport chain enzymes, resulting in the defective metabolic process^7,8^. Aberration in calcium dynamics disrupts the mitochondrial morphology that induces energy demand^9^. The gene expression studies illustrate that upregulation of N-methyl-D-aspartate receptors (NMDA) promotes excitotoxicity and disrupts calcium dynamics and energy metabolism in hippocampus neuron^10^.

Microarray data and large-scale co-expression network studies demonstrate an imbalance in the energy metabolism associated with neurodegeneration^11–13^. Transcriptome-wide association studies of AD revealed that noncoding variants associated with AD-susceptible genes disrupt splicing and gene expression patterns which cause tau protein aggregation^14^. Even though the availability of experimental and computational studies mainly refer the defects in energy metabolism and protein degradation which leads to neurodegeneration^15–17^, the reason for the energy crisis in neurodegeneration has not yet been explored. It is also essential to identify the underlying mechanism of the disease to determine a therapeutic strategy. Analyzing high-throughput hippocampus RNA-seq data may shed light to understand the disease mechanisms and provide profound insights into the cellular pathway, which helps in recognizing a potential drug target.

RNA-seq data plays an important role in identifying differential expressions, variants and fusion gene detection. In this study, we retrieved hippocampus RNA-seq data from sequence retrieval archive (SRA) database^18^. We identified variants from RNA-seq data using genome analysis tool kit (GATK) and compared the predicted variants with expression Quantitative trait loci (eQTL), Genome-wide association study (GWAS) for various neurological disorders^19–21^. GWAS study reports the abundance of variants in the disease population, but it does not show the effect of variants. Using *in silico* tools, we predicted the effect of variants on the transcription factor (TF) binding and epitranscriptomic modifications. Thus, we have identified 297 reported GWAS variants and 20 novel variants, where most of the identified variants disrupt TF binding.

We performed differential expression analysis in three different levels: (i) gene expression (ii) transcript expression (iii) transcript proportions. We identified 250 genes, which are differentially expressed at gene and transcript levels. To understand the relationship between predicted variants associate genes (VG), differentially expressed genes (DG) and TF, we built hippocampus-specific co-expression and functional module networks. From large-scale network studies, we found that most of the disrupted TFs are impacting the gene expression of differentially expressed genes.

Further, enrichment studies illustrate the dysregulation of novel DG is responsible for the blood vessel morphogenesis, blood circulation, gliogenesis, mitochondria biogenesis, ROS response, lipid metabolism, endothelial and smooth muscle cells dysfunction. This study depicts the loss of vascular structural and functional integrity, which is the primary factor for the energy crisis in hippocampus neuron. We propose that restoring vascular dynamics and the blood-brain barrier (BBB) may save the surviving neuronal population against energy crisis.

## Results

### Variant calling-hippocampus RNA-seq data

We have obtained the variants from stage 6 of Alzheimer patients using GATK tools by employing STAR spliced aligner and hg38 genomic assembly. We identified 297 significant GWAS and 20 novel variants (Supplementary Table S3, Fig. S2). These 20 variants are unique and not reported in any of the neurological disorders (termed as “novel”). Among them, 136 GWAS variant associated genes (VG) and 6 novel VG are differentially expressed (DG) in AD (Supplementary Table S3).

### Effect of variants in methylation, histone acetylation and transcription factor binding

The variants located in conserved transcription binding sites or cis-regulatory elements can potentially interrupt TF binding. In addition, the epigenetic modifications such as methylation and histone acetylation play an essential role in controlling the gene expression profile^22^. We evaluated the impact of variants in TF binding and epitranscriptomic modifications and the number of variants altering gene expression, methylation, histone acetylation and TF binding are presented in Supplementary Table S4.

Most of the variants (186) interrupt TF binding and 25 variants affect methylation and TF binding.The variants identified in our studies are predominantly located in non-coding regulatory regions, and the disrupted TFs has been matched with the AD expression profiles from the literature study^23^. Supplementary Table S3 represents the effect of GWAS and novel variants. We analyzed the impact of epigenetic modification on non-coding variants and the results are presented in Table 1. TIMM44 gene regulates the translocation of anti-oxidant enzymes and reduces the ROS response. Variants associated with this gene disrupt methylation, gene expression and as well, interrupt the binding of 8 TFs (NANOG, GLI1, TAL1, CCNT2, TCF12, YY1, XZF, ESR1). The upregulated TFs NANOG and GLI1, involved in hedgehog signaling are essential for regeneration during tissue injury^24^. The downregulated TFs such as TCF12, TAL1, YY1 and ZFX participate in neural and oligodendrocyte differentiation. ESR1 helps to maintain hippocampal memory function by regulating apolipoprotein.

**Table 1:** Effect of predicted variants on epigenetic modifications and TF binding.

Category	Variant associated gene name	Differential gene expression study in AD	Chr	Position	Ref/Alt	Genomic location	SNP ID	Dysregulated regulatory elements in AD	GWAS/eQTL study
Methylation, Gene expression and TF binding	TIMM44	DOWN	19	7935842	A/G	intronic	rs35065193	Nanog (UP), TCF12, TAL1(DOWN)	AD
				7927167	A/C	UTR3	rs12976850	ESR1, YY1, ZFX, CCNT2 (DOWN)	AD
				7930402	G/A	intronic	rs34629355	GLI1 (UP)	AD
	OGFOD3	DOWN	17	82390132	A/C	UTR3	rs11650671	JUN (DOWN)	ALS
	RPS20	DOWN	8	56069265	C/A	intronic	rs2953901	JUN (DOWN)	AD
	PSMC3IP	DOWN	17	42573638	G/C	intronic	rs2292752	SIX5 (DOWN)	AD, AMD
	ACP1	DOWN	2	266895	C/T	intronic	rs10171043	IRF1 (DOWN), MEF2A (UP)	AD
	FAM13A	UP	4	88754499	G/A	intronic	rs61231054	ZBTB7A (UP)	AD
	RPS20	DOWN	8	56073972	C/G	intronic	rs2976045	NF1 (DOWN)	AD
	MAN1B1	DOWN	9	137103590	C/T	intronic	rs28373932	RREB1 (DOWN), ZBTB1 (UP)	**Novel**
Histone acetylation, Gene expression and TF binding	RBFOX1	UP	16	7222542	G/A	intronic	rs12935687	RFX5 (DOWN), DNMT1(UP)	AD
	CDC27	DOWN	17	47055097	G/A	intergenic	rs11079748	Nanog (UP), TCF12, MLLT1 (DOWN)	AD
	HBS1L	UP	6	135048076	G/A	intronic	rs6915770	GFI1b (DOWN), HEY1(UP)	AD

PSMC3IP gene modulates the proteasomal activity and the variant, G/C (rs2292752) associated with this gene interrupts TF SIX5, which is essential for retinal function and is involved in AD and AMD.

We identified a novel variant, C/T (rs28373932) in gene MAN1B1, which is involved in quality control and degradation process and it is downregulated in AD. MAN1B1 also interrupts the TF involved in axon degradation signaling cascade (RREB1) and regulation of immune response (ZBTB1)^25^.

**Figure 1:**
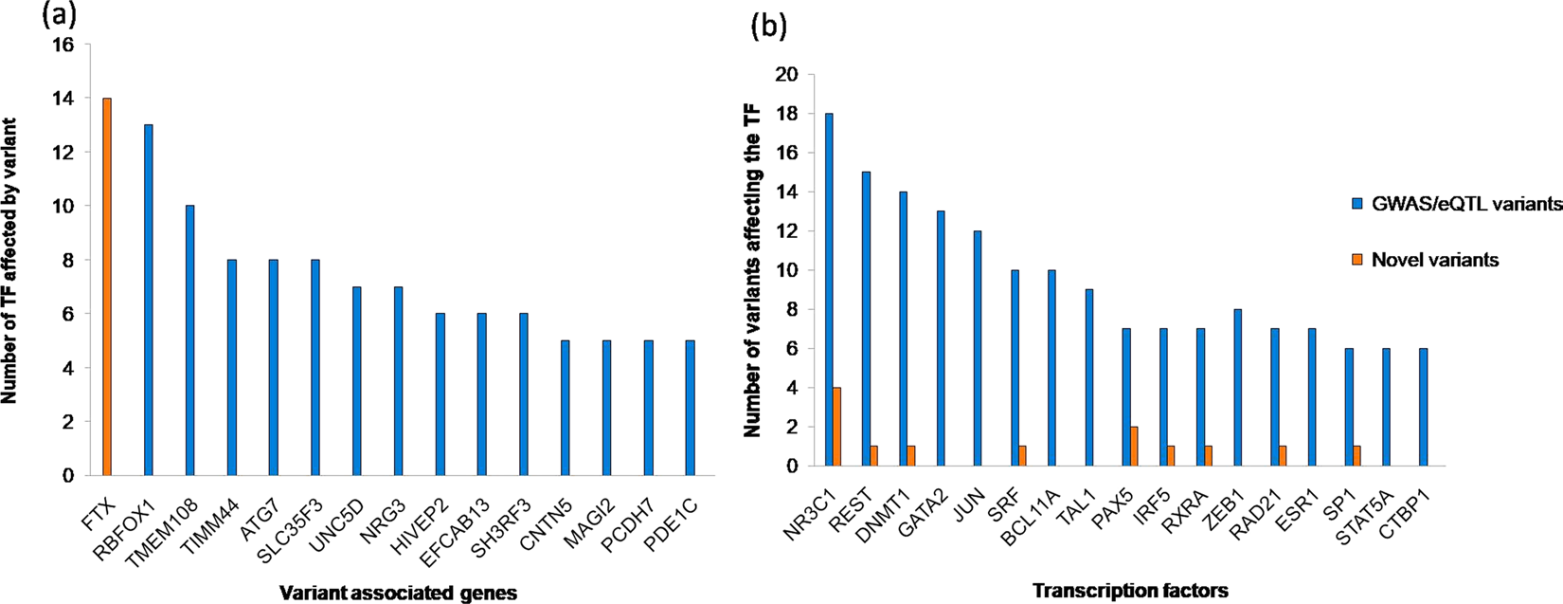
(**a**) The number of TFs affected by VG (**b**) The number of variants disrupting TFs.

We have analyzed the number of TFs interrupted by variants and the number of variants influencing TF binding, and the data are presented in Fig. 1. Figure 1a shows that the FTX gene (associated novel variants) disrupts the binding of 14 different TFs (Supplementary Table S3). FTX belongs to the class of noncoding RNA and it controls DNA methylation and miRNA regulation. Variants in this gene may impact heterochromatin organization^26^. RBFOX1 variants reported in AD disrupt the binding of 13 TFs (Supplementary Table S3**)** and a few of them influence methylation and histone acetylation. Further, this gene is upregulated in AD and involved in synaptic migration. The knockdown of this gene resulted in the aberrant structural integrity of the hippocampus neuron^27,28^. TMEM108 variants reported in AD, ALS and AMD also influence 10 TFs. This gene is involved in cognitive function and regulates the axonal transport.

Figure 1b represents the top 10 TFs disrupted by large number of variants, such as NR3C1, REST and DNMT1. NR3C1 plays a significant role in stress response and is upregulated in AD^29^. REST is essential for neuroprotection and neurogenesis and is downregulated in AD^30^. DNMT1 is involved in epigenetic regulation that helps preserve methylation patterns and upregulated in AD. Our gene expression studies also showed that NR3C1 and DNMT1 are 3-fold upregulated (Supplementary Fig. S3**)**.The variants present in FTX and RBFOX1 disrupt the TFs binding of NR3C1, REST and DNMT1.

We have explored the variants affecting the TFs which are not reported in AD gene expression profile (termed as “novel TFs”) such as CUX1, DIDO1 and RORA, which are found to be differentially expressed from our study (Supplementary Fig. S3**)**. CUX1 acts as a repressor of dendrite morphology regulation^31^ and inhibition of CUX1 promotes dendritic arborization. DIDO1 gene participates in apoptosis. RORA is involved in blood vessel morphogenesis, glucose metabolism, immune response, glutamate signaling, cholesterol metabolism and neuronal survival^32^. In AD, we observed 6-fold upregulation of CUX1, DIDO1 and 3-fold upregulation of RORA.

### Differential gene/transcript expression analysis of hippocampus RNA-seq

We computed DG obtained from transcriptome-based on Salmon quantification method. Supplementary Fig. S4 represents the number of genes implicated in the differential expression, transcript and transcript usage. In this study, we classified the differential expressed genes in three categories, which include (1) changes in the transcript expression (DTE, differential transcript expression), (2) changes in the gene expression (DEG, differential expression gene) and (3) changes in the transcript proportion between AD and control. The comparison of DG with existing gene expression profiles of AD showed that some genes are differentially expressed either in the gene, transcript and transcript proportions or combination of them (Supplementary Fig. S4). We compared the DG with various AD expression profile studies and identified some DGs are not reported in this profile (termed as “novel DG”).

**Figure 2:**
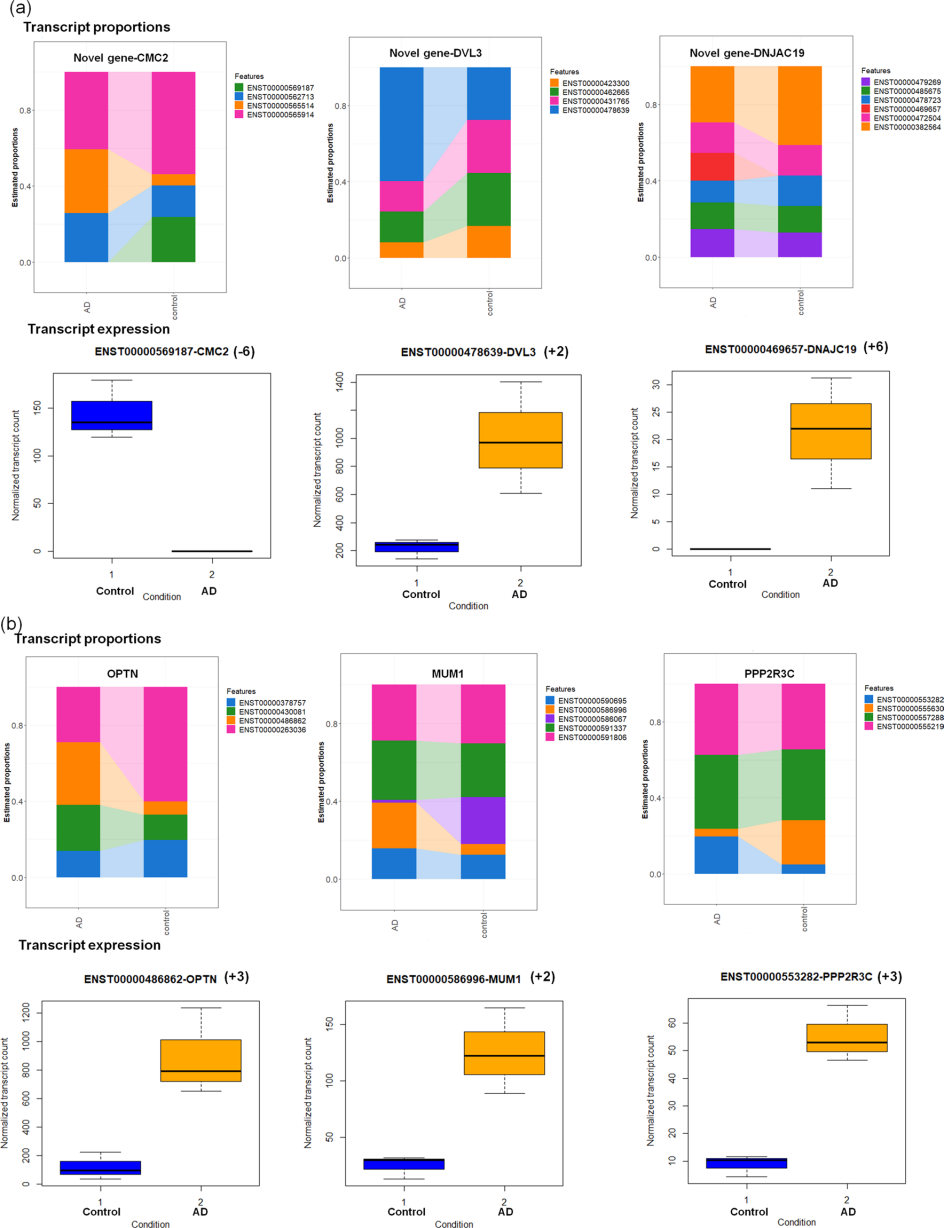
Differential transcript proportion and transcript expression for (a) novel and (b) known AD-associated genes (+upregulation, −downregulation, (+/− number in brackets refer to fold change)).

The differential expressed genes that influence gene/transcript expression and its proportions are presented in Fig. 2.

Figure 2a illustrates the transcript proportions and expression of novel DG genes such as CMC2, DVL3 and DNAJC19. CMC2 gene is widely expressed in brain tissue and plays a vital role in mitochondrial protein import^33^. This gene is involved in the regulation of cytochrome c oxidation and downregulation of this gene may play a role in disrupting energy metabolism. DVL3 is implicated in WNT signaling and upregulation of this gene impacts the downstream signaling cascade. DNAJC19 gene participates in mitochondrial biogenesis, protein transport inside and outside of mitochondria^34^. Upregulation of this gene may influence mitochondrial transport.

Figure 2b represents the transcript proportions and their expression of known AD-associated genes such as OPTN, MUM1 and PPP2R3C. OPTN gene participates in autophagy, vesicle transport, regulation of mitophagy and vasoconstriction (constriction of blood vessels, reducing blood flow)^35^. However, upregulation of this gene may interfere with blood supply to neuron. PPP2R3C gene is involved in immune response, calcium signaling and regulates ATP binding transporter activity. Upregulation of this gene resulted in aberrant calcium signaling and immune response. This study illustrates the genes responsible for mitochondrial morphology, transport, energy metabolism, vasoconstriction and calcium regulation are impacted in AD. Supplementary Table S5 contains detailed information about the differential expression.

**Figure 3:**
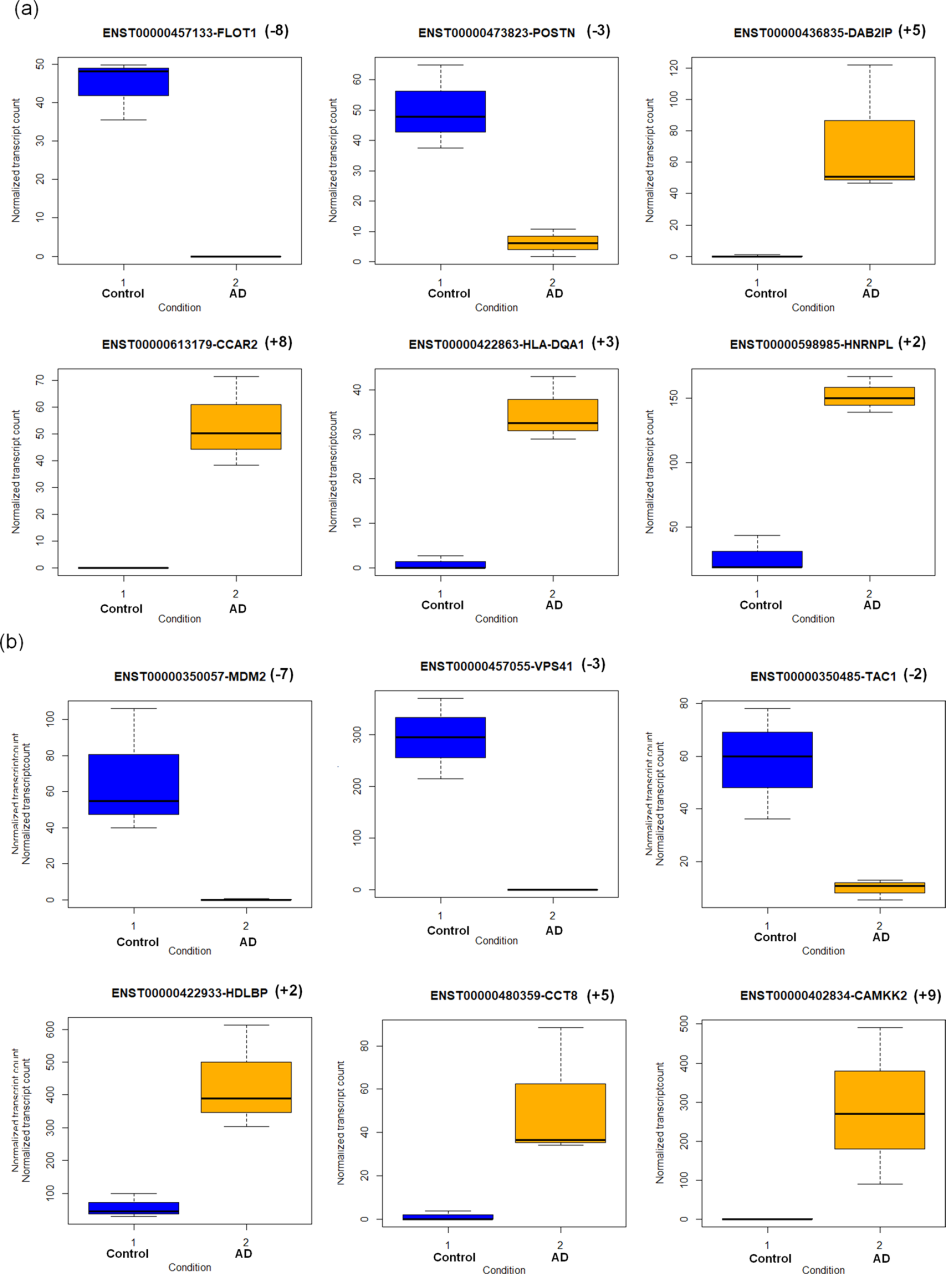
Differential gene and transcript expression for (a) novel and (b) known AD-associated genes (+upregulation, −downregulation, (+/− number in brackets refer to fold change)).

The differential gene and transcript expression of few novel genes (FLOT1, POSTN, DAB2IP, CCAR2, HLA-DQA1 and HNRNPL) and known AD-associated genes (MDM2, VPS41, TAC1, HDLBP, CCT8 and CAMKK2) are shown in Fig. 3.

#### Novel genes

FLOT1 participates in vesicle-mediated transport, cholesterol metabolism and insulin signaling^36^. Downregulation of this gene may influence neurotransmitter transport and potassium channel activity. POSTN gene is associated with cell adhesion, regulation of blood circulation, axonogenesis and enhances neuroprotection^37^. The downregulation of this gene may inhibit its neuroprotective role. DAB2IP gene acts as a negative regulator of vascular endothelial growth factor signaling and angiogenesis^38^. Further, it inhibits endothelial migration. Upregulation of this gene may negatively regulate endothelial cell migration during tissue injury. CCAR2 is involved in apoptosis, autophagy, and expressed in mitochondria. In the oxidative stress condition, this gene maintains mitochondrial integrity by inducing apoptosis^39^. Upregulation of this gene illustrates that this neuronal populationare subjected to tremendous oxidative stress, which in turn induces apoptotic signals. HLA-DQA1 is associated with immune response and is abundantly present in microglia^40^. Overexpression of this gene may result in reactive microglia, which in turn produces neurotoxic cytokines that may cause neuronal death. This depicts that novel DGs are involved in microglial, oxidative stress, immune response, ion channel activity and endothelial functionality dysfunction.

#### Known AD-associated genes

MDM2 gene codes for ubiquitin ligase, which is involved in ubiquitin-mediated protein degradation, angiogenesis and the regulation of vascular endothelial growth factor^41^. Therefore, downregulation of this gene may interrupt endothelial and blood vessel morphology. VPS41 is involved in trafficking misfolded proteins for lysosomal degradation. This gene is downregulated in AD and shows impairment in the protein degradation process. TAC1 gene acts as a potential vasodilator and may dilate blood vessels and increase the blood flow based on neuronal signals. Downregulation of this gene may result in reduced blood supply to neuron.CAMKK2 participates in mitochondrial biogenesis and metabolic homeostasis. CAMKK2 excites AMPA receptors and induces excitotoxicity. Studies show that inhibiting CAMKK2 protects the hippocampus neurons from toxicity^42^. Upregulation of this gene may induce excitotoxicity through NMDA receptor.

### Functional enrichment studies of TF and differentially expressed genes

**Figure 4:**
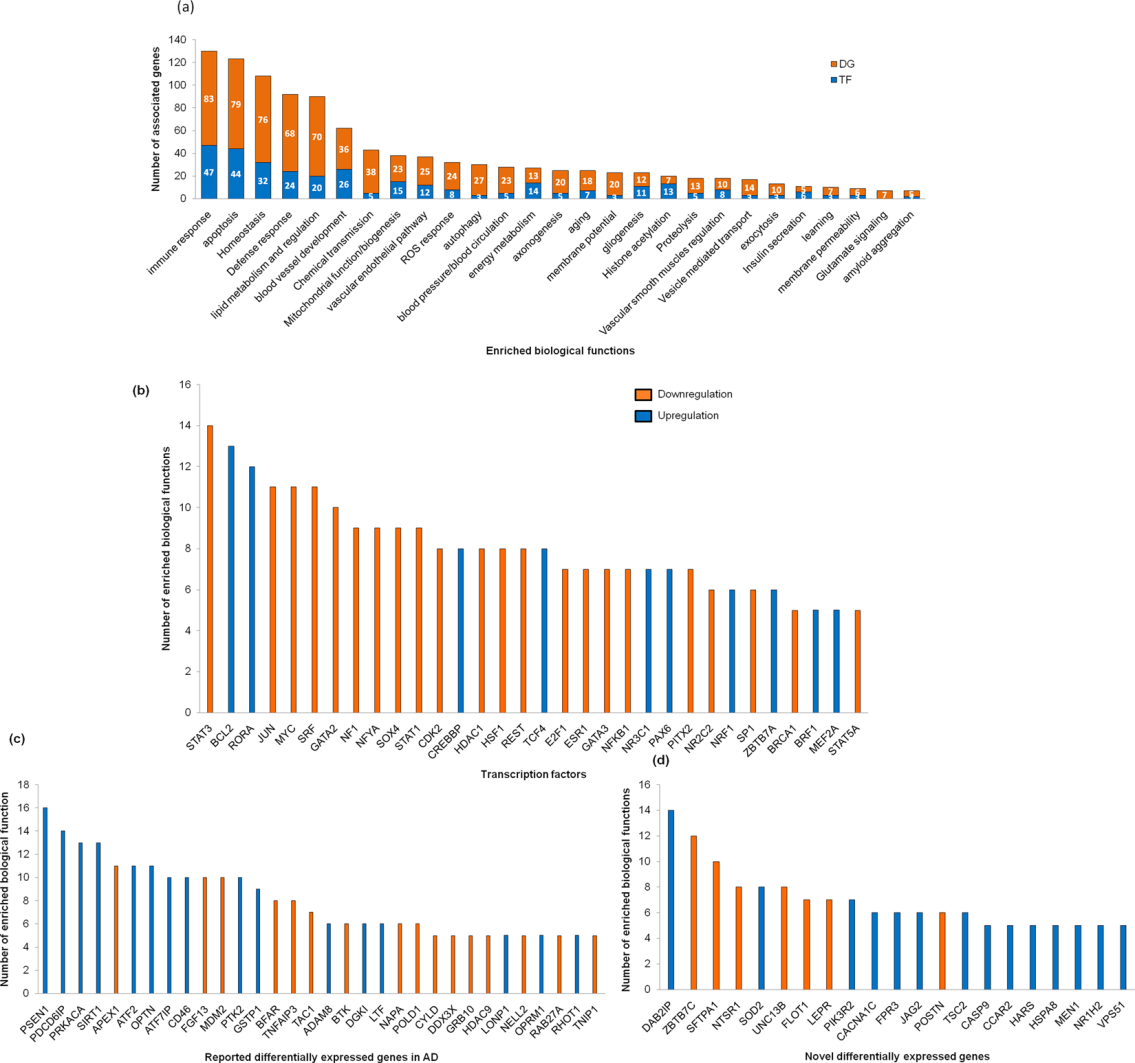
(**a**) Functional enrichment study based on biological functions. The number of enriched biological functions corresponds to (**b**) TFs, (**c**) Known AD-associated gene DG and (**d**) Novel DG (TF-Transcription factor, DG- Differentially expressed genes).

The functional enrichment analysis helps to identify the important biological function in the specified gene set. In this study, we performed enrichment studies of TF, differentially expressed genes as depicted in Fig. 4. Figure 4a represents the number of associated TF/DGs involved in biological functions. We observed that dysregulation of genes are associated with biological functions such as transport, metabolism, oxidative stress, lipid metabolism, ROS response, mitochondrial function, gliogenesis, vascular endothelial pathways, smooth muscle cell regulation, blood vessel morphogenesis, immune response,apoptosis, autophagy and protein degradation. Further, Hippocampus-specific functional module analyses from HumanBase revealed that dysregulation of genes are involved in angiogenesis, lipid transport, glucose transport, mitochondrial function and proteolysis (Supplementary Table S6**)**.

On the other hand, the identified TFs/DGs are involved in multiple biological functions (Fig. 4b). The TFs such as STAT3, BCL2, RORA (novel TF), JUN, MYC, SRF and GATA2 are involved in many biological functions. However, most of the TFs are downregulated in AD and these TFs are associated with immune response, homeostasis, apoptosis, blood vessel morphogenesis and mitochondrial function.

Figure 4c illustrates known-AD associated genes such as PSEN1, PDCD6IP, PRKACA, SIRT1, APEX, ATF2, OPTN, CD46, FGF13 and MDM2, linked in 10 different biological functions. Most of the genes are involved in immune response, apoptosis, lipid metabolism and blood vessel morphogenesis. Figure 4d depicts novel DG such as DAB2IP, ZBTB7C and SFTAP1, which participate in multiple biological functions including apoptosis, immune response, defense response, lipid metabolism and vesicle transport. In summary, most of the dysregulated genes in AD manipulates immune response, apoptosis, defense response, homeostasis and blood vessel morphogenesis. The detailed information about the functional enrichment analysis is depicted in Supplementary Table S7.

### Tissue-specific co-expression and functional interaction networks

We have built a hippocampus-specific co-expression network using differentially expressed genes, VGs, and TFs. From this network, we identified hub genes (degree > 100 nodes) and the results are summarized in Table 2. NF1 is a TF involved in various biological functions and is abundantly expressed in oligodendrocytes, which insulate the neuron via myelin sheath. NF1 gene regulates the RAS signaling pathway, which is essential for cellular growth and survival. Downregulating this gene may disrupt neuronal survival. TSC2 is a novel DG and negatively regulates mTORC1 signaling, essential for lipid synthesis and inhibits autophagy.

**Table 2:** Hub genes identified from hippocampus specific co-expression network.

Gene Name	Type	Gene expression	Degree	Neighborhood Connectivity	Closeness Centrality	Biological function
NF1	TF	DOWN	231	57.05	0.68	Apoptosis, immune response, blood vessel morphogenesis, gliogenesis, Homeostasis, learning, Neurogenesis, vascular endothelial pathway, chemical transmission
TSC2	Novel DG	UP	233	56.16	0.68	Akt/Wnt signaling, autophagy, apoptosis, vesicle transport, mitochondrial regulation
IFNAR2	Novel DG	DOWN	101	58.94	0.53	Akt signaling, immune response
DDX39B	Novel DG	UP	110	63.23	0.55	Regulation of vascular smooth muscle cells
DVL3	Novel DG	UP	109	63.38	0.55	Neurogenesis, Wnt signaling
CAMKK2	Reported DG	UP	223	58.64	0.67	Autophagy, mitochondrial biogenesis, ROS response
TCF3	Reported DG	UP	100	54.21	0.52	Immune response
KCNMA1	variant	UP	237	55.97	0.68	Homeostasis, membrane potential
GRIK2	variant	UP	124	60.98	0.55	Apoptosis, defense response, energy metabolism, exocytosis, membrane potential

TSC2 is upregulated in AD and may inhibit mTORC1 functioning. IFNAR2 is a novel DG involved in cell survival Akt signaling. Downregulation of this gene may further impair neuronal survival. CAMKK2 gene plays an essential role in synapse formation and regulates downstream AMPK signaling cascade. Upregulation of this gene may disrupt the signaling pathways and induce excitotoxicity. GRIK2 and KCNMA1 genes regulate the membrane potential using calcium signals. Upregulation of these genes may impair the firing pattern of the neuron.

**Figure 5:**
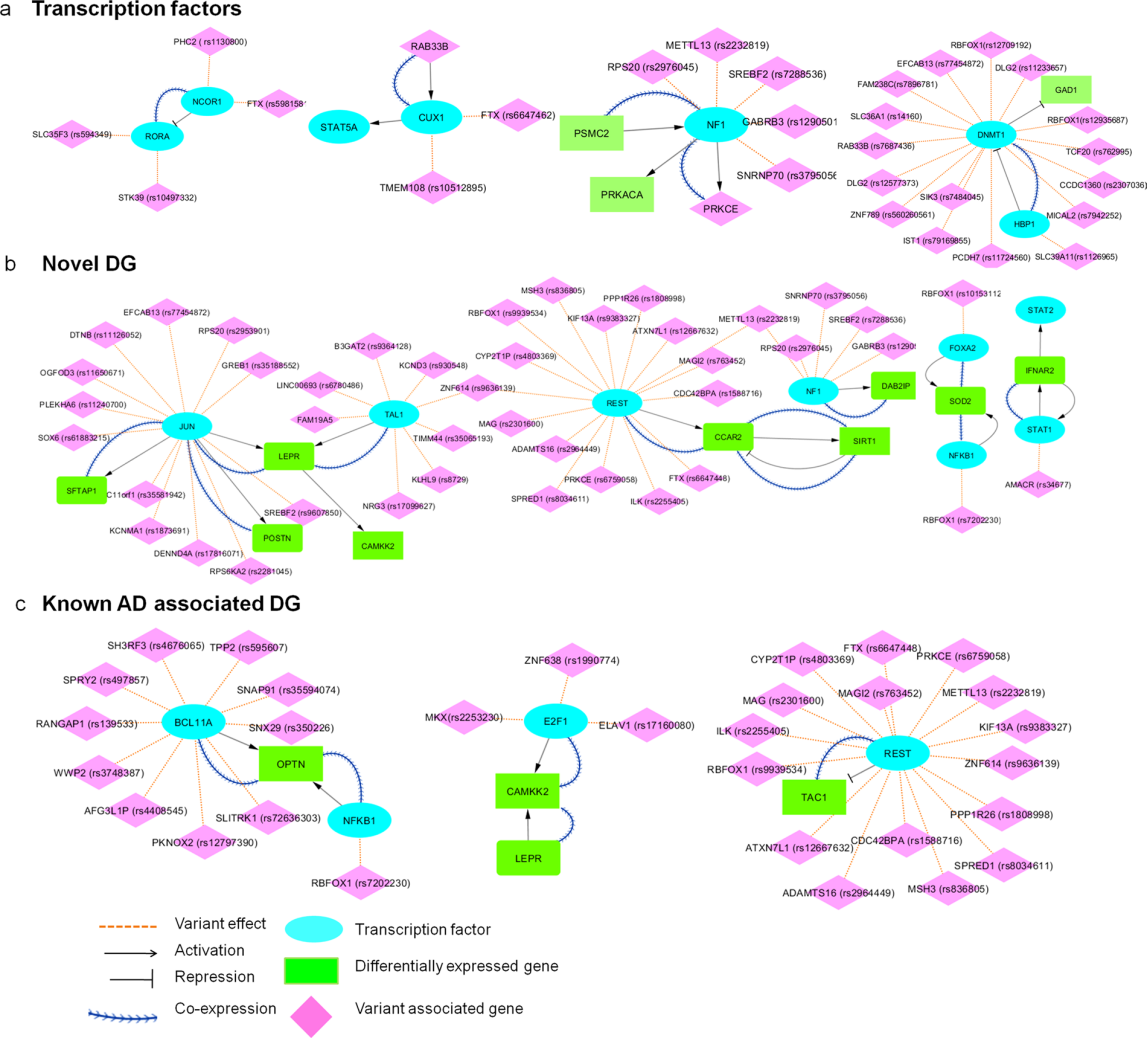
The functional interactions between important differentially expressed genes and TFs.

We constructed the functional interaction network between the DGs, VGs, TF which assists in understanding how the TFs functionally associated to differentially expressed genes. The functional interaction details between the DG and TF genes including the hippocampus co-expression information are presented in Fig. 5.

#### Transcription factors

Figure 5a showed that the NCOR1 TF represses the novel TF, RORA. We found that NCOR1 downregulates and RORA upregulates the gene expression in AD. These genes are co-expressed in the hippocampus and play an essential role in energy and lipid metabolism. NCOR1 TF binding is influenced by novel (rs5981581) and known (rs1130800) variants in FTX and PHC2 genes, respectively. RORA TF binding is affected by STK39 (rs10497332) and SLC35F3 (rs594349) variants. We also found that CUX1, a novel TF is activated by RAB33B. CUX1 and RAR33B are upregulated and co-expressed in the hippocampus, and are involved in the phagocytic activity and immune response. CUX1 TF binding is interrupted by another FTX novel variant (rs6647462) and a TMEM108 (rs10512895) variant. Further, we observed that, PSMC2 (DG) activates NF1 (TF) and these genes are downregulated and co-expressed in the hippocampus. NF1 TF binding is disrupted by METTL13 (rs2232819), SREBF2 (rs7288536) and SNRNP70 (rs3795056) variants. Also our study reveals that HBP1(TF) which represses DNMT1(TF) is downregulated in AD whereas DNMT1 is upregulated in AD. These genes are involved in chromatin remodeling^43^. HBP1 TF binding is interrupted by SLC39A11 variant (rs1126965) and DNMT1 TF binding is affected by 14 GWAS variants and one novel ZNF789 (rs560260561) variant.

#### Novel DGs

Figure 5b depicts JUN (TF) activates the novel DGs POSTN and SFTAP1. These genes are co-expressed in the hippocampus. JUN TF binding is influenced by 12 different variants. POSTN regulates the blood pressure and circulates through endothelial cells. SFTAP1 gene is involved in ROS response and lipid metabolism. Furthermore, the novel DG LEPR is activated by TFs JUN and TAL1 which are affected by 20 GWAS variants. We found that, the novel DG CCAR2 is activated by REST (TF) and it is affected by 14 GWAS variants and one FTX novel variant (rs6647448). CCAR2 activates SIRT1, and SIRT1 represses CCAR2. These genes are upregulated in AD and are implicated in apoptosis and ROS response. In addition, we found that the novel DG SOD2 is activated by TFs, FOXA2 and NFKB1, and these genes are co-expressed in the hippocampus. These TFs are affected by RBFOX1 variants (rs10153112, rs7202230). SOD2 plays an important role in vascular integrity and mitochondrial function. We observed that NF1 (TF) activates the novel DG, DAB2IP and these genes are also co-expressed in hippocampus. DAB2IP and NF1 are involved in blood vessel morphogenesis, immune response, gliogenesis and apoptosis. In addition, the novel DG IFNAR2 and STAT1 (TF) activate each other and are downregulated in AD. STAT1 TF binding is interrupted by AMACR (rs34677) variant. These genes participate in defense response.

#### Known AD associated DGs

TFs NFKB1, BCL11A, activates OPTN (DG) and these genes are co-expressed in hippocampus and are involved in neurogenesis and defense response. NFKB1 and BCL11A TFs binding is interrupted by 11 GWAS variants (Figure 5c). We observed that the CAMKK2 (DG) is activated by E2F1 (TF) and LEPR (DG) and co-expressed in hippocampus. E2F1 TF binding is disrupted by MKX(rs2253230), ZNF638 (rs1990774) and ELAV1 (rs17160080). Furthermore, we identified that TAC1 (DG) is repressed by REST (TF) and is involved in lipid metabolism. Based on this analysis, the predicted differentially expressed genes are connected with TFs either by functional interaction or by co-expression. However, changes in TF binding or expression may affect the target gene regulation and biological function.

### Hypothesis for the energy crisis in hippocampus neuron

**Figure 6:**
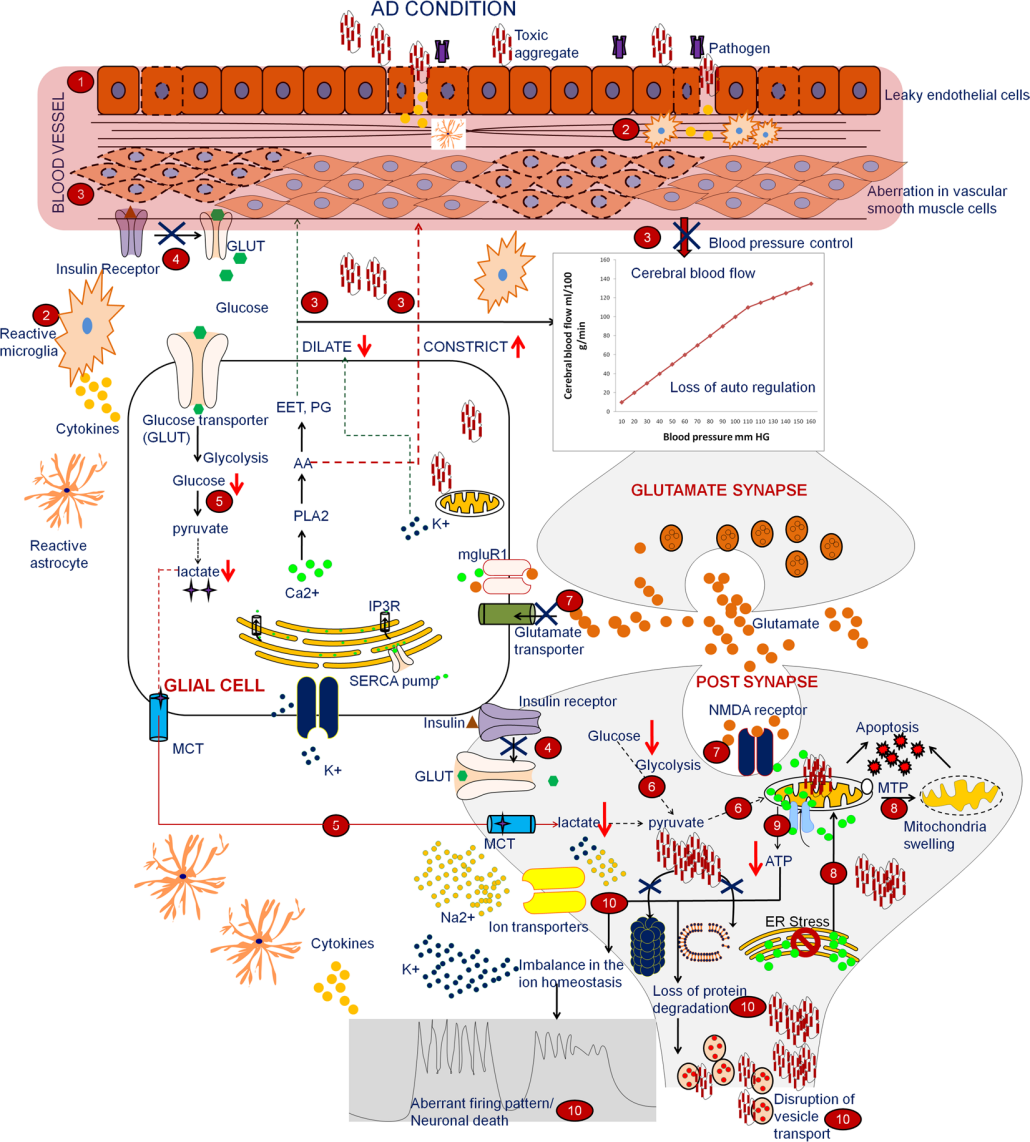
Proposed mechanism for the energy crisis in hippocampus neuron in AD.

The hippocampus neuron needs relatively more energy than other neurons to maintain its structural and functional integrity. These neurons exhibit an increased ROS (Reactive oxygen species) response to oxidative stress and have higher energy demands^3^. In normal condition (Supplementary Fig. S5) the mechanism involves the following steps: (i) The BBB (blood brain barrier) helps to preserve vascular structure by maintaining endothelial cell functional integrity. This saves the brain from toxic substances (ii) The microglia plays a crucial role in the immune response and releases neurotrophic factors that can improve neuronal survival (iii) The vascular smooth muscles regulate the blood pressure based on the neuronal demand, which is communicated through glial cells (iv) The glial cells communicate with neuron as well as vascular smooth muscles and act as a key regulator that controls the cerebral blood flow based on the neuronal activity by regulating vasodilators and vasoconstrictors^44^ (v) After an actionpotential, the increased amount of potassium ions (K^+^) in the extracellular region is taken up by glia (astrocytes) through gap junction and acts as a potential vasodilator, dilating the blood vessels and increasing blood supply. Based on glutamate and K^+^ concentration, glial cells sense neuronal activity and regulate calcium dynamics, which in turn regulates the vasodilator and constrictor (vi) Further, insulin receptors regulate glucose transporters (GLUT) which in turn help to increase the concentration of intracellular glucose (vii) In the glial cells, glucose is converted to lactate through glycolysis. Lactate is transported to the neurons, where it undergoes oxidative phosphorylation. (viii) The neurons mainly depend on oxidative phosphorylation for meeting their energy needs. The neurons utilize this energy to maintain their functional integrity (protein degradation, ion homeostasis, vesicle transport and firing pattern). Hence, the energy supply improves neuronal survival^45,46^. Based on the current study and available literature, we have outlined the reason for the energy crisis in the hippocampus in Fig. 6.

1. In the disease condition, leaky endothelial cells may allow the transport of toxic invaders into the neuron, thus interrupting the vascular dynamics and cerebral blood flow^47^.
2. If tissue injury occurs, the microglia will release neurotoxic substances and inflammatory cytokines, which indirectly affect the vascular structure.
3. Aberrations in smooth muscle cells and imbalance in the vasoregulatory substances affect blood pressure and reduce cerebral blood flow.
4. The insulin resistance influences GLUT and leads to lower intracellular glucose concentration.
5. Disruption in vascular and glial functionality leads to reduced cerebral blood flow, which in turn reduces insulin and glucose metabolism and leads to a deficiency in the lactate supply to the neurons.
6. The reduced supply of lactate and glucose results in diminished oxidative phosphorylation.
7. Aberration in glutamate uptake leads to higher concentration of glutamate in the synapse which upregulates NMDA/AMPA receptor and induces excitotoxicity.
8. This type of excitotoxicity allows increased calcium flux in the ER (endoplasmic reticulum), which in turn increases calcium influx in the mitochondria, leading to MTP formation, swelling and apoptosis.
9. Disruption in mitochondrial morphology and diminished oxidative phosphorylation leads to enormous energy demands in the neurons, resulting in an energy crisis.
10. In order to maintain the firing pattern and ion homeostasis, neurons require higher energy. This type of energy demand may disturb the firing pattern in the neuron. Along with this energy crisis, additional cytotoxic agents (such as protein aggregates) and calcium load further increase the energy demand and subsequently, neuronal vulnerability is higher compared to other neurons.

In the current study, we have deciphered the dysregulation of the novel and reported expressed genes involved in the functional aspects and are listed in Supplementary Table S8. From this, we propose that aberration in vascular structural integrity, gliogenesis are the primary factors responsible for high energy demands, resulting in dysfunctional glucose metabolism, energy imbalance and neuronal vulnerability. This study shows that preserving the vascular integrity and BBB homeostasis may help to shield the hippocampus neuron from the energy crisis and associated energy-related functions. Fluctuation in the cerebral blood flow may be a nearly indicator for energy crisis and cell loss.

## Discussion

The variant analysis for stage 6 of AD showed that the predicted variants are influencing TF binding and epitranscriptomic modifications. The comparison of these variants with GWAS and eQTL studies of AD and other neurological disorders revealed the mechanism behind the energy crisis of AD. The FTX novel variants disrupt 14 different TFs, which are involved in crucial biological functions,including blood vessel morphogenesis, apoptosis, immune response and neurogenesis. From the literature, we noticed that these TFs are differentially expressed in AD. The TFs identified in our study agree with experimentally known observations^32,48^. Further, TFs NR3C1, DNMT1 impacted by the higher number of variants and are differentially expressed. MAN1B1 associated novel variant (rs28373932) disrupts methylation, gene expression and TF binding. The predicted GWAS variants interrupt the binding of novel TFs such as RORA, CUX1 and IRF7. We identified dysregulation of this novel TFs in our differential expression study.

From our differential expression study we have captured 134 literature reported genes, 114 novel differentially expressed genes with isoform resolution. Among them, 25 genes were observed with the changes in both transcript expression and transcript proportions. In some of the cases, we observed the changes in the transcript proportion level, but there is no change in the gene expression level. This may be due to differential transcript usage.

Functional enrichment analysis revealed that novel differentially expressed genes are related to blood vessel morphogenesis (DAB2IP, LEPR, SOD2, SEMA4A, RORA), blood pressure regulation (CACNA1C, CACNA2D, POSTN, SOD2, ZBTB7C), vascular endothelial pathways (DAB2IP, PIK3R2, MAP3K3), vascular smooth cell regulation (SOD2, ZBTB7C), and gliogenesis (RORA, DAB2IP, FPR3), insulin secretion (CACNA1C, CAPN10), mitochondria biogenesis and regulation (MINOS1, SOD2, CCAR2, DNAJC19, LEPR, TIMMDC1), ROS response (CYP2E1, EIF2A, PRKRA, SFTPA1, SOD2, ZBTB7C), lipid metabolism (RORA, DAB2IP, PIK3R2, SFTPA1, SLC16A11, ZBTB7C, NTSR1) and energy metabolism (NTSR1, RORA, ZBTB7C, MEN1, LEPR) are dysregulated in AD.

Tissue-specific co-expression network between TF, DG and VG showed that DVL3, NF1, KCNMA1, IFNAR2 and GRIK2 genes are highly connected with other genes and act as potential hubs. From the functional interaction network, we identified differentially expressed genes regulated by TFs and showed there is an intrinsic functional relationship between them. e.g., Novel DG’s (SOD2, POSTN, RORA, SFTAP1 and LEPR) are involved in energy metabolism and vascular function. SOD2 gene regulated by NFKB1 and FOXA2 TFs. These TFs binding are influenced by RBFOX1 variant. Novel TF RORA influenced by NCOR1 TF and novel variant FTX disrupts the binding of NCOR1. POSTN and SFTAP1 activated by JUN TF and this TF binding affected by KCNMA1 variant. LEPR activated by JUN and TAL1, both of these TFs are influenced by RBFOX1 variant. In summary, we identified the variants from RNA-seq data that may affect the binding of TF. From the functional network, we deciphered the relationship between the predicted TFs and DG. Hence, this study decodes how the variants may disrupt the TF binding which in turn dysregulates the downstream gene expression.

The gene expression and network study from hippocampus neuron provide deep insights to understand the underlying mechanism for the energy crisis in the specific neuronal populations. From this study, we propose that restoration of the BBB, vascular integrity and glial function may rescue the surviving hippocampus neuron from the energy crisis and cell death. Examining the cerebral blood flow in control and disease samples may provide additional information to energy crisis before damaging the energy demanding neuron. Extending this study to single cell RNA level may shed further light on the energy crisis in neurodegenerative disorders.

## Conclusion

The hippocampus neurons need huge energy for its functionality and these neurons are selectively vulnerable in AD pathogenesis. However, the underlying cause for metabolic stress is still unknown. In this study, we performed variant and differential expression profile analysis from hippocampus RNA-seq data. The variant analysis showed that most of the GWAS and novel variants disrupt the TF binding. We identified differentially expressed genes with transcript level resolution. The functional interaction network depicts that there is an intrinsic relationship between TF and differentially expressed genes. The large scale network and functional enrichment studies reveals that during hypoxic condition, these neurons release the inflammatory cytokines, which can be assessed by astrocytes and microglia. The increased cytokine and ROS response may affect the structural integrity of endothelial and vascular smooth muscle cells, which eventually increase the permeability of the BBB that allows toxic invaders inside the brain. These events further enhance the inflammatory response and it impairs the cerebral blood pressure that leads to reduction of regional cerebral blood flow. The communication loss between glia and vasculature influences insulin resistance that additionally induces inflammation and reduces the glucose uptake that leads to glucose hypo metabolism. However, the reduced levels of glucose influence the lactate shuttle, which in turn resulted in diminished oxidative phosphorylation. All these above events impose a greater metabolic stress on this neural population. Along with this energy crisis, an additional stress such as protein aggregation, genetic risk factors and calcium load expands the vulnerability of this neuron in a higher rate compared to others. This shows imbalance between energy supply and demand, which drastically hampers the energy craving neuron and leads to selective neurodegeneration. This study suggests that constant monitoring and restoration of vasculature may secure these neurons before the energy crisis.

## Methods

### Variant analysis

The RNA-seq samples were retrieved from the SRA database and reads were preprocessed to remove the adapter, hexamer contamination, and low Phred quality reads (Q < 20) using Trim Galore^49^. The preprocessed FASTQ files are subjected to spliced alignment with known hg38 genomic annotation using STAR2.6^50^. Details on samples and processed reads are tabulated in Supplementary Table S1.

The duplicates from the aligned files were discarded using Picard Tools and the aligned files were subjected to variant calling using the GATK^51^. For evaluating the variants, we used stringent filtering criteria such as minimum read depth >10, Phred score >30, Ts/Tv > 2.5 and P_adj_ value < 0.05 in order to avoid false positive variant call^52^. We compared the predicted variants with GWAS and eQTL studies from various neurological disorders listed in Supplementary Table S2. The VGs are compared with differential expression profiles of Alzheimer disease, which are obtained from various literature sources, Gene Expression Omnibus (GEO) and the HumanMine database^53^.

This comparison also assists in confirming the variants already reported in GWAS and any novel variants identified from the current analysis. GWAS provide information on the variants predominantly present in the disease population but not on the effect of variants. In this work, we predict the effects of variants in transcription binding and epi-transcriptomic modifications using HaploReg^54^, SNP2TFBS^55^, GWAS4D^56^ and RMBase^57^ databases as well as gene expression using deep learning networks and xQTL analysis^58–60^. The xQTL study comprises 411 control samples from frontal cortex area procured from old aged people, which provides the effect of variants in methylation, histone acetylation and gene expression.

### Differential gene/transcript expression and differential transcript usage analysis

The preprocessed RNA-seq sample reads are subjected to alignment-free transcriptome based lightweight mapping using Salmon^61^. This procedure maps the preprocessed reads with ensembl mRNA hg38 transcriptome sequences and counts the overlapping reads with the given transcripts. We imported the transcript abundance using tximport^62^ in order to identify gene-level expression. We used negative binomial generalized linear and dispersion model from DESeq 2^63^ to identify the differentially expressed genes. We analyzed the variance within and between the biological replicates. Using statistical parameters (False discovery rate < 0.05, minimum fold change |log_2_ FC| > 1) we filtered the differential expression genes/transcripts. Further, using Salmon count data, we performed differential transcript usage using DRIMSeq and stageR^64^ which helps to identify the transcript proportions between two different conditions. This study aids in understanding the differential expression profiles of the gene, transcript and transcript proportions. The overall workflow for variant and differential expression analysis is depicted in Supplementary Fig. S1.

### Network and enrichment analysis

The large-scale network analysis helps to decipher the functional interaction between the given gene set. The functional enrichment, pathway analysis of TFs, DGs and predicted VGs are carried out by InterMine, KEGG, Reactome and ClueGO Cytoscape app^23,65^. We have built hippocampus specific co-expression network, which is obtained from predicted TF, DG and VG using HumanBase^66^. This assists in identifying the potential gene hubs based on various network measures. We constructed a functional interaction network for identifying interactions between DG, VG and TF using ReactomeFI Cytoscape app and TRRUST v2 database^67,68^. TRRUST2 provides regulatory information that includes TF target genes and their functional association. This type of study helps in understanding the interaction between TF and DG.

## Supplementary information

Supplementary information
