# Supplementary material for: Investigating the energy crisis in Alzheimer disease using transcriptome study
Source: Sci Rep. 2019 Dec 6;9:18509. doi: 10.1038/s41598-019-54782-y (PMC6898285; doi:10.1038/s41598-019-54782-y)
Supplement: Supplementary file 1 — Supplementary information [file 41598_2019_54782_MOESM1_ESM.pdf]

Supplementary Information

Investigating the energy crisis in Alzheimer disease using transcriptome study

S. Akila Parvathy Dharshini[1], Y-h. Taguchi[2] and M. Michael Gromiha[1,*]

[1]Department of Biotechnology, Bhupat and Jyoti Mehta School of Biosciences, Indian Institute of Technology Madras, Chennai 600036, Tamilnadu, India.

[2]Department of Physics, Chuo University, Kasuga, Bunkyo-ku, Tokyo 112-8551, Japan.

Table of contents

List of Supplementary Figures	
Supplementary Figure S1	The workflow for variant calling, differential expression and network analysis of RNA-seq Alzheimer data.
Supplementary Figure S2	The number of predicted variants from stage-6 of AD (GATK/STAR).
Supplementary Figure S3	The differential gene expression of (a) Known transcription factors (b) Novel transcription factors affected by predicted variants
Supplementary Figure S4	The number of predicted differentially expressed genes and transcripts - Salmon based transcriptomic quantification
Supplementary Figure S5	Cerebral blood flow and energy functions in normal condition
**List of Supplementary Tables**	
Supplementary Table S1	Datasets used for this study obtained from hippocampus tissue
Supplementary Table S2	GWAS studies of Alzheimer, Parkinson, and Age related Macular degeneration, Amyotrophic Lateral Sclerosis.
Supplementary Table S3	The variant effect on methylation , histone acetylation, expression and transcription factor binding
Supplementary Table S4	The number of predicted variants altering gene expression, methylation, histone acetylation and TF binding
Supplementary Table S5	Differential gene/ transcript expression study
Supplementary Table S6	Hippocampus specific functional module analysis
Supplementary Table S7	Functional enrichment study of predicted variants, TF and differentially expressed genes
Supplementary Table S8	Biological function of differentially expressed genes (novel and known genes)

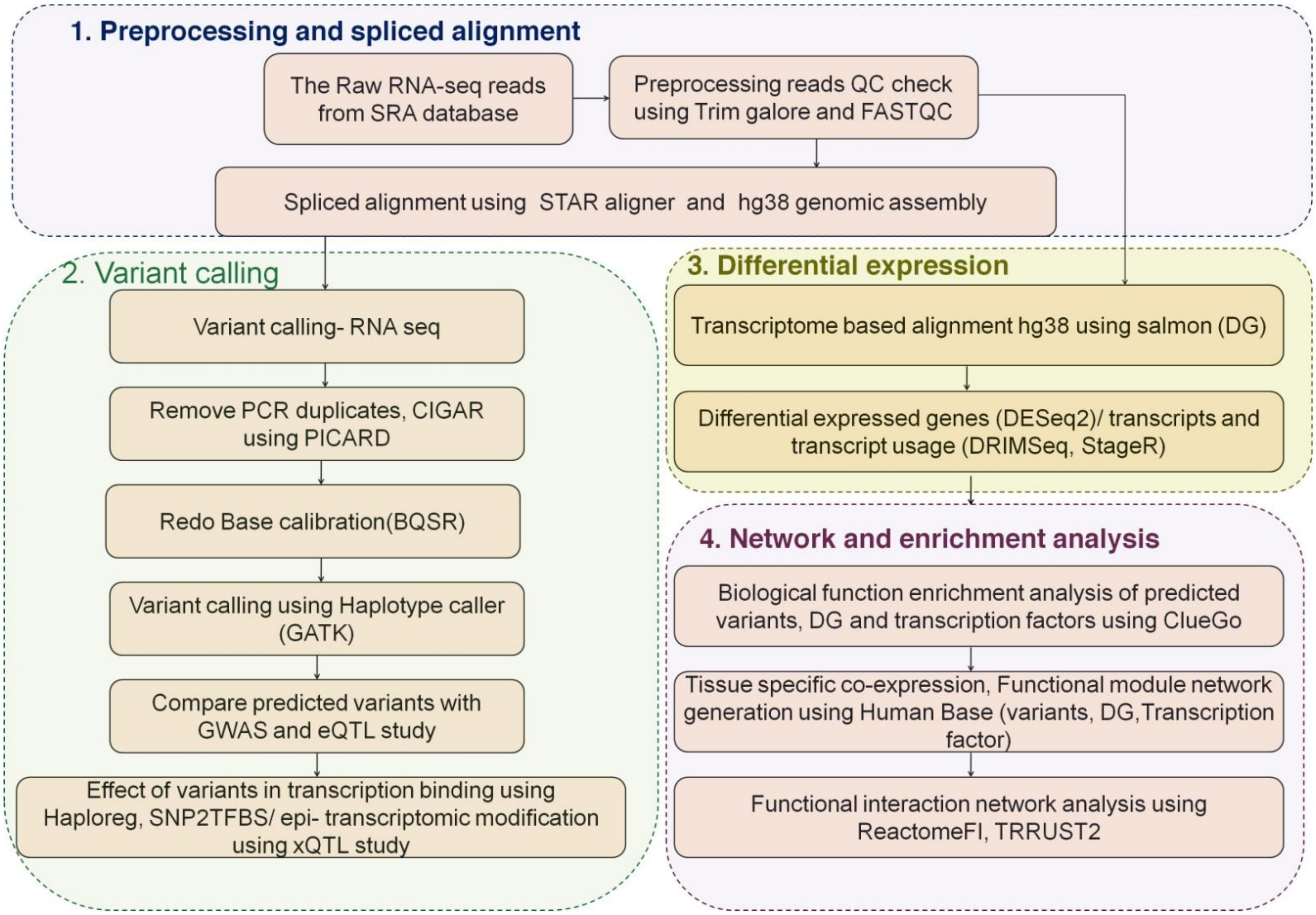

Supplementary Figure S1: The workflow for variant calling, differential expression and network analysis of RNA-seq Alzheimer data.

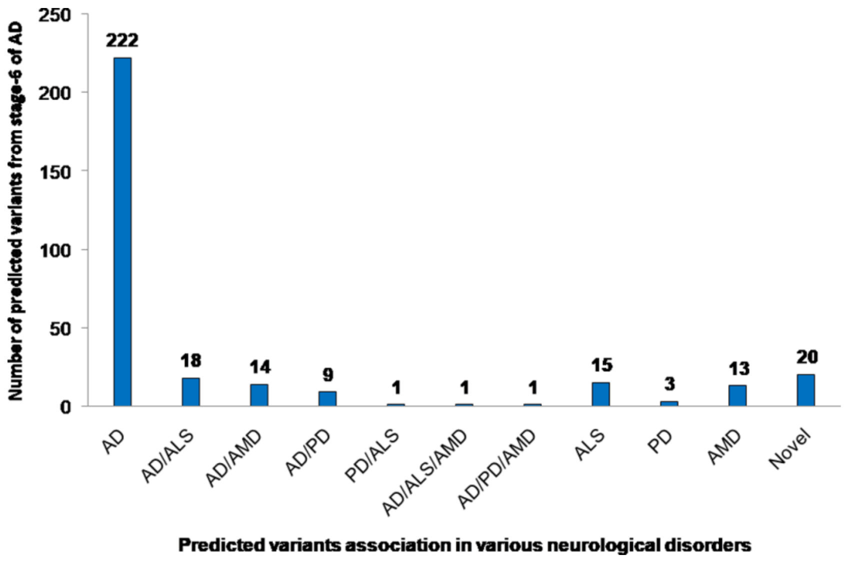

Supplementary Figure S2: The number of predicted variants from stage-6 of AD (GATK/STAR).

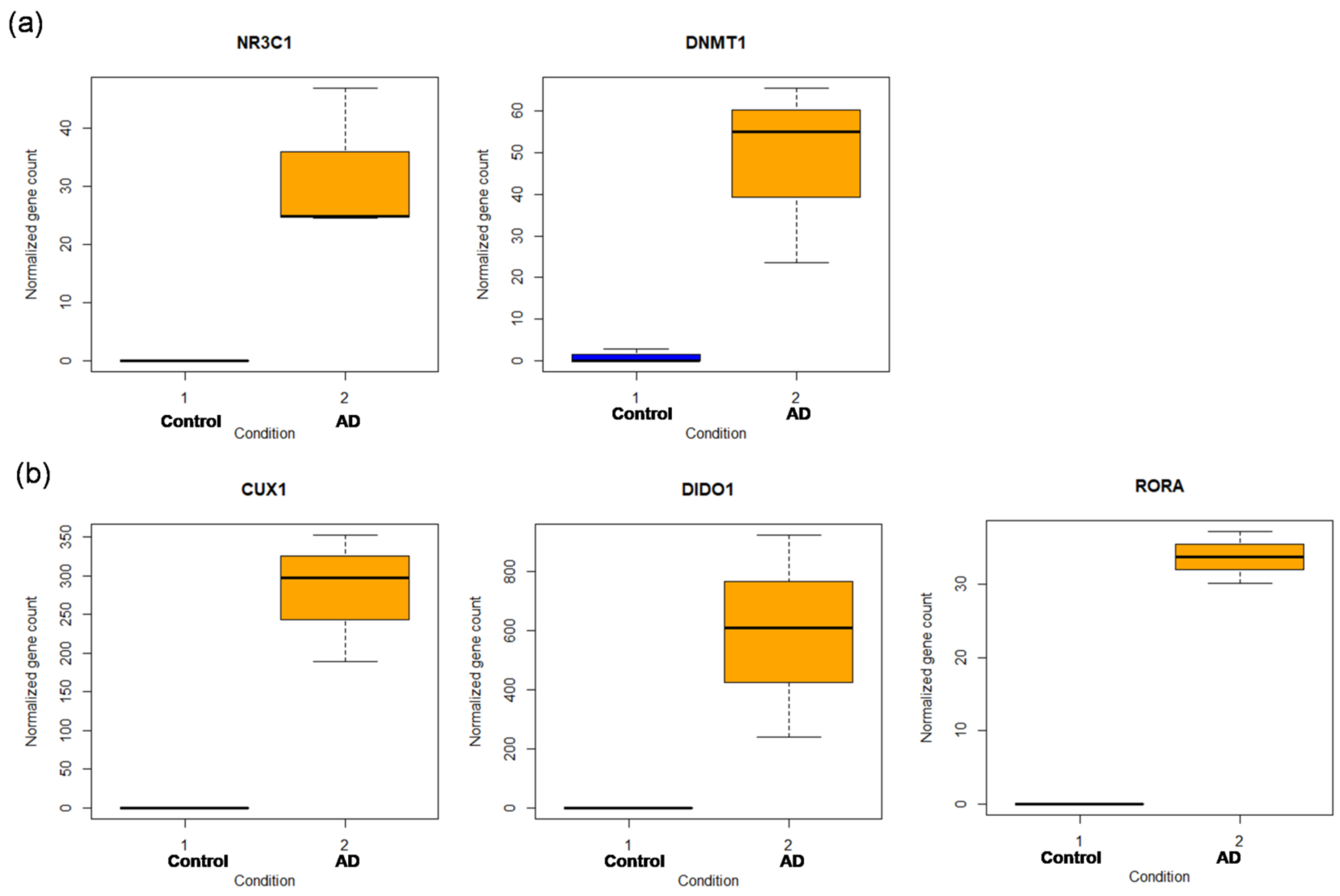

Supplementary Figure S3: The differential gene expression of (a) Known transcription factors (b) Novel transcription factors affected by predicted variants

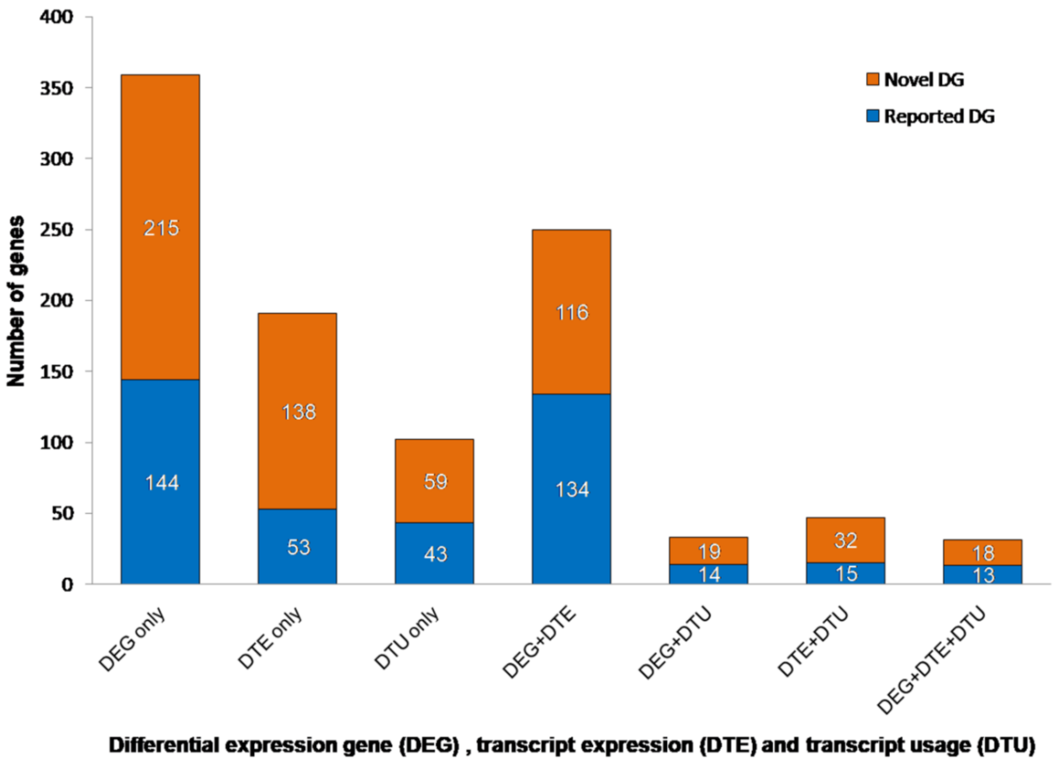

Supplementary Figure S4: The number of predicted differentially expressed genes and transcripts - Salmon based transcriptomic quantification

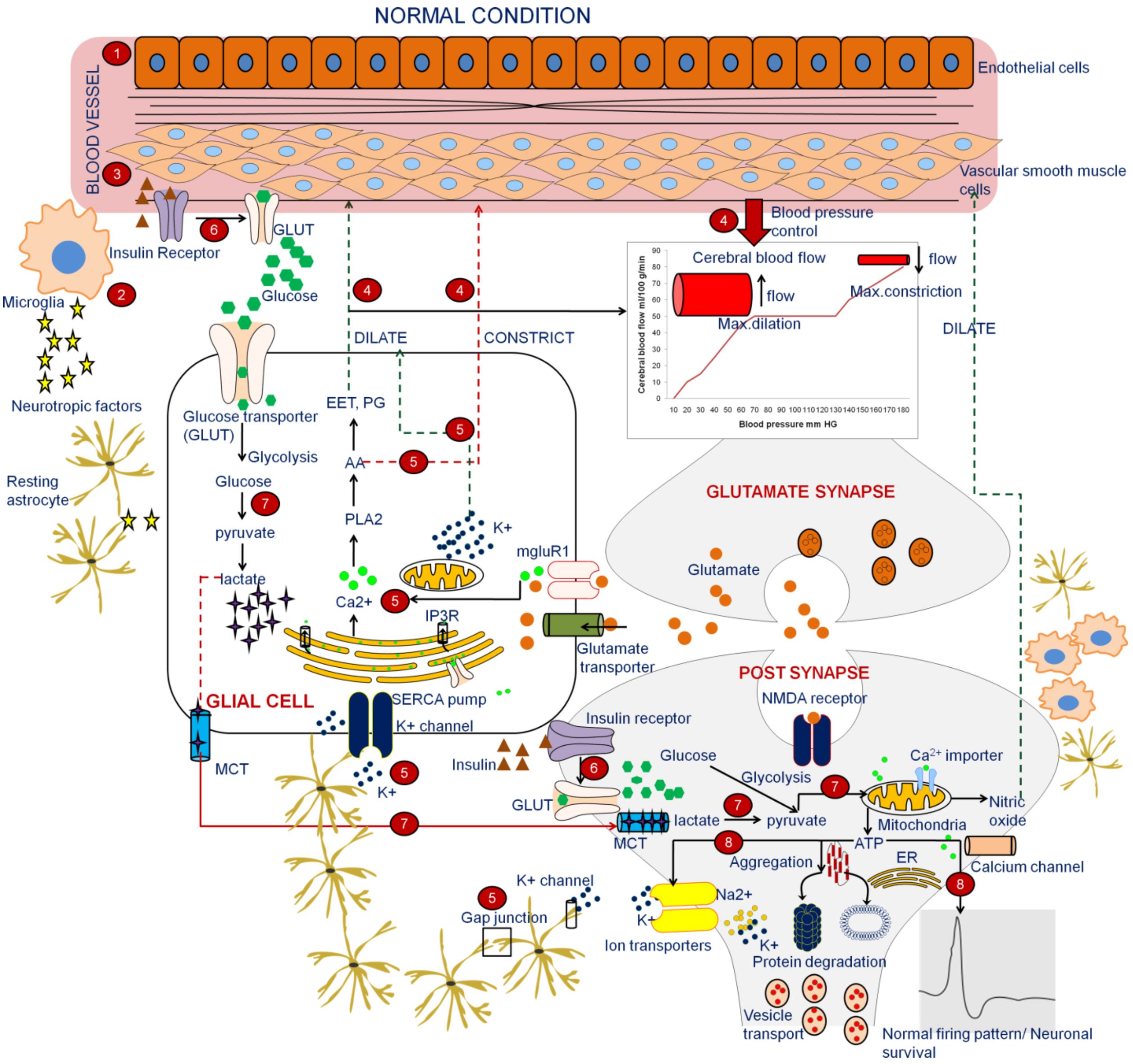

Supplementary Figure S5: Cerebral blood flow and energy functions in normal condition

Supplementary Table S1: Datasets used for this study obtained from hippocampus tissue

SRA ID	Sample	Number reads before filtering	After filtering	Gender	Demised age	Alignment rate	Braak stage
SRR1931816	control	177108169	143571067	Female	77	90.2%	NA
SRR1931817	control	173894719	145425427	Male	83	91.0%	NA
SRR1931818	control	164772379	150652083	Male	90	87.4%	NA
SRR1931819	control	170053030	152216500	Female	85	92.4%	NA
SRR1931812	AD	187514717	168868570	Female	82	91.7%	VI
SRR1931814	AD	178521597	161465641	Male	83	89.8%	VI
SRR1931815	AD	188604801	161247903	Female	82	90.4%	VI

Supplementary Table S2 : GWAS studies of Alzheimer, Parkinson, and Age related Macular degeneration, Amyotrophic Lateral Sclerosis.

Consortium/niagads ID	Source of Sample	Sample size
NG00058	Blood	25,849 control, 14,406 AD
NG00056	Blood	20,474 control, 5813 AD
NG00055	CSF	3146 Control,3154 AD
NG00053	Blood	11,312 control, 8,572 AD
NG00052	Blood	520 control, 1116 AD
NG00050	CSF/plasma	934 control, 1094 AD
dbGap	Blood	4238 PD, 4239 control
AMD gene consortium	Blood	17100 AMD, 60000 control
ALS gene consortium	Blood	12,577 ALS, 23,477 control

AD: Alzheimer disease, PD: Parkinson disease, AMD: Age-related macular degeneration, ALS: Amyotrophic lateral sclerosis

Supplementary Table S3: Predicted variant effect on methylation (mQTL), histone acetylation (haQTL),expression (eQTL) and transcription factor binding

Gene name	Gene expression of variant associated gene	HG38	Ref/Alt	Genomic location	mQTL, haQTL, eQTL	SNP ID	Regulatory elements affected by variant	GWAS/eQTL study	score
RBFOX1	UP	7222542	G/A	intronic	0,1,1	rs12935687	RFX5,	AD	3
CDC27	DOWN	47055097	G/A	intergenic	0,1,1	rs11079748	Nanog,TCF12,MLLT1	AD	3
ACE	DOWN	63488670	G/A	exonic	0,0,0	rs4343	NR2C2	AD	5
ATG7	NA	11330677	T/C	intronic	0,0,0	rs2606750	Nanog,SOX4,MAF	AD	8
MTCH1	UP	36975152	A/G	intronic	0,0,0	rs745506	NR3C1	AD	4
DYNC1I1	UP	95822571	C/G	intronic	0,0,0	rs2706877	USF2	AD	4
ARFGEF1	DOWN	67287549	C/T	intronic	0,0,0	rs35754047	STAT3	ALS	3
ADARB2	UP	1708304	C/A	intronic	0,0,0	rs10903548	SETDB1	AD	3
MMS19	NA	97465981	T/A	intronic	0,0,0	rs2236575	NA	AD	5
TIMM44	DOWN	7935842	A/G	intronic	1,1,1	rs35065193	Nanog,TCF12,TAL1	AD	3
MAG	UP	35295965	C/T	exonic	1,0,0	rs2301600	ESR1,ESR2,REST,NR3C1	AD	0
PHC2	NA	33324367	G/T	UTR3	1,0,0	rs1130800	MZF,NCOR1	AD	6
DUSP12	NA	161751917	C/T	exonic	0,0,0	rs1063178	NA	AD,ALS	6
KCNJ6	UP	37774124	A/G	intronic	0,0,0	rs2835954	HLF	AD	3
CECR7	NA	17044694	A/G	ncRNA_intronic	0,0,1	rs2845394	NFYB	AD,AMD	3
FAM171B	NA	186761611	C/T	exonic	0,0,0	rs13026081	GATA2	AD	5
RNF123	NA	49701512	C/T	exonic	0,0,1	rs6804655	NA	AD	5
CNTN3	NA	74294591	G/A	intronic	0,0,0	rs17012313	NA	AD,ALS	3
SNAP91	UP	83696618	C/A	intronic	0,0,0	rs217288	Bcl6,CCNT2	AD	4
ANKRD18A	NA	38552341	T/C	intergenic	0,0,0	rs4242650	NA	AD	3
MICAL2	UP	12120523	G/A	intronic	0,0,0	rs7942252	DNMT1	PD,ALS	4
SLITRK1	NA	83730563	A/G	intergenic	0,0,0	rs72636303	BCL11A	ALS	3
TIMM44	DOWN	7927167	A/C	UTR3	1,0,1	rs12976850	ESR1,YY1,ZFX,CCNT2	AD	7
HSD3B1	NA	119519050	A/C	intergenic	0,0,0	rs1812256	Maf	AD	3
USP34	NA	61312810	T/C	intronic	0,1,1	rs2600664	NA	AD	3
RPS20	DOWN	56069090	G/A	intronic	1,0,1	rs6474043	NA	AD	4
KCNMA1	UP	77005035	G/A	intronic	0,0,0	rs1873691	JUN,CEBPG,	AD	4
AFG3L1P	NA	89977620	C/T	ncRNA_exonic	1,0,1	rs4408545	BCL11A	AD,AMD	4
NPTX1	UP	80470498	T/G	UTR3	0,0,0	rs4077719	ZEB1	AD	7
OGFOD3	DOWN	82390132	A/C	UTR3	1,0,1	rs11650671	JUN	ALS	5
CYP2T1P	NA	40810075	G/A	ncRNA_exonic	1,0,1	rs4803369	REST	AD	5

SCN1A	UP	166066509	A/G	intronic	0,0,0	rs1461198	HOXA10,HOXA9	AD	4
B3GAT2	NA	71084701	C/T	intergenic	0,0,0	rs9364128	ZEB1,TAL1	AMD	3
UNC5D	NA	35350128	T/C	intronic	0,0,0	rs28388271	MYC,YY1,SRF	AD	4
RPS20	DOWN	56069265	C/A	intronic	1,0,1	rs2953901	JUN	AD	4
KLHL9	UP	21331120	C/T	UTR3	0,0,0	rs8729	SOX1,TAL1,TCF4,TAL1	AD	5
FAM238C	NA	26966822	C/T	intergenic	0,0,0	rs7896781	DNMT1	AD,PD	5
FAM13C	UP	59350010	A/C	intronic	0,0,0	rs11006443	RXRA	AD,AMD	3
SHANK2	DOWN	70578083	G/A	intronic	0,0,0	rs11236708	ESR1,SP1	AD,PD	3
DLG2	UP	83492607	G/A	intronic	0,0,0	rs7127319	KAT2B	AMD	4
CNTN5	DOWN	99061554	A/T	intronic	0,0,0	rs10892735	STAT5A	AD	3
SPRED1	NA	38266634	C/T	intronic	0,0,0	rs8034611	REST	AD	4
RPAIN	NA	5431358	G/A	intronic	0,0,1	rs11650692	ZEB1,SMAD2,SMAD3	AD	4
RAD51L3-I	NA	35009895	T/G	ncRNA_exonic	0,0,1	rs3744358	HBP1	AMD	8
SLC39A11	NA	72646637	A/T	UTR3	0,0,0	rs1126966	NA	AD	5
NRXN1	UP	50385732	C/A	intronic	0,0,0	rs6722443	HOXA1	AD	4
MXD1	NA	69935402	T/G	exonic	0,0,0	rs2228183	NA	AD	6
SCN1A	UP	166065513	C/A	intronic	0,0,0	rs13397208	EZH2	AD	3
ARHGEF3	UP	56727622	A/G	UTR3	0,0,0	rs808	NR3C1	AD	5
GUCY1B3	UP	155791879	G/A	intronic	0,0,0	rs7661128	RXRA,IRF5	AD,AMD	3
ZBTB16	UP	114208485	C/G	intronic	0,0,0	rs669024	NR3C1,YY1	AD	5
SNAPC1;SY	NA	61977460	C/A	intergenic	0,0,0	rs2353857	RAD21,STAT6,NR2C1	AD	4
ZNRF1	NA	75024657	A/G	intronic	0,0,0	rs35934181	NA	AD	4
ANKRD11	NA	89372862	C/T	intronic	1,0,0	rs744327	SP1	AMD	4
METTL13	DOWN	171786042	G/A	exonic	1,0,0	rs2232819	REST,NF1	AD	0
CDC42BPA	UP	227245989	A/G	intronic	0,0,0	rs1588716	NR3C1, REST	AD	3
TCF20	NA	42276118	A/G	intronic	0,0,1	rs762995	IRF4,DNMT1	AD	10
ADAMTS1(	NA	5414687	G/T	intergenic	0,0,0	rs2964449	BCL3,REST	AD	3
MAGI2	UP	78547649	A/G	intronic	0,0,0	rs763452	MYC, REST, NRF1,SETDB	AD	4
TRIB1	DOWN	125370792	T/C	intergenic	0,0,0	rs117596499	Gfi1b	ALS	3
C11orf1	NA	111885562	C/T	intergenic	0,0,0	rs35581942	JUN,BCL2,RXRA	AD	5
PHC2	NA	33324895	G/A	exonic	1,0,0	rs11554674	NA	AD,ALS	6
MYT1L	UP	2802425	T/G	intergenic	0,0,0	rs6712409	PAX6	AD	3
LINC00903	NA	116564717	G/A	ncRNA_intronic	0,0,0	rs9862002	NR3C1, YY1,NR2C1	AD	4
PLXNA4	NA	132357439	A/G	intronic	0,0,0	rs1863015	NA	AD	3
UNC5D	NA	35307303	T/C	intronic	0,0,0	rs4739400	HOXA5,SRF	AD	3
DLG2	UP	83521771	T/G	intronic	0,0,0	rs12577373	DNMT1	AMD	5
OPCML	UP	133451936	C/A	intronic	0,0,0	rs6590703	SMARCA1	AD	3
RBFOX1	UP	7227946	G/T	intronic	1,0,0	rs12709192	DNMT1	AD	4
FAM192A	NA	57174958	T/G	intronic	0,0,0	rs56089342	GATA3	AD	4

CDH13	NA	83786436	C/G	intronic	0,0,0	rs692600	NA	ALS	3
RABEP1	NA	5379468	C/T	intronic	0,0,1	rs8081481	NA	AD,ALS	5
RPRD2	UP	150433973	A/C	intronic	0,0,0	rs9436112	NFYA	AD,ALS	3
TCF20	NA	42256068	G/A	intronic	0,1,1	rs134869	NA	AD	3
LINC00693	NA	28672296	A/G	ncRNA_intronic	0,0,0	rs6780486	TAL1,GATA2	AD	3
DYNLL1	UP	120471890	C/T	intronic	0,0,1	rs3759396	ZEB1,SMAD2, SMAD3	AD	5
RABEP1	NA	5341599	G/C	intronic	0,0,1	rs9907108	HOXA9	AD,ALS	3
TIMM44	DOWN	7935116	C/T	exonic	1,0,1	rs11542189	NA	AD,AMD	4
WBP2NL	NA	42035242	G/T	intergenic	0,1,1	rs133351	KAT2B	AD	3
SH3YL1	DOWN	256278	T/C	UTR5	0,0,1	rs4455191	CTBP1	AD	5
CADM2	NA	85225405	G/C	intronic	0,0,0	rs7625890	CTBP1	AD	3
LSAMP	NA	115924430	A/C	intronic	0,0,0	rs10511352	NA	AD	3
GRIK2	UP	102016732	T/A	intronic	0,0,0	rs13218403	Bbx,HSF1,ELF1	AD,ALS	3
NRG3	NA	82218939	A/G	intronic	0,0,0	rs17099627	TAL1	AD,ALS	3
DICER1-AS	NA	95179683	G/T	ncRNA_exonic	0,0,0	rs14042	NA	AD	5
SPG7	NA	89529698	C/T	intronic	1,0,1	rs3803676	HEY1	AD,AMD	5
RABEP1	NA	5379466	C/T	intronic	0,0,1	rs8081480	HOXA9	AD,ALS	4
KCND3	UP	111964942	C/G	intronic	0,0,0	rs930548	TAL1	AD,ALS	4
ERVH48-1	NA	42919268	T/G	UTR5	0,0,1	rs2187247	ESR1, ESR2,NR3C1	AD,PD,AN	3
ELMOD3	NA	85389635	T/C	intronic	0,0,0	rs17026285	Ascl2,HOXA10,Pou6f1	AD	5
KAT2B	UP	20150563	T/A	intronic	0,0,1	rs3804570	NR2C2	AD	3
CHN2	UP	29513418	T/C	UTR3	0,0,0	rs17158153	Foxl1,SMARCA1	AD	7
LOC72874	NA	150410649	A/G	ncRNA_exonic	1,0,0	rs1048407	NA	AD	4
CAMK1D	NA	12379292	A/G	intronic	0,0,0	rs4747979	NA	AD	4
MVK	NA	109576074	G/A	exonic	1,0,0	rs7957619	NA	AD	0
SPRY2	UP	80343392	T/C	intergenic	1,0,0	rs497857	BCL11A	AMD	5
BCL11B	UP	99225456	G/C	intronic	1,0,0	rs8021454	SETDB1	AD,AMD	6
TIMM44	DOWN	7930402	G/A	intronic	1,0,1	rs34629355	GLI1	AD	3
LINC00665	NA	36322182	A/G	ncRNA_exonic	0,0,1	rs2972632	HEY1	AD	3
ZNF283	NA	43842438	T/A	intronic	0,1,1	rs1994414	NR3C1,Rad21,YY1	AD	3
PRICKLE2-A	NA	64197444	G/A	ncRNA_intronic	0,0,0	rs62251775	FOXA2,PBX1,SRF	AD	3
ARHGEF28	NA	73849020	A/G	exonic	0,0,0	rs2973568	NR3C1	AD	5
SLC36A1	NA	151492234	C/T	UTR3	0,0,0	rs1175	DIDO1	AD	6
FLNC	NA	128830784	C/T	exonic	1,0,0	rs3734972	ZBTB1	AD	7
MSRA	UP	10372534	T/A	intronic	0,0,0	rs7842443	HSF1,PAX5,IRF5	AD	3

RUSC2	UP	35557699	G/T	intronic	0,0,0	rs1890590	PAX5	AD	4
PGAP2	NA	3826063	A/G	UTR3	0,0,0	rs1055640	NA	AD	6
SRRM4	NA	119131791	G/A	intronic	0,0,0	rs7301936	ELF1,Ets1,smad3	AD,PD	4
RBFOX1	UP	6167116	G/A	intronic	0,0,0	rs7202230	NFKB1,EZH2	AD	3
RBFOX1	UP	7242194	G/C	intronic	0,0,0	rs12929041	HEY1	AD	3
PSMC3IP	UP	42573638	G/C	intronic	1,0,1	rs2292752	SIX5	AD,AMD	4
MAP3K14	NA	45284124	G/A	intronic	0,0,0	rs2074292	EWSR1,MZF1	AD	3
PLEKHA6	NA	204227199	A/C	intronic	0,0,0	rs4581328	Ascl2	AD,ALS	3
ACP1	DOWN	276942	A/G	intronic	0,0,1	rs7573495	ZEB1	AD	6
PRKCE	DOWN	45773347	A/G	intronic	0,0,0	rs6759058	BCL3,REST,HDAC1	AD,AMD	4
SH3RF3	NA	109133919	G/A	intronic	1,0,0	rs4676065	Ets1,GATA6,ESR1,Klf4,RREB1,BCL11A	AD	3
MTURN	NA	30205297	T/C	intergenic	0,0,0	rs4719997	CREBBP	AD	3
CCDC136	NA	128814483	C/A	intronic	0,0,0	rs2307036	DNMT1	AD	3
RBM23	UP	22905653	C/T	exonic	0,0,1	rs2295682	CUX1	AD	5
WWP2	NA	69940643	A/G	UTR3	1,0,1	rs3748387	RREB1,BCL11A	AD	9
LUZP1	UP	23140568	G/A	intronic	0,0,0	rs74902245	NR3C1,SETDB1	AD	4
RANGAP1	NA	41281210	T/C	intronic	0,0,0	rs139533	Gfi1b,Rad21,BCL11A	AD	5
AFF3	NA	99623734	G/T	intronic	0,0,0	rs34632628	Smad3,YY1	AD	3
POLR2D	NA	127845111	C/T	intergenic	0,0,0	rs77082794	ESRRA	AD	4
IL17RC	NA	9928474	C/T	exonic	0,0,1	rs279548	NA	AD	6
TMEM108	NA	133297060	C/T	intronic	0,0,0	rs10512895	PAX5,CUX1	AD,ALS,A	3
TMED11P	NA	1121659	A/G	ncRNA_intronic	0,0,1	rs28660089	STAT5A	AD	3
MCUB	DOWN	109543296	G/A	intergenic	0,0,0	rs6533433	GATA2	AD	4
PDE4D	NA	59310090	G/C	intronic	0,0,0	rs11959132	HNF4A	AD	4
MAGI2	UP	78062814	G/A	intronic	0,0,0	rs3807761	HNF4A	AD	5
RALYL	UP	84222419	C/G	intronic	0,0,0	rs10504795	PAX8	AD,PD	3
RPL13P5	DOWN	6884494	C/G	ncRNA_exonic	0,0,0	rs12817264	ELF1	AD	6
PLEKHA6	NA	204221284	C/G	UTR3	0,0,0	rs11793	CTBP1	AD,ALS	6
SLC23A2	NA	4889747	C/T	intronic	0,0,0	rs1776957	NA	AD	3
FAM19A5	NA	48748288	G/A	intronic	0,0,0	rs9628480	Ascl2,LMO2,TAL1	ALS	3
DTNB	UP	25432522	C/G	intronic	0,0,0	rs11126052	JUN,GATA3,MLLT1	AD	4
ATG7	NA	11392906	A/G	intronic	1,0,0	rs6792718	GATA2,CCNT2,GATA6,HMGN3,PAX3	AD	5
TMEM108	NA	133255869	A/G	ncRNA_intronic	0,0,0	rs4854705	MXI1,PAX3,pitx2,GATA2	ALS	3

PCDH7	UP	30819413	A/T	intronic	0,0,0	rs6837246	IRF3,EZH2	AD	8
CSMD1	NA	4763325	T/A	intronic	0,0,0	rs1676963	Gbx1,SMARCA1	AD	3
FRA10AC1	NA	93683931	C/G	intronic	0,0,1	rs8181435	NA	PD	3
FBXO33	NA	39401813	T/C	exonic	0,0,0	rs7156962	TAF1	AD	6
RBFOX1	UP	6848413	A/G	intronic	0,0,0	rs6500855	NA	AD	3
IST1	NA	71916298	A/G	intronic	0,0,0	rs79169855	DNMT1	AD	4
RAB4B-EG	NA	40807297	C/A	ncRNA_intronic	1,0,1	rs3736329	SMC3	AD	5
ZNF638	UP	71341076	A/G	intronic	0,0,0	rs1990774	smad1,E2F1	AD,AMD	3
SND1	DOWN	128080871	T/C	intronic	1,0,0	rs322826	HMGN3	AD	3
MKX	NA	27675541	G/A	exonic	0,0,0	rs2253230	E2F1	AD	5
GALNT18	NA	11286364	A/C	intronic	0,0,0	rs17348760	Pou6f1	AD	3
PICALM	UP	86067882	C/T	intronic	0,0,0	rs544458	SOX1,SMARCA1	AD	6
RBFOX1	UP	6576498	T/C	intronic	1,0,0	rs9939534	REST	AD	3
SHISA9	NA	12954505	G/C	intronic	0,0,0	rs4780485	NR3C1,PBX1	AD	5
MED12L	NA	151272254	T/G	intronic	0,0,0	rs7653603	GATA2	PD	3
PCDH7	UP	30888720	C/A	intronic	0,0,0	rs11724560	RFX5,STAT6,DNMT1	AD	3
ARHGAP26	DOWN	143208247	T/G	intronic	0,0,0	rs2398617	ZBTB7A	AD	5
KIF13A	UP	17817004	C/G	intronic	0,0,0	rs9383327	BCL6,ELF1,REST,YY1	AD	4
MTCH1	UP	36975203	T/C	intronic	0,0,0	rs745505	GATA2	AD	3
UNC5D	NA	35331145	T/C	intronic	0,0,0	rs12550658	PAX5,GATA2	AD	3
RPS20	DOWN	56073255	T/C	exonic	0,0,0	rs1050403	SRF	AD	0
NRG3	NA	82760120	G/T	intronic	0,0,0	rs2644212	Ets1,SIX5	AD	3
DLG2	UP	83479322	G/A	intronic	0,0,0	rs11606666	RXRA,	AMD	4
FRMD5	NA	44075092	A/C	intronic	0,0,0	rs8038096	MEF2A	AD,AMD	4
MBP	UP	77073856	C/T	intronic	0,0,0	rs7233242	NFYB	AD	4
ADGRL2	NA	81589337	T/C	intergenic	0,0,0	rs6659297	HDAC1	AD	3
MAEA	NA	1327180	A/G	intronic	0,0,1	rs59810282	RAD21	AD	3
UVSSA	NA	1358897	A/G	intronic	1,0,1	rs1882099	NA	AD	3
AMACR	NA	33998663	C/A	exonic	0,0,1	rs34677	STAT1,STAT5A	AD	0
MCPH1-AS	NA	6616194	A/G	ncRNA_exonic	1,0,0	rs2959799	SOX15,SRF	AD	4
DPP7	NA	137111750	G/A	exonic	0,0,1	rs13631	NA	AD	0
ARRB1	NA	75333754	T/C	intronic	0,0,0	rs616191	IRF5	AD	5
SIK3	UP	117076174	C/T	intronic	0,0,0	rs7484045	DNMT1	AD	3
SREBF2	UP	41839348	C/T	intronic	0,0,1	rs9607850	JUN,HMGN3,NFE2	AMD	4
ARMC8	NA	138252065	G/A	intronic	0,0,0	rs7631734	NA	AD	5
CDH18	UP	19702446	G/T	intronic	0,0,0	rs62351330	BCL2	AD	3
SNX9	NA	157882556	G/A	intronic	0,0,0	rs2746219	NA	ALS	3
RPS6KA2	UP	166490910	A/G	intronic	0,0,0	rs2281045	JUN	AD	5

FAM185BP	NA	77122040	G/A	ncRNA_exonic	0,0,0	rs3095481	RAD21,DIDO1	AD	6
FOCAD	NA	20902720	A/C	intronic	0,0,0	rs7849011	NA	AD	4
NRG3-AS1	NA	82229336	A/G	ncRNA_intronic	0,0,0	rs17095111	NA	AD,ALS	3
RNLS	NA	88583080	C/G	exonic	1,0,0	rs2296545	HOXA1	AD	0
OPCML	UP	133454191	A/G	intronic	0,0,0	rs6590704	NA	AD	3
SLC39A11	NA	72646651	C/G	UTR3	0,0,0	rs1126965	HBP1	AD	5
ELAVL1	DOWN	7959715	G/A	UTR3	0,0,1	rs17160080	E2F1	AD	7
PPFIA4	UP	203057463	A/G	intronic	1,0,0	rs17461925	GATA2	AMD	3
SREBF2	UP	41847614	T/C	intronic	0,0,1	rs7288536	NF1	AD	5
VSNL1	UP	17596457	C/T	intronic	0,0,0	rs2710691	BCL3	AD	4
FMNL2	NA	152393992	G/T	intronic	0,0,0	rs6721741	NA	AD	3
TMEM108	NA	133268520	A/G	intronic	0,0,0	rs56940649	FXR1,FOXL1,NR3C1,NRF1,KAT2B	ALS	3
GAB1	NA	143371900	G/A	intronic	0,0,0	rs6537155	NA	AD	4
MSH3	DOWN	80679672	G/A	intronic	0,0,0	rs836805	REST	ALS	4
SLC36A1	NA	151491719	T/C	UTR3	1,0,0	rs14160	AScl2,DNMT	AD	6
DDX56	NA	44573154	T/C	intronic	0,1,0	rs217372	NA	AD	5
PTPRD	UP	10391604	A/C	intronic	0,0,0	rs10756031	Alx4,HMGN3	AD	3
NRG3	NA	82222302	A/G	intronic	0,0,0	rs17099635	NR3C1,SOX14,SOX15,sox	AD,ALS	3
LMO7	NA	75806106	C/T	intronic	0,0,0	rs17065074	NA	AD,PD	8
LINC00662	NA	27777033	C/A	intergenic	0,0,0	rs12462571	PAX6, PAX8,HOXA1	AD	3
ACP1	DOWN	266895	C/T	intronic	1,0,1	rs10171043	IRF1,MEF2A	AD	5
LINC0125(	NA	2802430	T/A	intergenic	0,0,0	rs6712407	IRF4,PAX5,PAX6,STAT5A	AD	3
TMEM108	NA	133070515	A/C	intronic	0,0,0	rs12493576	JMJD6	AD	3
LMO7	NA	75807964	C/T	exonic	0,0,0	rs2241913	TCF4,RXRA	AD,PD	0
POP5	NA	120579636	A/G	intronic	1,0,0	rs563920	NA	AD	4
PPCDC	DOWN	75054419	T/C	intergenic	0,0,0	rs7163636	Ets2,HDAC1	AD	4
CDH13	NA	83788207	A/G	intronic	0,0,0	rs466873	NR3C1,SOX2,TCF4	AD	3
NETO1	NA	72605866	C/G	intergenic	0,0,0	rs12962382	PAX5	AD	3
SNRNP70	NA	49107156	C/T	intronic	0,0,0	rs3795056	NF1	AD	4
NFYC	DOWN	40716738	A/G	intronic	0,0,0	rs12034952	NR3C1,GATA	AD	4
ASAP2	NA	9313117	C/T	intronic	1,1,0	rs11892025	SETDB1,YY1	AD	3
PKP4	NA	158478501	A/G	intronic	0,0,0	rs79077595	NA	AD	3
PART1	NA	60523279	T/A	ncRNA_exonic	0,0,0	rs4081739	NA	AD	5
HIVEP2	UP	142835226	A/T	intronic	0,0,0	rs198645	SOX2,SOX14,SOX15,SOX18,SOX9,TCF4	AD	4
UNC5D	NA	35356803	G/C	intronic	0,0,0	rs4739302	NA	AD	3
RPS20	DOWN	56071833	T/C	intronic	1,0,1	rs2953916	NA	AD	4

DLG2	UP	83517246	C/T	intronic	0,0,0	rs7127859	NA	AMD	4
PKNOX2	UP	125261319	A/G	intronic	0,0,0	rs12797390	BCL11A	AD	3
NAV1	NA	201661389	G/A	intronic	0,0,0	rs724221	NA	AD	3
CDC42BPA	UP	227311892	T/C	intronic	0,0,0	rs11804709	IRF5	AD	4
RAPH1	NA	203450671	G/A	intronic	0,0,0	rs11679740	NA	AD	4
ACTR1A	DOWN	102483129	G/A	intronic	0,0,0	rs2296581	NFYA	AD	8
PLEKHA6	NA	204226828	T/C	intronic	0,0,0	rs11240700	JUN	AD,ALS	3
TCF20	NA	42284794	C/T	upstream	1,1,1	rs86669	GATA2	AD	3
SLC6A6	NA	14486520	T/C	UTR3	0,0,0	rs2341985	SRF,CTBP1	AD	5
PPP1R26	UP	135485811	T/C	exonic	1,0,0	rs1808998	REST	AD	0
RBFOX1	UP	7192406	T/G	intronic	0,0,0	rs12930176	HNF4A	AD	3
RBFOX1	UP	7200783	C/T	intronic	0,0,0	rs7189620	NFYB	AD	3
IST1	NA	71930399	C/T	UTR3	0,0,0	rs75182609	SRF	AD	3
ZNF614	NA	52018043	G/A	exonic	0,0,0	rs9636139	BCL3, REST,TAL1,BRCA1	AD	0
CNN3	UP	94910288	G/A	intronic	0,0,0	rs9432409	SRF	ALS	5
SLC35F3	NA	234291788	A/C	intronic	0,0,0	rs594349	ESR1,ESR2,GBX2,HOXA5,Phox2a,PRRX2,RXRA,RORA	AD	3
UVSSA	NA	1387060	A/G	UTR3	0,0,1	rs4974559	MYC,NANOG	AD	5
GRIA1	UP	153617515	C/T	intronic	0,0,0	rs17114876	SOX2	AD	4
PDE1C	UP	32000754	T/C	intronic	0,0,0	rs11766488	BCL6,HSF1,NFKB1,NFKB2,SMAD3	AD,AMD	3
FAM102A	UP	127963162	G/A	intronic	0,0,1	rs943392	STAT2	AD	6
DLG2	UP	83518543	G/A	intronic	0,0,0	rs11233657	DNMT1	AMD	4
CEP164	DOWN	117351876	G/A	exonic	0,0,0	rs490262	NR3C1,STAT	AD	0
TRIM37	DOWN	59027862	C/T	intronic	0,0,0	rs8077332	EWSR1,HOXA1	AD	4
FAM13A	UP	88754499	G/A	intronic	1,0,1	rs61231054	ZBTB7A	AD	6
ILK	DOWN	6606823	G/A	intronic	0,0,1	rs2255405	REST,YY1	AD,ALS	5
CNTN5	DOWN	99058829	A/C	intronic	0,0,0	rs11218250	GATA6,HOXA5,POU6F1,GATA2	AD	4
TPP2	DOWN	102659411	T/C	intronic	0,0,0	rs595607	Foxl1,BCL11	ALS	3
RBFOX1	UP	7197233	G/C	intronic	0,0,0	rs9928904	NA	AD	3
RBFOX1	UP	7657917	T/C	intronic	0,0,0	rs7190927	CDK2	AD,PD	4
CDC42BPA	UP	227067342	T/C	intronic	0,0,0	rs16846919	NA	AD	3
MTR	NA	236887758	A/G	intronic	0,0,0	rs3768149	NR3C1	AD	4
CBLB	DOWN	106674163	A/G	intergenic	0,0,0	rs7641939	SMAD1	AD	3
RAB33B	UP	139408936	A/G	intergenic	0,0,0	rs7687436	DNMT1	AD	3
ATXN7L1	DOWN	105744460	C/T	intronic	0,0,0	rs12667632	REST,SP1	AD	4
FAM120A	DOWN	93521022	C/T	intronic	0,0,0	rs10821148	NR3C1,NFYB	AD	3
SNX29	NA	12146941	G/C	intronic	1,0,0	rs350226	SMAD1,BCL11A	AD	3

ZNF302	NA	34678008	G/C	UTR5	0,0,0	rs67231489	NA	ALS	7
SYN3	NA	33057436	A/G	intronic	1,0,0	rs5754399	SOX4	AD	6
TMEM108	NA	133262557	A/G	intronic	0,0,0	rs766952	STAT6,ZBTB7A	AD,ALS	5
LOC10193	NA	173165105	C/G	ncRNA_exonic	1,0,0	rs934358	STAT5A	AD	3
KCNV2	NA	2770887	T/C	intergenic	0,0,0	rs4520217	STAT5A	AD,PD	4
NONE;SLIT	NA	83676475	T/C	intergenic	0,0,0	rs7987156	Gfi1b,MAF	PD	5
DENND4A	UP	65772083	T/C	intronic	0,0,0	rs17816071	JUN	AD	6
SHISA9	NA	12949419	G/T	intronic	0,0,0	rs34939433	EZH2	AD	3
EFCAB13	NA	47432130	G/A	intronic	0,0,0	rs77454872	JUN,HEY1,LMO2,TCF12,ZEB1,DNMT1	AD	3
SNX7	NA	98739972	G/C	intronic	0,0,0	rs12402587	NA	AMD	5
LINC02024	NA	117804104	C/T	intergenic	0,0,0	rs12489939	NA	AD	3
TMEM108	NA	133261101	G/A	intronic	0,0,0	rs11718689	NA	ALS	5
NDUFC1	DOWN	139300880	C/G	UTR5	1,0,0	rs3806767	TAF1	AD	5
PTPRD	UP	10371281	G/A	intronic	0,0,0	rs1535658	CDK2	AD	3
CELF2	UP	10532531	T/C	intronic	0,0,0	rs10905734	DIDO1	AD	4
SOX6	NA	16390596	C/T	intronic	0,0,0	rs61883215	JUN	AD	3
CWF19L2	NA	107386439	T/C	intronic	0,0,0	rs660962	CUX1	AD	3
GATC	NA	120462471	C/T	UTR3	0,0,1	rs9040	NA	AD	4
PHC2	NA	33324069	G/A	UTR3	1,0,0	rs11061	ZBTB1	AD	9
PHACTR3	NA	59692443	G/C	intronic	0,0,0	rs6123926	NA	AD	5
APP	UP	25885348	C/A	intronic	0,0,0	rs373521	SP1	AD,PD	5
AAK1	UP	69539897	C/T	intronic	0,0,0	rs17036759	SOX2	AD,AMD	4
MXD1	NA	69926304	T/G	intronic	0,0,1	rs10496174	GATA3,RAD21,GATA2	AD	4
LSAMP	NA	116528279	C/A	intergenic	0,0,0	rs10934350	CTBP1	AD	3
ANKH	UP	14768161	G/A	intronic	0,0,0	rs6889788	CUX1	AD	6
AGPAT4	UP	161137004	A/G	intronic	1,0,0	rs7768457	SRF	AD	3
ZBTB16	UP	114215695	G/A	intronic	0,0,0	rs495248	SRF	AD	4
SLITRK1	NA	83710374	G/A	intergenic	0,0,0	rs9546490	IRF5	ALS	4
GABRB3	NA	26703938	C/A	intronic	0,0,0	rs12905013	NF1	AD	4
RBFOX1	UP	7205157	A/G	intronic	0,0,0	rs10153112	FOXA2,BRCA	AD	3
BAIAP2	UP	81055927	G/C	intronic	0,0,0	rs111815788	Klf4,SP1,KLF5,CTBP1	AD	5
GREB1	UP	11585821	T/C	exonic	0,0,1	rs35188552	JUN,GATA6,PAX5,ZEB1	AD	0
LINC02043	NA	186812695	T/C	ncRNA_intronic	0,0,0	rs266717	ZBTB1	AD	3
SNAP91	UP	83683914	C/T	intronic	0,0,0	rs35594074	RREB1,BCL11A	AD	4
HBS1L	UP	135048076	G/A	intronic	0,1,1	rs6915770	GFI1b,HEY1	AD	4
UNC5D	NA	35326843	T/C	intronic	0,0,0	rs12680114	NA	AD	3
RPS20	DOWN	56073972	C/G	intronic	1,0,1	rs2976045	NF1	AD	5
LAMA5	DOWN	62320641	C/T	exonic	0,0,0	rs2297587	MAFg,YY1,	AD	0

STK39	UP	167983805	A/G	intronic	0,0,0	rs10497332	ESR1,ESR2,RXRA,RORA	AD	3
ZYX	DOWN	143384849	G/C	intronic	1,0,0	rs11772895	IRF5,SP1	AD	7
NAP1L1	NA	76083176	A/G	intronic	0,0,0	rs1368578	NA	AD	5
RBM26	UP	79318587	T/C	downstream	0,0,0	rs3818563	ZEB1	AMD	4
RBFOX1	UP	7197296	A/C	intronic	0,0,0	rs58998740	JMJD6	AD	3
CDYL2	NA	80736226	T/A	intronic	0,0,0	rs4497705	NA	AD,ALS	4
PIGN	NA	62067535	T/C	intronic	0,0,0	rs12458350	IRF5	AD,AMD	3
SLC45A3	NA	205706112	C/T	intergenic	0,1,0	rs72750964	IRF7,MYC	AD	3
LINC02145	NA	6181691	A/G	intergenic	0,0,0	rs2964716	RAD21,TAF1	AD	3
ARID1B	NA	156862918	A/T	intronic	1,0,0	rs12212399	MEF2A	AD	3
NTRK2	UP	84772992	A/G	intronic	0,0,0	rs7038866	MEF2A	AD	4
FTX	NA	74076916	G/A	ncRNA_intronic	0,0,0	rs5981580	HDAC1	Novel	3
FTX	NA	74081102	A/C	ncRNA_intronic	0,0,0	rs5981581	NR3C1,MZF1,SP1,E2F3,PAX5,SETDB1,WT1,NCOR1	Novel	3
LRRN2	UP	204669100	T/C	intronic	0,0,0	rs189852394	PITX2	Novel	3
ZNF789	NA	99477674	A/G	intronic	0,0,0	rs560260561	DNMT1	Novel	8
FTX	NA	74271828	T/C	ncRNA_intronic	0,0,0	rs1151602	BRF1	Novel	3
FTX	NA	74222251	T/A	ncRNA_intronic	0,0,0	rs6647462	CUX1	Novel	3
MAN1B1	DOWN	137103590	C/T	intronic	1,0,1	rs28373932	RREB1,ZBTB1	Novel	10
KIF9-AS1	NA	47178377	A/G	ncRNA_intronic	0,0,0	.	NA	Novel	0
FTX	NA	74104967	T/C	ncRNA_intronic	0,0,0	rs6647448	REST,RAD21,DIDO1	Novel	1
LOC22072	NA	197641116	T/C	intergenic	0,0,0	rs11713761	NR3C1	Novel	1
DMD	UP	31241553	C/T	intronic	0,0,0	rs5927712	GATA3	Novel	2
LOC38990	NA	3818448	C/T	ncRNA_exonic	0,0,0	rs1133336	SRF	Novel	0
TSPAN7	UP	38625725	T/A	intronic	0,0,0	rs963481	IRF5	Novel	3
RPL7L1	NA	42889285	A/G	UTR3	0,0,0	rs1134326	MEF2A	Novel	4
SIK3	UP	117027437	C/T	intronic	0,0,0	rs4938324	NA	Novel	1
UBE2H	NA	129896680	T/C	intronic	0,0,0	rs532960055	NA	Novel	8
REPS2	NA	17062966	A/T	intronic	0,0,0	rs1961490	NR3C1,TAF1	Novel	2
LOC22072	NA	197641114	C/T	intergenic	0,0,0	rs11708910	NR3C1,PAX5	Novel	1
FRMPD4	NA	12157900	A/G	intronic	0,0,0	rs5935248	NA	Novel	2
ACTRT1;SM	NA	129233821	T/G	intergenic	0,0,0	rs5932596	GLI1,RXRA	Novel	1
ARMCX3	UP	101626444	A/G	UTR3	0,0,0	rs6995	TRIM28	Novel	8

AD- Alzhemier disease, AMD-Age related macular degeneration, ALS-Amyo lateral sclerosis,
PD -Parkinson disease,
Prediction variant effect: 0->No effect 1-> functional effect, NA- Not Available,
score: include (CADD, FATHMM, Deepsea, GWAVA, Region score, TSS score, eQTL, GWAS, HGMD probability, Funseq2, REMM)

Supplementary Table S4: The number of predicted variants altering gene expression, methylation, histone acetylation and TF binding

**Category**	**Number of predicted variants**
Expression and TF binding	24
Expression	6
Histone acetylation and expression	2
Histone acetylation and TF binding	1
Histone acetylation, expression and TF binding	5
Methylation	4
Methylation and expression	1
Histone acetylation	1
Methylation and TF binding	25
Methylation, expression and TF binding	15
Methylation, Histone acetylation and TF binding	1
Methylation,Histone acetylation,expression, TF binding	1
TF binding	186

**Supplementary Table S5:** Differential gene expression study- Salmon							
**Gene name**	**Fold change in gene expression**	**C1_HG38**	**C3_HG38**	**C4_HG38**	**AD1_HG38**	**AD3_HG38**	**AD4_HG38**
CASP9	2.6	0.0	0.0	0.0	11.5	90.7	47.1
SCMH1	-3.9	492.3	316.1	32.8	0.0	0.0	0.0
CDKN2C	2.8	0.0	0.0	3.9	25.7	108.5	83.0
LEPR	-2.3	212.7	13.6	26.1	0.0	0.0	0.0
USP33	-3.0	25.4	158.6	287.6	0.0	3.6	0.0
WDR63	-2.4	341.1	199.3	190.1	42.5	35.6	41.5
HAO2	2.8	0.0	0.0	0.0	28.3	21.3	25.5
NDUFS2	3.2	0.0	0.0	0.0	49.6	81.8	22.6
GPR161	3.0	0.0	0.0	0.0	40.7	22.2	34.9
CD46	2.7	0.0	0.0	0.0	84.1	144.1	10.4
INTS7	4.8	0.0	0.0	0.0	709.4	97.8	250.8
ESRRG	2.9	0.0	0.0	0.0	21.3	30.2	37.7
JMJD4	-3.9	33.4	107.5	72.4	0.0	0.0	0.0
MRPL55	-3.0	48.2	39.6	17.4	0.0	0.0	0.0
PHYH	2.7	0.0	0.0	0.0	8.9	133.4	113.1
MKX	2.7	0.0	0.0	0.0	15.9	31.1	83.0
CREM	2.3	0.0	0.0	0.0	39.0	8.0	44.3
ZFAND4	-2.8	29.4	67.8	22.2	0.0	0.0	1.9
SFTPA1	-2.5	17.4	24.0	16.4	0.0	0.0	0.0
SFXN3	-2.6	17.4	694.8	1246.9	28.3	17.8	34.9
MTG1	-3.9	33.4	65.7	77.2	0.0	0.0	0.0
CYP2E1	-3.9	49.5	112.7	41.5	0.0	0.0	0.0
TSSC4	3.5	0.0	3.1	0.0	207.2	201.9	34.9
USH1C	-2.9	951.1	381.8	312.7	26.6	75.6	17.0
MS4A4A	-2.4	168.5	162.7	24.1	15.1	8.9	2.8
NPAS4	-2.3	121.7	79.3	480.6	22.1	24.9	33.0
ALG8	3.1	0.0	0.0	0.0	35.4	28.5	31.1
HTR3B	2.7	0.0	0.0	1.9	21.3	27.6	69.8
CADM1	-6.5	258.2	248.3	443.9	0.0	0.0	0.0
CACNA1C	4.1	0.0	0.0	0.0	46.1	143.2	296.0
CHD4	4.9	0.0	0.0	0.0	364.0	86.3	186.7
ATF7IP	2.4	0.0	0.0	0.0	33.7	22.2	12.3
PAN2	-4.9	69.6	364.1	235.5	0.0	0.0	0.0
SLC25A3	-5.5	208.7	203.4	101.3	0.0	0.0	0.0
CCDC62	2.4	0.0	0.0	0.0	24.8	34.7	11.3
P2RX2	-3.0	25.4	68.9	24.1	0.0	0.0	0.0
POSTN	-3.1	78.9	19.8	250.0	0.0	0.0	0.0
SLC7A7	2.2	0.0	0.0	0.0	37.2	16.9	12.3
RBM23	4.8	0.0	0.0	0.0	645.6	740.0	79.2
KHNYN	-2.1	302.3	160.7	165.0	58.5	26.7	25.5
STXBP6	2.5	0.0	0.0	0.0	30.1	32.0	13.2

WARS	-4.8	169.9	418.3	79.1	0.0	0.0	0.0
SNORD114	-3.2	2084.1	883.6	670.7	21.3	9.8	107.5
ZNF839	4.0	0.0	0.0	0.0	70.0	179.7	50.0
CHRM5	-2.1	1145.0	285.9	352.2	123.1	111.2	72.6
RAB27A	-5.6	167.2	136.7	139.9	0.0	0.0	0.9
RAB27A	5.0	0.0	0.0	0.0	92.1	117.4	198.0
RORA	3.2	0.0	0.0	0.0	37.2	33.8	30.2
MEGF11	2.3	0.0	0.0	0.0	49.6	8.9	33.9
MFGE8	-2.9	56.2	126.2	13.5	0.0	0.0	0.0
RGMA	-2.7	9.4	82.4	188.2	0.0	0.0	0.0
RGS11	2.3	0.0	0.0	0.0	19.5	11.6	38.7
ZC3H7A	-5.8	131.1	396.4	456.5	0.0	0.0	0.0
DUS2	-3.1	25.4	68.9	26.1	0.9	0.0	0.0
DEF8	-3.9	777.2	344.3	40.5	0.0	0.0	0.0
AIPL1	-2.5	9.4	41.7	49.2	0.0	0.0	0.0
NCOR1	-4.5	88.3	175.3	692.9	0.0	0.0	0.0
PRPSAP2	-3.1	117.7	15.6	67.6	0.0	0.0	0.0
MFAP4	3.2	0.0	0.0	0.0	20.4	133.4	62.2
RHOT1	3.0	0.0	0.0	0.0	31.0	24.9	32.1
PSMC3IP	3.1	1.3	0.0	0.0	57.6	69.4	17.9
C17orf53	3.6	0.0	0.0	0.0	34.5	91.6	45.2
UBTF	-3.6	70.9	77.2	26.1	0.0	0.0	0.0
GJC1	2.7	0.0	1.0	0.0	90.3	8.9	95.2
MAP3K3	-3.3	42.8	48.0	23.2	0.0	0.0	0.0
HN1	-6.7	255.5	412.1	362.9	0.0	0.0	0.0
MXRA7	-4.9	543.1	485.1	194.9	5.3	0.0	0.0
MTCL1	-4.4	46.8	243.1	175.6	0.0	0.0	0.0
RBBP8	4.0	0.0	0.0	0.0	39.0	71.2	85.8
PSTPIP2	5.4	0.0	0.0	0.0	124.9	123.6	147.1
MRO	2.5	4.0	121.0	21.2	1502.9	284.6	1194.4
WDR18	-6.1	194.0	183.6	336.8	0.0	0.0	0.0
TCF3	3.3	0.0	0.0	0.0	34.5	161.9	38.7
ZNF627	-2.4	814.6	610.3	683.3	80.6	72.9	175.3
DPF1	-4.0	129.8	235.8	34.7	0.0	0.0	0.0
PRX	2.5	0.0	0.0	0.0	10.6	132.5	42.4
POLD1	-2.3	4.0	230.6	162.1	0.0	0.0	0.0
GREB1	5.6	0.0	0.0	0.0	203.7	212.6	662.7
STON1	5.3	0.0	0.0	0.0	104.5	137.0	148.9
PRKRA	3.4	0.0	0.0	0.0	116.0	20.5	146.1
SLC39A10	5.2	0.0	0.0	0.0	263.0	116.5	134.8
SATB2-AS1	2.2	12.0	7.3	11.6	47.8	49.8	129.1
ANKMY1	-2.5	37.5	36.5	10.6	0.0	0.0	0.0
SNED1	-5.3	103.0	135.6	105.2	0.0	0.0	0.0
TNNC2	-2.7	68.2	39.6	12.5	0.0	0.0	0.0
SLCO4A1	4.0	0.0	0.0	0.0	92.1	60.5	43.4
DIDO1	6.3	0.0	0.0	0.0	611.1	924.1	239.4
IFNAR2	-2.4	234.1	257.7	130.3	24.8	5.3	0.0

CLIC6	-2.6	390.6	583.2	306.9	9.7	37.4	0.0
AC005301.	-2.4	14.7	24.0	18.3	0.0	0.0	0.0
TSSK2	-2.2	25.4	11.5	16.4	0.0	0.0	0.0
CABP7	-2.1	4770.0	10210.3	5783.6	1305.4	1893.5	341.3
PES1	2.7	0.0	0.0	0.0	13.3	65.8	36.8
PISD	2.4	0.0	0.0	0.0	23.9	20.5	16.0
CYP2D6	3.1	0.0	0.0	0.0	56.7	26.7	27.3
SEC13	-3.2	235.4	59.5	25.1	0.0	0.0	0.0
PDCD6IP	4.0	0.0	0.0	0.0	38.1	393.1	269.6
XIRP1	2.5	0.0	0.0	0.0	26.6	24.9	14.1
CCR9	-2.6	60.2	19.8	15.4	0.9	0.0	0.0
NDUFAF3	-2.8	73.6	57.4	11.6	0.0	0.0	0.0
LAMB2	-4.5	77.6	171.1	64.7	0.0	0.0	0.0
ALAS1	-2.5	129.8	8.3	55.0	0.0	0.0	0.0
GNL3	-2.3	1409.9	105.4	222.9	73.5	36.5	34.9
PDHB	-5.2	355.8	138.8	111.0	0.0	0.0	0.0
SYNPR	-7.1	351.8	1127.8	887.9	0.0	0.0	0.0
ST3GAL6	-2.9	36.1	109.5	14.5	0.0	0.9	0.0
B3GALNT1	2.8	78.9	44.9	25.1	761.6	273.0	482.7
FRAS1	-2.7	64.2	76.2	63.7	5.3	0.0	0.0
SPRY1	3.6	0.0	0.0	0.0	44.3	36.5	60.3
RHOBTB3	2.7	21.4	12.5	5.8	116.9	241.9	87.7
NR3C1	2.9	0.0	0.0	0.0	46.9	24.9	24.5
RMND5B	-4.6	224.7	66.8	95.5	0.0	0.0	0.0
IER3	2.4	0.0	0.0	0.0	36.3	14.2	17.9
AGPAT1	4.8	0.0	0.0	0.0	108.9	119.2	73.5
AGPAT1	4.0	0.0	0.0	0.0	285.2	141.4	39.6
TPBG	2.9	0.0	3.1	0.0	28.3	69.4	229.1
CEP162	4.3	0.0	0.0	0.0	75.3	97.8	333.7
SYNE1	-2.1	109.7	121.0	66.6	20.4	18.7	16.0
PARK2	3.4	5.4	0.0	0.0	94.8	73.8	130.1
KDELR2	2.8	1.3	0.0	0.0	22.1	72.9	22.6
HDAC9	-6.2	983.2	459.0	227.8	0.0	0.0	0.0
AC003986.	2.4	0.0	1.0	0.0	15.1	16.0	55.6
GPNMB	2.1	343.8	118.9	432.3	1075.1	2009.2	1920.3
TAC1	-2.2	248.8	187.8	112.9	34.5	16.9	41.5
TAF6	3.6	20.1	0.0	12.5	282.5	1029.9	505.3
HBP1	-4.6	191.3	116.8	55.0	0.0	0.0	0.0
PNPLA8	3.3	0.0	0.0	0.0	106.3	106.7	19.8
NRF1	2.6	0.0	0.0	0.0	23.0	16.9	26.4
MEST	7.4	0.0	0.0	0.0	643.8	489.2	689.1
C8orf58	4.3	0.0	0.0	0.0	69.1	53.4	83.9
TSTA3	-3.6	26.8	55.3	67.6	0.0	0.0	0.0
CNTFR	2.5	0.0	29.2	13.5	272.8	318.4	157.4
FOXD4L6	-2.9	10.7	133.5	155.4	0.0	0.0	0.0
TLE4	-2.2	2232.5	318.2	563.6	87.7	225.9	88.6
PHYHD1	2.6	0.0	0.0	0.0	23.9	16.9	25.5

PTGES	-3.8	30.8	192.0	105.2	0.0	0.0	0.0
CARD9	-2.9	17.4	36.5	108.1	0.0	0.0	0.0
DCX	3.8	0.0	0.0	0.0	232.9	30.2	252.6
FGF13	-4.7	333.1	55.3	278.9	0.0	0.0	0.0
ARHGAP4	-3.9	191.3	151.3	29.0	0.0	0.0	0.0
TTTY15	-2.3	68.2	1004.7	762.4	23.0	52.5	94.3
DDX3Y	-2.7	61.5	1333.3	1371.4	64.6	18.7	61.3
PHTF1	2.3	0.0	0.0	16.4	271.9	292.6	120.7
HPSE2	2.3	0.0	0.0	0.0	14.2	17.8	22.6
ALKBH2	2.2	0.0	0.0	0.0	26.6	48.0	8.5
ALOXE3	3.5	0.0	0.0	0.0	42.5	86.3	34.9
SEZ6	-6.2	171.2	515.4	514.4	0.0	0.0	0.0
DTNA	5.3	0.0	0.0	0.0	500.4	492.7	100.9
ZNF44	-3.0	28.1	27.1	27.0	0.0	0.0	0.0
SFXN5	-3.8	70.9	36.5	44.4	0.0	0.0	0.0
ST3GAL6	-2.3	321.0	355.7	105.2	14.2	40.0	45.2
RXFP1	3.5	0.0	0.0	0.0	26.6	92.5	60.3
SH3TC2	-2.6	181.9	35.5	15.4	0.0	0.0	0.0
FNDC1	-2.4	287.6	699.0	366.7	60.2	87.2	63.2
MTERF3	-2.9	29.4	33.4	19.3	0.0	0.0	0.0
SPATA31A3	2.5	0.0	0.0	0.0	23.9	124.5	16.0
VPS45	3.1	0.0	0.0	0.0	55.8	66.7	18.9
SLC39A1	-2.6	1094.2	514.3	319.4	3.5	64.0	55.6
SYT14	-4.0	608.6	158.6	51.1	0.9	0.0	0.0
TSNAX-DISC	-2.1	400.0	357.8	240.3	69.1	91.6	44.3
RRP12	-4.3	73.6	79.3	46.3	0.0	0.0	0.0
EPS8L2	-3.1	52.2	19.8	35.7	0.0	0.9	0.0
PGAP2	-3.3	30.8	31.3	37.6	0.0	0.0	0.0
C2CD5	-2.5	66.9	8.3	60.8	0.0	0.0	0.0
LMO7	-3.7	45.5	31.3	67.6	0.0	0.0	0.0
NARFL	2.3	0.0	0.0	0.0	27.5	50.7	9.4
TMC5	-3.6	34.8	38.6	58.9	0.0	0.0	0.0
ACADVL	2.7	0.0	0.0	0.0	42.5	13.3	56.6
C18orf42	-2.1	152.5	175.3	85.9	6.2	39.1	12.3
BFSP1	2.3	0.0	0.0	0.0	16.8	40.0	13.2
TMEM189	-4.1	175.2	123.1	36.7	0.0	0.0	0.0
DIP2A	5.2	0.0	3.1	0.0	161.2	949.0	1212.3
TSEN2	2.7	0.0	0.0	0.0	24.8	22.2	114.1
IP6K2	-3.1	29.4	28.2	29.9	0.0	0.0	0.0
EIF2A	3.1	0.0	0.0	0.0	30.1	24.0	48.1
TNIP1	-3.1	61.5	51.1	17.4	0.0	0.0	0.0
OPRM1	2.5	0.0	0.0	0.0	24.8	14.2	36.8
DDX39B	5.5	0.0	0.0	0.0	158.5	777.3	318.6
VARS	-3.1	18.7	36.5	51.1	0.0	0.0	0.0
OR2H2	-4.6	73.6	98.1	262.5	0.0	0.9	0.0
DDX39B	2.3	0.0	0.0	1.0	134.6	29.4	7.5
TCF19	-2.2	10.7	16.7	33.8	0.0	0.0	0.0

TCF19	-2.2	10.7	16.7	33.8	0.0	0.0	0.0
VARS	-3.1	18.7	36.5	51.1	0.0	0.0	0.0
TUBB	-2.5	500.3	5.2	174.7	0.9	2.7	0.0
DAQB-335/	2.4	2.7	10.4	4.8	63.8	59.6	45.2
RPP21	2.3	0.0	0.0	0.0	27.5	9.8	98.0
BRD2	4.0	0.0	0.0	0.0	55.8	50.7	56.6
HDAC9	-2.7	634.0	403.7	303.0	24.8	0.0	29.2
SPATA6L	2.8	0.0	0.0	0.0	15.1	42.7	43.4
FIBCD1	-2.4	224.7	1404.2	719.9	141.7	64.0	57.5
ABCA2	-5.3	103.0	108.5	175.6	0.0	0.0	0.0
FAM223A	2.4	0.0	0.0	0.0	16.8	28.5	16.0
KDM5D	-3.0	18.7	615.5	503.8	5.3	13.3	8.5
NBL1	-5.0	177.9	78.2	142.8	0.9	0.0	0.0
PDZD7	-2.6	20.1	50.1	16.4	0.0	0.0	0.0
GNGT2	-2.3	48.2	18.8	24.1	2.7	2.7	2.8
DPF1	-3.1	32.1	43.8	21.2	0.0	0.0	0.0
TGOLN2	2.4	13.4	0.0	0.0	88.6	332.6	352.6
ATXN7	7.4	0.0	0.0	0.0	489.7	1150.9	1121.8
TIPARP	5.4	0.0	0.0	0.0	166.5	110.3	190.4
MAGI2-AS3	2.7	0.0	0.0	0.0	46.1	40.9	13.2
SNORA11	3.1	0.0	0.0	0.0	73.5	17.8	62.2
AKR1E2	2.6	0.0	0.0	0.0	21.3	48.0	17.9
CRIP2	2.5	0.0	0.0	0.0	143.5	8.0	77.3
SPRY1	-4.6	100.3	55.3	110.0	0.0	0.0	0.0
WDR74	2.3	49.5	52.2	54.0	393.2	257.9	216.8
NAP1L4	-3.5	64.2	499.7	257.7	0.0	0.0	4.7
U2	2.8	606.0	3099.5	5010.6	15445.0	32330.8	71883.6
ACACA	-2.3	671.5	569.6	5.8	3.5	8.0	0.0
SDCCAG8	-2.1	1680.1	686.5	449.7	45.2	104.1	254.5
CBSL	6.3	0.0	0.0	0.0	821.0	324.6	301.7
PCAT14	2.1	36.1	18.8	1.9	94.8	209.9	192.3
SEC14L2	2.7	0.0	0.0	0.0	204.6	21.3	35.8
WDR86	-2.7	88.3	126.2	182.4	8.0	0.0	0.0
TSTA3	-3.6	26.8	55.3	67.6	0.0	0.0	0.0
FAM223B	-2.3	28.1	9.4	24.1	0.0	0.0	0.0
KLHL17	-2.4	53.5	43.8	39.6	3.5	0.0	5.7
RPL22	-5.3	1862.0	661.4	178.5	0.0	0.0	0.0
SLC2A5	-3.2	124.4	65.7	17.4	0.0	0.0	0.0
PADI2	3.5	0.0	0.0	0.0	113.4	23.1	94.3
MINOS1	-2.7	64.2	25.0	16.4	0.0	0.0	0.0
NBL1	3.2	0.0	0.0	0.0	31.0	82.7	31.1
VWA5B1	-2.5	25.4	13.6	24.1	0.0	0.0	0.0
SYTL1	-3.0	20.1	31.3	37.6	0.0	0.0	0.0
EYA3	3.6	0.0	0.0	0.0	146.1	110.3	24.5
SVBP	3.5	0.0	0.0	0.0	509.2	37.4	123.5
ATP6V0B	2.2	0.0	0.0	0.0	43.4	9.8	20.7
CCDC24	-2.2	45.5	11.5	14.5	0.0	0.0	0.0

TTC39A	-3.1	29.4	90.8	33.8	1.8	0.0	0.0
NRD1	-4.4	45.5	130.4	166.0	0.0	0.0	0.0
DAB1-AS1	-2.3	86.9	44.9	12.5	1.8	0.0	3.8
IL12RB2	-2.0	763.8	732.4	531.7	155.9	190.3	94.3
SRSF11	2.7	0.0	0.0	0.0	15.1	33.8	99.0
LMO4	2.5	65.5	83.5	33.8	85.9	758.7	1598.8
SNX7	2.6	0.0	0.0	0.0	37.2	12.5	56.6
SORT1	-2.3	13.4	19.8	18.3	0.0	0.0	0.0
PHTF1	-4.3	620.7	277.5	289.5	7.1	0.0	0.0
S100A6	-3.3	42.8	35.5	26.1	0.0	0.0	0.0
SLC50A1	3.1	0.0	0.0	0.0	14.2	150.3	147.1
SEMA4A	-4.1	52.2	65.7	48.3	0.0	0.0	0.0
PEX19	-2.7	444.1	241.0	472.9	39.9	53.4	50.9
ILDR2	-2.2	359.8	75.1	97.5	0.0	16.9	10.4
RC3H1	3.0	0.0	3.1	0.0	51.4	32.0	122.6
TNN	2.2	0.0	0.0	0.0	44.3	44.5	6.6
TNNT2	2.4	0.0	0.0	1.9	13.3	25.8	90.5
CSRP1	2.9	0.0	0.0	0.0	40.7	56.9	16.0
EIF2D	2.8	0.0	0.0	0.0	29.2	65.8	17.9
EIF2D	-2.7	13.4	48.0	33.8	0.0	0.0	0.0
MRPL55	3.0	0.0	0.0	0.0	55.8	22.2	32.1
PCNXL2	-2.6	22.7	78.2	57.9	3.5	0.0	0.0
GNG4	2.6	0.0	0.0	0.0	52.3	9.8	77.3
PGBD2	3.4	0.0	0.0	0.0	35.4	32.0	43.4
AKR1E2	-2.6	33.4	13.6	24.1	0.0	0.0	0.0
RBM17	-2.5	45.5	214.9	12.5	0.0	0.0	0.0
OPTN	2.3	204.7	85.5	32.8	591.6	1114.4	714.6
SIRT1	3.6	0.0	0.0	0.0	28.3	88.9	83.0
NUTM2D	3.7	0.0	0.0	0.0	46.9	50.7	176.3
CASP7	3.4	0.0	0.0	0.0	23.9	81.8	69.8
ABLIM1	2.8	0.0	0.0	1.9	15.9	48.9	69.8
ADAM8	2.2	0.0	0.0	2.9	23.9	32.9	19.8
IRF7	2.8	0.0	0.0	0.0	47.8	21.3	21.7
RPLP2	-2.6	20.1	20.9	20.3	0.0	0.0	0.0
AC091053.	2.4	0.0	12.5	8.7	22.1	197.4	591.1
CAPRIN1	-2.2	1207.9	292.1	139.9	103.6	23.1	17.0
CPSF7	2.5	0.0	0.0	1.9	15.9	25.8	91.4
DAGLA	-2.3	5.4	179.4	72.4	0.0	0.0	0.0
UBXN1	-2.2	141.8	44.9	48.3	6.2	4.4	16.0
RASGRP2	-2.2	46.8	18.8	44.4	0.0	3.6	0.0
MEN1	2.8	0.0	0.0	0.0	11.5	73.8	121.6
ATG2A	-3.0	105.7	23.0	30.9	0.0	0.0	0.0
VPS51	2.8	0.0	0.0	0.0	138.2	40.0	17.0
MRPL49	3.2	0.0	0.0	0.0	37.2	40.0	25.5
MRPL49	2.2	0.0	9.4	4.8	96.5	24.0	99.0
RNASEH2C	-2.7	41.5	16.7	22.2	0.0	0.0	0.0
FIBP	2.8	0.0	0.0	0.0	52.3	40.9	14.1

BRMS1	3.1	0.0	0.0	0.0	24.8	47.1	31.1
DPP3	4.0	0.0	0.0	0.0	56.7	73.8	45.2
BBS1	-4.6	82.9	78.2	57.9	0.0	0.0	0.0
CTSF	2.7	60.2	51.1	8.7	782.9	634.1	181.9
RBM4	-2.0	234.1	96.0	291.4	10.6	56.9	26.4
GSTP1	4.3	0.0	0.0	0.0	43.4	171.7	181.0
ANAPC15	2.6	0.0	0.0	0.0	18.6	22.2	26.4
PDE2A	2.6	0.0	0.0	0.0	9.7	72.9	212.1
CEP57	2.6	0.0	0.0	0.0	59.3	244.6	13.2
CASP4	3.1	0.0	0.0	0.0	27.5	26.7	52.8
PPP2R1B	3.0	0.0	0.0	0.0	19.5	47.1	43.4
TMEM25	-2.9	89.6	77.2	11.6	0.0	0.0	0.0
PHLDB1	2.4	0.0	0.0	0.0	11.5	40.9	27.3
CCDC84	2.8	0.0	2.1	0.0	32.8	23.1	44.3
HSPA8	3.1	49.5	17.7	96.5	2875.6	1601.8	165.9
TBRG1	-6.0	140.5	273.3	256.7	0.0	0.0	0.0
NTM	2.5	0.0	0.0	0.0	46.1	14.2	21.7
GLB1L3	2.5	0.0	0.0	0.0	8.9	51.6	105.6
RAD52	-3.5	53.5	29.2	40.5	0.0	0.0	0.0
DCP1B	4.2	0.0	0.0	0.0	105.4	51.6	65.0
M6PR	-2.4	279.6	271.2	30.9	0.0	9.8	10.4
A2M	2.9	0.0	0.0	0.0	54.0	20.5	28.3
NELL2	-2.2	24.1	27.1	19.3	2.7	1.8	2.8
ARID2	3.0	0.0	0.0	0.0	52.3	21.3	33.0
DDX23	3.9	0.0	0.0	0.0	63.8	55.1	231.0
SMUG1	-3.2	26.8	27.1	56.0	0.0	0.0	0.0
TESPA1	2.3	45.5	15.6	74.3	233.8	396.7	368.6
RP11-977G	-2.8	21.4	123.1	63.7	0.0	0.0	2.8
SHMT2	-3.2	132.4	85.5	16.4	0.0	0.0	0.0
OS9	2.4	0.0	0.0	0.0	166.5	5.3	194.2
SLC16A7	-2.8	314.3	54.2	49.2	6.2	8.0	6.6
HELB	-3.8	112.4	64.7	31.8	0.0	0.0	0.0
MDM1	3.1	4.0	0.0	0.0	59.3	92.5	45.2
MDM2	-5.3	192.6	518.5	265.4	0.0	3.6	0.0
OSBPL8	2.5	0.0	0.0	0.0	12.4	88.1	29.2
RP11-90C1	-2.2	53.5	53.2	19.3	0.0	4.4	3.8
METTL25	-2.8	54.8	1164.3	1027.8	8.0	51.6	30.2
NR2C1	2.2	0.0	0.0	0.0	27.5	10.7	21.7
TMPO	3.1	0.0	3.1	0.0	131.1	61.4	34.9
ACTR6	-3.2	1547.7	214.9	182.4	24.8	1.8	12.3
MYBPC1	2.6	0.0	0.0	1.9	43.4	16.0	33.0
TCP11L2	3.7	0.0	0.0	0.0	140.8	29.4	102.8
CRY1	2.1	0.0	3.1	1.9	20.4	28.5	19.8
ISCU	4.7	0.0	0.0	0.0	110.7	192.1	74.5
GPN3	2.6	0.0	0.0	2.9	47.8	24.9	32.1
TAOK3	5.0	0.0	0.0	0.0	120.4	86.3	139.5
CAMKK2	2.5	0.0	0.0	4.8	675.7	2019.0	3681.2

RP11-463O	2.3	2.7	1.0	1.0	16.8	33.8	21.7
CDK2AP1	2.8	0.0	0.0	0.0	46.1	40.9	15.1
POLE	3.8	0.0	6.3	0.0	248.0	618.1	121.6
STARD13-A	2.8	0.0	3.1	3.9	19.5	101.4	109.4
POSTN	-2.2	80.3	45.9	58.9	13.3	7.1	2.8
COG6	2.7	113.7	38.6	65.6	1144.2	689.3	300.7
ENOX1	3.5	0.0	0.0	1.9	49.6	64.9	37.7
KIAA0226L	-2.4	9.4	55.3	30.9	0.0	0.0	0.0
EBPL	-2.8	21.4	75.1	56.9	0.0	0.0	2.8
DACH1	2.0	46.8	70.9	41.5	184.2	211.7	375.2
TBC1D4	2.2	0.0	0.0	0.0	9.7	20.5	53.7
AL355075.1	2.2	14.7	5.2	1.0	77.9	63.1	59.4
APEX1	-3.6	216.7	24.0	130.3	0.0	0.0	0.0
NDRG2	-2.8	251.5	259.8	45.4	6.2	0.0	0.0
GMPR2	3.9	0.0	0.0	0.0	46.1	56.9	132.9
RABGGTA	2.3	0.0	0.0	0.0	12.4	17.8	36.8
DHRS1	-2.3	14.7	14.6	23.2	0.0	0.0	0.0
PPP2R3C	2.0	14.7	12.5	4.8	75.3	59.6	52.8
EXD2	4.7	0.0	1.0	1.0	117.8	98.7	329.9
PSEN1	3.2	0.0	0.0	0.0	29.2	29.4	65.0
RPS6KL1	2.6	0.0	0.0	0.0	25.7	15.1	55.6
RP11-371E	-2.3	14.7	16.7	19.3	0.0	0.0	0.0
JAG2	3.1	0.0	0.0	0.0	23.9	35.6	41.5
BRF1	3.3	0.0	10.4	0.0	190.4	216.1	312.0
MTA1	3.4	0.0	0.0	0.0	21.3	185.0	142.3
RP11-371E	-2.3	14.7	16.7	19.3	0.0	0.0	0.0
GOLGA8T	-2.3	93.6	10.4	21.2	0.0	0.0	0.0
IVD	2.2	0.0	0.0	0.0	7.1	55.1	36.8
STRC	2.6	0.0	21.9	22.2	163.8	332.6	247.9
GLDN	-2.9	191.3	56.3	17.4	0.0	0.0	0.0
TCF12	-2.3	131.1	137.7	89.8	30.1	8.9	6.6
MEGF11	-2.6	120.4	322.4	219.1	35.4	17.8	24.5
SNAPC5	2.5	0.0	0.0	0.0	39.0	16.0	20.7
LCTL	2.9	0.0	0.0	0.0	31.9	18.7	56.6
CLK3	3.9	0.0	0.0	0.0	47.8	62.3	46.2
ULK3	-3.1	20.1	555.0	169.9	0.0	0.0	0.0
EFTUD1	2.3	0.0	0.0	0.0	8.0	42.7	33.9
MEGF11	-2.6	120.4	322.4	219.1	35.4	17.8	24.5
RHOT2	4.1	0.0	0.0	0.0	81.5	133.4	46.2
TSC2	4.9	0.0	2.1	1.0	401.2	89.8	356.3
MLST8	-4.9	123.1	205.5	70.4	0.0	0.0	0.0
ANKS3	2.4	0.0	0.0	0.0	21.3	52.5	12.3
ROGDI	-3.7	65.5	410.0	59.8	0.0	0.0	0.0
RSL1D1	-2.7	262.2	168.0	8.7	0.0	0.0	0.0
BFAR	-2.3	22.7	10.4	23.2	0.0	0.0	0.0
NDE1	2.3	4.0	9.4	16.4	32.8	109.4	145.2
SH2B1	2.4	0.0	0.0	0.0	7.1	57.8	113.1

RP11-345J4	2.5	0.0	0.0	0.0	17.7	20.5	27.3
MVP	-4.2	88.3	68.9	42.5	0.0	0.0	0.0
ALDOA	2.6	0.0	0.0	0.0	31.9	15.1	29.2
CYLD	-2.7	28.1	19.8	19.3	0.0	0.0	0.0
COQ9	-3.5	143.1	130.4	162.1	0.0	8.0	6.6
NDRG4	-4.7	85.6	64.7	115.8	0.0	0.0	0.0
SF3B3	2.6	0.0	0.0	0.0	10.6	46.2	70.7
KARS	-2.3	13.4	86.6	19.3	0.0	0.0	1.9
CNTNAP4	3.2	0.0	2.1	0.0	665.1	18.7	847.5
CMC2	3.5	0.0	0.0	0.0	72.6	31.1	42.4
CMC2	-6.3	301.0	225.3	199.8	0.0	0.0	0.0
TAF1C	2.7	0.0	0.0	0.0	29.2	17.8	32.1
TLDC1	-3.0	37.5	20.9	29.9	0.0	0.0	0.0
CHMP1A	2.6	0.0	0.0	0.0	9.7	83.6	61.3
VPS53	2.1	62.9	40.7	80.1	148.8	377.1	606.2
VPS53	-2.1	593.9	621.8	442.0	108.9	154.8	72.6
DPH1	-2.3	65.5	25.0	8.7	0.0	0.0	0.0
PLD2	3.3	0.0	0.0	0.0	21.3	86.3	183.8
C17orf49	-4.9	288.9	141.9	74.3	0.0	0.0	0.0
SLC16A11	2.4	0.0	0.0	0.0	15.1	35.6	18.9
TMEM256-	2.2	0.0	0.0	0.0	37.2	8.9	25.5
MPDU1	2.4	0.0	0.0	0.0	19.5	20.5	18.9
SAT2	3.8	2.7	0.0	0.0	70.0	57.8	162.1
SPAG5	2.2	21.4	13.6	36.7	97.4	191.2	146.1
LRRC37B	2.2	0.0	14.6	0.0	110.7	325.5	135.7
AP2B1	-3.4	72.2	24.0	48.3	0.0	0.0	0.0
AARSD1	-2.4	111.0	281.7	3.9	0.9	0.0	0.0
PLCD3	2.4	0.0	0.0	0.0	17.7	17.8	24.5
ARL17B	-2.5	57.5	8.3	97.5	0.0	0.0	0.0
CDK5RAP3	2.5	0.0	0.0	0.0	18.6	18.7	100.9
LPO	2.7	0.0	1.0	0.0	33.7	15.1	34.9
TRIM37	-5.3	295.6	117.9	124.5	0.0	0.0	0.0
CEP112	-2.3	17.4	13.6	18.3	0.0	0.0	0.0
HELZ	6.0	0.0	0.0	0.0	178.9	353.1	255.5
FAM20A	2.8	0.0	0.0	0.0	36.3	101.4	17.0
DNAI2	-2.9	25.4	24.0	25.1	0.0	0.0	0.0
GGA3	5.2	0.0	0.0	0.0	116.9	117.4	197.0
H3F3B	-3.4	61.5	25.0	43.4	0.0	0.0	0.0
JMJD6	3.9	0.0	0.0	0.0	112.5	41.8	58.4
LRRC37A	5.9	0.0	0.0	0.0	202.8	257.9	598.6
LPIN2	4.4	0.0	0.0	0.0	93.9	56.0	161.2
SEH1L	3.9	0.0	2.1	0.0	77.9	71.2	54.7
RIOK3	3.4	0.0	0.0	0.0	68.2	36.5	31.1
ZBTB7C	-2.5	44.1	49.0	8.7	0.0	0.0	0.0
DYM	2.2	0.0	0.0	0.0	33.7	15.1	13.2
FECH	-3.6	38.8	34.4	44.4	0.0	0.0	0.0
ABCA7	-2.5	379.9	161.7	113.9	19.5	22.2	34.9

MUM1	2.0	44.1	19.8	47.3	135.5	185.0	248.9
IZUMO4	2.6	0.0	0.0	0.0	32.8	61.4	12.3
AES	-2.1	161.9	581.1	334.9	38.1	85.4	6.6
CTB-50L17.	3.3	1.3	0.0	0.0	53.1	68.5	22.6
DPP9	-3.2	48.2	61.6	40.5	0.0	0.0	2.8
DPP9	3.6	0.0	0.0	0.0	27.5	72.0	71.6
KDM4B	2.4	0.0	0.0	3.9	34.5	30.2	40.5
LONP1	2.3	0.0	19.8	38.6	77.9	695.5	656.1
CAPS	-2.9	50.8	21.9	24.1	0.0	0.0	0.0
TUBB4A	-2.2	70.9	117.9	198.8	32.8	10.7	8.5
STXBP2	2.6	0.0	0.0	0.0	78.8	33.8	13.2
43161	3.6	0.0	0.0	0.0	31.9	88.9	52.8
C19orf66	-2.8	45.5	78.2	13.5	0.0	0.0	0.0
DNMT1	2.9	2.7	0.0	0.0	65.5	55.1	23.6
DOCK6	2.3	2.7	3.1	9.7	35.4	68.5	43.4
ZNF788	3.2	0.0	0.0	0.0	18.6	101.4	71.6
NFIX	2.8	0.0	0.0	0.0	58.5	12.5	96.2
PRKACA	3.0	0.0	2.1	0.0	17.7	97.8	83.9
PIK3R2	2.7	0.0	0.0	0.0	15.1	34.7	42.4
DDX49	-3.7	107.0	173.2	27.0	0.0	0.0	0.0
TMEM161A	2.4	0.0	0.0	0.0	11.5	45.4	27.3
GMIP	3.6	0.0	4.2	2.9	139.9	97.8	56.6
ZNF682	2.9	0.0	0.0	0.0	15.1	75.6	56.6
ZNF429	2.8	0.0	0.0	0.0	13.3	65.8	47.1
ZNF793	-2.2	85.6	18.8	10.6	0.0	0.0	0.0
SPINT2	-3.0	30.8	18.8	56.9	0.0	0.0	0.0
HNRNPL	2.2	50.8	123.1	53.1	395.9	470.5	426.1
SHKBP1	-2.4	56.2	20.9	46.3	1.8	4.4	0.0
RAB4B	-3.3	107.0	206.6	18.3	0.0	0.0	0.0
EXOSC5	3.3	0.0	0.0	0.0	48.7	29.4	166.9
XRCC1	-3.0	151.2	26.1	31.8	0.0	0.0	0.0
ERCC1	2.8	0.0	0.0	0.0	19.5	26.7	33.9
NPAS1	-2.2	26.8	11.5	16.4	0.0	0.0	0.0
KPTN	3.3	0.0	0.0	0.0	85.0	35.6	32.1
NAPA	-4.4	496.3	91.8	103.3	0.0	0.0	0.0
SPHK2	-2.0	264.9	147.1	177.6	30.1	56.9	28.3
FUZ	-3.0	26.8	26.1	29.0	0.0	0.0	0.0
MYH14	3.5	0.0	0.0	7.7	186.9	137.9	450.6
NR1H2	4.3	0.0	0.0	0.0	150.6	87.2	50.9
FPR3	2.6	0.0	0.0	0.0	15.9	32.0	22.6
LSM14A	3.1	0.0	0.0	0.0	185.1	17.8	395.9
KIR3DX1	3.1	0.0	0.0	0.0	16.8	87.2	79.2
SH3YL1	-2.6	95.0	53.2	9.7	0.0	0.0	0.0
MPV17	2.9	0.0	2.1	0.0	102.7	15.1	104.6
TTC7A	2.2	0.0	0.0	0.0	15.1	14.2	21.7
AC012358.	2.2	5.4	0.0	5.8	17.7	61.4	132.9
EFEMP1	4.0	0.0	0.0	0.0	40.7	127.2	77.3

UGP2	3.2	0.0	2.1	0.0	49.6	32.0	44.3
TRABD2A	2.5	0.0	0.0	0.0	21.3	14.2	37.7
MAT2A	2.3	0.0	0.0	0.0	19.5	14.2	128.2
KANSL3	-4.6	113.7	60.5	88.8	0.9	0.0	0.0
IMP4	2.3	0.0	0.0	0.0	16.8	13.3	28.3
GAD1	2.7	0.0	1.0	0.0	8.9	65.8	107.5
ATF2	3.5	0.0	0.0	0.0	94.8	33.8	45.2
SATB2	3.3	0.0	0.0	0.0	36.3	32.9	34.9
TTLL4	2.7	0.0	0.0	0.0	39.9	30.2	15.1
SPEG	-3.2	45.5	49.0	19.3	0.0	0.0	0.0
SLC4A3	-5.6	243.5	160.7	647.6	0.0	0.0	0.0
ARMC9	-3.8	29.4	70.9	87.8	0.0	0.0	0.0
DGKD	2.6	1.3	8.3	0.0	254.2	592.3	20.7
RBM44	2.6	0.0	0.0	0.0	12.4	50.7	33.0
CAPN10	-4.6	69.6	235.8	90.7	0.0	0.0	0.0
HDLBP	2.5	86.9	291.1	133.2	1815.5	1144.7	895.6
NOP56	2.2	85.6	155.4	64.7	2927.8	413.6	130.1
CDC25B	3.2	0.0	0.0	0.0	32.8	102.3	30.2
KIF16B	2.8	0.0	0.0	0.0	50.5	14.2	96.2
ZNF337-AS	2.4	2.7	20.9	18.3	116.9	108.5	145.2
TM9SF4	2.4	0.0	2.1	0.0	26.6	30.2	16.0
NECAB3	-2.6	33.4	13.6	26.1	0.0	0.0	0.0
RALY	2.5	0.0	0.0	0.0	19.5	18.7	25.5
ACSS2	3.4	0.0	0.0	0.0	47.8	28.5	41.5
PTPRT	4.1	0.0	0.0	5.8	134.6	142.3	177.2
ZNFX1	2.7	0.0	0.0	0.0	16.8	32.9	97.1
RNF114	-2.7	78.9	38.6	12.5	0.0	0.0	0.0
NTSR1	-2.5	8.0	50.1	85.9	0.0	0.0	0.0
CCT8	2.5	0.0	4.2	0.0	34.5	36.5	90.5
CRYZL1	-2.2	131.1	28.2	93.6	0.0	6.2	9.4
MORC3	-2.3	8.0	33.4	31.8	0.0	0.0	0.0
DSCR3	-3.0	22.7	32.3	27.0	0.0	0.0	0.0
AGPAT3	2.4	0.0	0.0	1.0	9.7	38.2	30.2
AC006547.	2.5	0.0	0.0	0.0	11.5	93.4	32.1
TRMT2A	2.4	0.0	0.0	3.9	22.1	52.5	48.1
LRRC75B	2.1	18.7	27.1	5.8	100.1	94.3	147.1
SRRD	2.5	13.4	34.4	28.0	151.4	169.9	221.5
CTA-929C8	-2.2	187.3	152.3	228.7	30.1	40.0	39.6
CHEK2	2.3	0.0	0.0	1.0	15.9	20.5	14.1
DRG1	-3.0	40.1	21.9	154.4	0.0	0.0	0.0
CYTH4	-2.3	104.3	61.6	27.0	7.1	9.8	2.8
MKL1	3.4	0.0	0.0	0.0	25.7	207.2	545.8
ARHGAP8	2.1	0.0	4.2	6.8	35.4	32.0	47.1
TRABD	2.4	0.0	4.2	10.6	78.8	40.0	138.6
CHKB	-3.1	20.1	32.3	44.4	0.0	0.0	0.0
GRM7	3.3	0.0	0.0	0.0	19.5	339.8	205.5
TTLL3	3.1	0.0	0.0	0.0	15.1	105.8	136.7

IL17RC	2.2	0.0	0.0	0.0	10.6	40.0	19.8
CAPN7	3.7	0.0	0.0	0.0	63.8	82.7	31.1
EAF1-AS1	2.8	0.0	4.2	0.0	31.9	104.1	70.7
RARB	4.5	0.0	0.0	0.0	70.0	161.9	84.8
ZNF852	-2.8	37.5	60.5	14.5	0.0	0.0	0.0
LZTFL1	3.4	0.0	0.0	0.0	103.6	20.5	135.7
LTF	2.4	0.0	0.0	0.0	8.0	53.4	110.3
NPRL2	2.5	0.0	10.4	1.0	81.5	123.6	59.4
MAPKAPK3	-3.9	95.0	37.6	56.9	0.0	0.0	0.9
GRM2	3.0	0.0	0.0	0.0	58.5	17.8	40.5
ARF4	4.9	0.0	0.0	0.0	95.6	102.3	228.1
KCTD6	4.3	0.0	0.0	0.0	88.6	48.9	114.1
CLDND1	6.4	0.0	1.0	1.0	617.3	278.4	880.5
ST3GAL6	2.7	0.0	0.0	0.0	27.5	64.9	16.0
LSAMP	-2.1	19617.9	12519.1	2770.7	2627.6	1181.1	196.1
TIMMDC1	2.7	16.1	44.9	72.4	727.1	205.5	505.3
SLC41A3	-4.4	46.8	172.1	108.1	0.0	0.0	0.0
UBA5	3.1	0.0	0.0	1.0	24.8	33.8	104.6
TFDP2	3.0	0.0	0.0	0.0	41.6	38.2	19.8
PLOD2	-2.3	88.3	101.2	90.7	21.3	2.7	4.7
MFSD1	-3.0	49.5	662.5	338.7	0.0	0.0	5.7
MFSD1	4.0	1.3	0.0	0.0	101.8	38.2	128.2
DNAJC19	4.2	0.0	0.0	0.0	130.2	46.2	95.2
YEATS2-AS	4.4	0.0	0.0	0.0	66.4	137.9	73.5
DVL3	3.4	0.0	0.0	0.0	21.3	84.5	87.7
LINC00969	-2.6	20.1	59.5	92.6	4.4	0.9	0.0
FAM53A	-2.6	26.8	17.7	18.3	0.0	0.0	0.0
NELFA	2.6	0.0	4.2	0.0	141.7	26.7	63.2
NOP14-AS1	2.5	0.0	0.0	0.0	23.9	17.8	20.7
GABRA2	-5.5	571.2	328.6	115.8	0.0	0.0	0.0
NFXL1	2.6	0.0	3.1	0.0	32.8	31.1	42.4
PAQR3	3.6	0.0	0.0	2.9	58.5	64.9	66.0
LIN54	-4.2	74.9	114.8	41.5	0.0	0.0	0.0
ALPK1	-2.4	34.8	33.4	9.7	0.0	0.0	0.0
FHDC1	-5.7	248.8	196.1	119.7	0.0	0.0	0.0
FAM198B	2.3	0.0	0.0	0.0	17.7	12.5	43.4
TRAPPC11	-2.6	58.9	61.6	9.7	0.0	0.0	0.0
SORBS2	-3.9	28.1	165.9	205.6	0.0	0.0	0.0
MTRR	5.3	0.0	0.0	0.0	138.2	111.2	226.2
CTNND2	-3.8	476.2	46.9	1145.5	0.0	0.0	0.0
RNU6-760F	2.4	2.7	1.0	1.9	22.1	20.5	36.8
RP11-53O1	2.4	0.0	0.0	1.9	23.9	17.8	24.5
ERBB2IP	2.4	2.7	0.0	0.0	40.7	16.0	38.7
MAST4	2.3	0.0	0.0	0.0	46.9	43.6	7.5
RP11-60A8	-2.1	438.7	448.6	272.1	97.4	76.5	69.8
ANKRA2	2.3	0.0	3.1	3.9	29.2	27.6	39.6
VCAN	2.5	0.0	6.3	31.8	181.5	242.8	372.4

LINC00461	-2.1	460.2	228.5	88.8	62.0	12.5	28.3
RP11-213H	2.8	0.0	0.0	0.0	20.4	55.1	26.4
RHOBTB3	2.3	0.0	0.0	0.0	6.2	77.4	55.6
LINC01554	2.8	0.0	0.0	0.0	28.3	24.9	22.6
TMEM232	2.3	0.0	0.0	0.0	15.1	16.0	24.5
FBN2	3.4	0.0	0.0	0.0	26.6	49.8	65.0
AFF4	2.7	95.0	73.0	34.7	232.0	952.6	819.2
SMAD5	-2.1	64.2	31.3	69.5	3.5	12.5	6.6
HARS	2.9	0.0	0.0	6.8	70.8	119.2	101.8
FGF1	2.2	0.0	0.0	0.0	79.7	9.8	23.6
FAM114A2	-3.1	125.7	40.7	21.2	0.0	0.0	0.0
TSPAN17	-2.8	68.2	100.2	11.6	0.0	0.0	0.0
HNRNPH1	4.2	0.0	0.0	0.0	53.1	110.3	65.0
CANX	-2.5	21.4	14.6	23.2	0.0	0.0	0.0
ABCF1	3.0	12.0	0.0	0.0	217.9	267.7	267.7
AGPAT1	3.2	0.0	0.0	0.0	57.6	247.3	28.3
AGPAT1	2.2	0.0	0.0	0.0	13.3	53.4	14.1
HLA-DQA1	3.0	0.0	0.0	2.9	55.8	37.4	42.4
CCND3	2.1	0.0	10.4	13.5	98.3	79.2	62.2
PTK7	2.4	0.0	0.0	0.0	47.8	8.9	49.0
COL12A1	5.3	0.0	0.0	0.0	110.7	444.7	256.4
CEP162	2.1	18.7	4.2	17.4	132.8	104.9	46.2
CEP57L1	-3.5	28.1	45.9	55.0	0.0	0.0	0.0
DCBLD1	-2.6	89.6	168.0	110.0	8.0	0.0	0.0
SAMD3	-2.6	28.1	40.7	27.0	0.0	2.7	0.0
TNFAIP3	-2.4	16.1	40.7	13.5	0.0	0.0	0.0
SYNE1	-3.1	1561.0	1998.9	574.2	142.6	102.3	117.8
RP11-13P5	-3.7	50.8	80.3	65.6	0.0	0.9	2.8
FNDC1	-2.8	2288.7	4064.5	1893.4	128.4	385.1	338.4
SOD2	2.9	0.0	0.0	0.0	74.4	15.1	50.0
PARK2	-2.2	127.1	112.7	185.3	18.6	36.5	15.1
MLLT4	-2.6	323.7	295.2	42.5	13.3	0.0	7.5
C4B	-3.8	137.8	123.1	26.1	0.0	0.9	0.0
HLA-A	-3.3	28.1	45.9	30.9	0.0	0.0	0.0
ABCF1	3.0	12.0	0.0	0.0	217.9	267.7	267.7
EGFL8	-4.6	104.3	69.9	61.8	0.0	0.0	0.0
ABCF1	3.0	12.0	0.0	0.0	217.9	267.7	267.7
FLOT1	-4.9	97.6	100.2	70.4	0.0	0.0	0.0
BAG6	2.8	5.4	0.0	8.7	62.0	328.2	81.1
PBX2	3.0	0.0	0.0	2.9	36.3	39.1	52.8
C4B	-3.8	137.8	123.1	26.1	0.0	0.9	0.0
ICA1	2.4	0.0	0.0	0.0	8.0	56.0	48.1
NUPL2	-2.1	78.9	116.8	121.6	29.2	8.0	11.3
IGF2BP3	2.5	0.0	1.0	0.0	39.0	14.2	18.9
ELMO1	2.6	0.0	3.1	7.7	64.6	34.7	234.7
VPS41	-6.6	401.3	230.6	317.5	0.0	0.0	0.0
AEBP1	2.5	0.0	0.0	0.0	23.9	56.9	13.2

PPIA	2.0	12.0	4.2	4.8	25.7	78.3	47.1
UPP1	2.5	10.7	0.0	0.0	150.6	89.8	287.5
ABCA13	-3.4	25.4	108.5	50.2	0.0	0.0	0.0
GRB10	-2.8	72.2	18.8	22.2	0.0	0.9	0.0
LAT2	3.6	0.0	0.0	0.0	29.2	68.5	72.6
CACNA2D1	-2.2	349.1	58.4	29.0	7.1	16.9	12.3
KIAA1324L	4.4	0.0	0.0	0.0	255.1	216.1	49.0
AKAP9	4.5	0.0	0.0	0.0	137.3	54.3	179.1
ARPC1B	2.8	0.0	0.0	0.0	32.8	21.3	22.6
GNB2	-2.5	84.3	109.5	256.7	0.0	14.2	8.5
CUX1	6.1	0.0	0.0	0.0	352.5	188.6	297.9
RASA4	3.8	0.0	0.0	0.0	42.5	210.8	71.6
PSMC2	-3.6	173.9	58.4	31.8	0.0	0.0	0.0
HBP1	4.1	0.0	0.0	0.0	51.4	58.7	102.8
DGKI	2.0	34.8	18.8	21.2	156.8	54.3	268.7
FASTK	2.7	0.0	0.0	0.0	115.1	24.9	21.7
NCAPG2	4.2	0.0	0.0	0.0	50.5	81.8	67.9
MYOM2	2.5	2.7	4.2	1.9	25.7	32.9	46.2
ATP6V1B2	2.3	0.0	8.3	0.0	58.5	458.9	68.8
CCAR2	6.0	0.0	0.0	0.0	353.4	246.4	187.6
INTS9	-2.4	53.5	43.8	15.4	0.0	0.0	2.8
RP11-363L	2.2	0.0	0.0	0.0	13.3	16.0	24.5
ZFAND1	2.4	0.0	0.0	0.0	5.3	148.5	125.4
TMEM64	2.3	0.0	0.0	0.0	16.8	24.0	15.1
RPL30	-4.4	128.4	52.2	77.2	0.0	0.0	0.0
SQLE	-2.8	111.0	92.8	10.6	0.0	0.0	0.0
KIAA0196	-4.2	96.3	92.8	39.6	0.0	0.0	0.0
KHDRBS3	3.9	0.0	0.0	0.0	76.2	73.8	36.8
RP11-149P	-2.9	120.4	66.8	169.9	14.2	8.9	3.8
AGO2	-2.2	123.1	49.0	51.1	9.7	0.0	4.7
PTK2	3.4	0.0	0.0	0.0	44.3	27.6	72.6
PUF60	3.0	0.0	0.0	0.0	106.3	27.6	28.3
MFSD3	4.3	0.0	0.0	0.0	52.3	132.5	81.1
SLC1A1	2.5	0.0	0.0	1.9	15.1	34.7	37.7
SPATA6L	2.2	4.0	7.3	14.5	61.1	55.1	73.5
RP11-408A	2.1	4.0	2.1	4.8	31.0	20.5	44.3
IL11RA	2.5	0.0	0.0	0.0	27.5	23.1	15.1
UNC13B	-3.7	536.4	434.0	86.9	0.0	1.8	8.5
RP11-305L	2.9	0.0	0.0	0.0	23.9	38.2	24.5
NOL8	-2.9	18.7	26.1	48.3	0.0	0.0	0.0
DAB2IP	5.2	0.0	0.0	1.0	138.2	150.3	361.1
FIBCD1	-2.3	694.2	2432.9	1316.3	329.4	178.8	163.1
MSL3	-3.2	25.4	139.8	41.5	0.0	0.0	0.0
SAT1	3.4	0.0	0.0	0.0	57.6	44.5	315.8
DDX3X	-6.6	317.0	269.2	551.0	0.0	0.0	0.0
RBM3	-4.5	116.4	66.8	63.7	0.0	0.0	0.0
PQBP1	4.3	0.0	0.0	0.0	341.8	125.4	64.1

BTK	-2.3	32.1	21.9	10.6	0.0	0.0	0.0
SLC6A8	2.4	0.0	0.0	0.0	10.6	33.8	40.5
FAM223A	-2.3	28.1	9.4	24.1	0.0	0.0	0.0
FAM223B	2.4	0.0	0.0	0.0	16.8	28.5	16.0
MPP1	2.4	0.0	2.1	4.8	36.3	53.4	25.5
RPS4Y1	-2.2	5.4	157.5	159.2	0.0	2.7	0.0
ZFY	-2.5	10.7	98.1	70.4	0.9	4.4	0.9
DDX3Y	-2.3	34.8	486.2	378.3	16.8	0.0	1.9
NLGN4Y	-2.6	40.1	700.0	587.7	20.4	38.2	23.6
FERMT2	2.7	0.0	0.0	0.0	25.7	17.8	34.9
RYR3	-2.6	13.4	656.2	107.1	2.7	0.0	2.8
BFAR	-2.3	22.7	10.4	23.2	0.0	0.0	0.0
NDE1	2.3	4.0	9.4	16.4	32.8	109.4	145.2
FBF1	-3.7	73.6	26.1	99.4	0.0	0.0	0.0
ARHGAP23	2.4	6.7	2.1	6.8	2.7	640.4	420.4
KANSL1	4.2	0.0	0.0	0.0	54.9	64.9	120.7
QARS	2.4	0.0	0.0	0.0	15.9	58.7	16.0
FAM20C	3.2	0.0	0.0	0.0	27.5	32.0	58.4
MYOM2	2.5	2.7	4.2	1.9	25.7	32.9	46.2

Supplementary Table S5: Differential gene expression and transcript expression

Gene name	Type	Transcript	Fold change in transcript level	Fold change in gene level
VPS41	Known DG	ENST00000457055	-10.86	-6.57
KHDRBS3	Known DG	ENST00000518728	10.36	3.89
CMC2	Novel DG	ENST00000569187	-9.84	-6.30
HELZ	Novel DG	ENST00000581159	9.57	6.00
RNU6-760P	Novel DG	ENST00000516078	3.89	2.38
PGBD2	Novel DG	ENST00000462488	8.75	3.35
CAMKK2	Known DG	ENST00000402834	9.47	2.50
GGA3	Known DG	ENST00000584550	8.76	5.23
MEST	Novel DG	ENST00000416162	8.54	7.38
MLST8	Novel DG	ENST00000562479	-9.09	-4.87
TAOK3	Novel DG	ENST00000543709	8.45	5.01
SYNPR	Novel DG	ENST00000478300	-8.95	-7.07
LPIN2	Novel DG	ENST00000584915	8.74	4.38
LSM14A	Novel DG	ENST00000624888	10.00	3.14
SAT1	Known DG	ENST00000487713	9.50	3.42
CLDND1	Known DG	ENST00000510545	8.53	6.38
WDR74	Known DG	ENST00000538098	2.47	2.27
COQ9	Known DG	ENST00000563391	-4.68	-3.47
SYNE1	Known DG	ENST00000474655	-3.51	-3.06
FLOT1	Novel DG	ENST00000457133	-8.14	-4.87
C17orf49	Novel DG	ENST00000439424	-8.64	-4.85
CCAR2	Novel DG	ENST00000613179	8.02	6.04
SLC41A3	Known DG	ENST00000514023	-8.56	-4.38
RPL30	Known DG	ENST00000517489	-8.28	-4.41
MINOS1	Novel DG	ENST00000462646	-8.59	-2.72
APEX1	Known DG	ENST00000554325	-9.03	-3.60
DDX23	Known DG	ENST00000551468	8.58	3.89
MDM2	Known DG	ENST00000350057	-7.78	-5.29
SVBP	Novel DG	ENST00000372522	9.22	3.48
WDR18	Known DG	ENST00000585809	-8.05	-6.05
BRD2	Novel DG	ENST00000471151	7.68	4.01
EFEMP1	Known DG	ENST00000440439	8.11	3.99
PQBP1	Known DG	ENST00000470062	8.51	4.34
ISCU	Novel DG	ENST00000547005	7.99	4.74
GSTP1	Known DG	ENST00000398603	8.32	4.25
TBRG1	Known DG	ENST00000530731	-7.99	-5.95
HBP1	Known DG	ENST00000461963	7.82	-4.57
VPS53	Known DG	ENST00000575207	-2.30	-2.11
SLC6A8	Known DG	ENST00000467402	8.15	2.44
BRF1	Known DG	ENST00000547530	-7.38	3.26

DIDO1	Novel DG	ENST00000354665	8.15	6.31
SRRD	Known DG	ENST00000477945	2.81	2.46
CLK3	Known DG	ENST00000563297	7.60	3.88
CBSL	Novel DG	ENST00000398168	7.97	6.26
MXRA7	Novel DG	ENST00000449428	-7.60	-4.89
DAB2IP	Novel DG	ENST00000436835	7.52	5.23
SNORD114-20	Novel DG	ENST00000365178	-4.70	-3.21
SLC25A3	Known DG	ENST00000552981	-7.87	-5.51
ICA1	Known DG	ENST00000486677	8.42	2.37
IVD	Novel DG	ENST00000559575	8.47	2.22
ROGDI	Known DG	ENST00000591392	-8.84	-3.67
RPLP2	Known DG	ENST00000532004	-7.66	-2.59
GMPR2	Novel DG	ENST00000559409	7.89	3.90
NOP14-AS1	Novel DG	ENST00000512802	7.47	2.51
ARMC9	Novel DG	ENST00000424740	-8.01	-3.79
HDAC9	Known DG	ENST00000401921	-8.13	-6.22
FNDC1	Novel DG	ENST00000329629	-3.25	-2.85
NDRG4	Known DG	ENST00000564960	-7.70	-4.73
SHMT2	Known DG	ENST00000557703	-8.47	-3.18
SORBS2	Known DG	ENST00000419063	-8.37	-3.85
ENOX1	Known DG	ENST00000482207	5.83	3.46
RBM3	Known DG	ENST00000488216	-7.66	-4.53
RC3H1	Novel DG	ENST00000484867	6.17	2.97
PDHB	Known DG	ENST00000383714	-7.94	-5.17
NDRG2	Known DG	ENST00000553900	-6.63	-2.84
PTPRT	Known DG	ENST00000620410	6.17	4.07
PLD2	Novel DG	ENST00000576983	8.24	3.27
PSMC2	Known DG	ENST00000457587	-8.15	-3.56
AFF4	Known DG	ENST00000378593	3.31	2.65
MFSD1	Known DG	ENST00000471266	7.25	3.97
HDLBP	Known DG	ENST00000422933	2.90	2.49
GLDN	Novel DG	ENST00000558426	-8.53	-2.94
TTLL3	Novel DG	ENST00000422738	8.23	3.08
DDX3X	Known DG	ENST00000630255	-7.59	-6.60
OS9	Known DG	ENST00000550372	8.92	2.40
FECH	Novel DG	ENST00000585747	-7.38	-3.59
ATXN7	Novel DG	ENST00000538065	7.39	7.36
HNRNPL	Known DG	ENST00000598985	2.49	2.23
TAF6	Novel DG	ENST00000344095	6.33	3.55
MEGF11	Novel DG	ENST00000455812	-3.11	-2.60
NAPA	Known DG	ENST00000595227	-8.15	-4.37
RBM23	Known DG	ENST00000346528	8.09	4.79
EIF2D	Known DG	ENST00000613435	7.57	2.79
RASA4	Novel DG	ENST00000521397	7.73	3.78
HDAC9	Known DG	ENST00000524023	-4.70	-6.22
HARS	Novel DG	ENST00000512396	5.25	2.94
TIMMDC1	Novel DG	ENST00000493694	3.39	2.66

GMIP	Novel DG	ENST00000591047	5.01	3.63
DPP9	Known DG	ENST00000600621	7.34	3.56
SIRT1	Known DG	ENST00000497639	7.41	3.65
DVL3	Novel DG	ENST00000467873	7.55	3.35
BRF1	Known DG	ENST00000619151	5.97	3.26
TMEM25	Novel DG	ENST00000533587	-8.02	-2.86
PDE2A	Known DG	ENST00000538749	8.40	2.56
STARD13-AS	Novel DG	ENST00000589122	5.04	2.80
C4B	Novel DG	ENST00000487226	-7.79	-3.80
C4B	Novel DG	ENST00000488817	-7.79	-3.80
SEH1L	Known DG	ENST00000589446	6.53	3.95
CMC2	Novel DG	ENST00000566918	7.18	-6.30
MVP	Known DG	ENST00000563915	-7.30	-4.18
CAPS	Novel DG	ENST00000590428	-7.34	-2.95
DVL3	Novel DG	ENST00000478639	2.17	3.35
HSPA8	Novel DG	ENST00000526110	4.80	3.08
NBL1	Known DG	ENST00000439278	7.29	-5.02
SMUG1	Novel DG	ENST00000511522	-7.32	-3.18
SLC2A5	Known DG	ENST00000484798	-7.80	-3.15
BBS1	Known DG	ENST00000532908	-7.15	-4.55
SH2B1	Novel DG	ENST00000567536	7.99	2.36
VCAN	Novel DG	ENST00000505615	4.32	2.49
SAT2	Novel DG	ENST00000570914	6.72	3.79
TMPO	Known DG	ENST00000546828	6.19	3.14
CTSF	Known DG	ENST00000525733	3.74	2.68
PHTF1	Novel DG	ENST00000369604	-6.69	-4.27
ACTR6	Known DG	ENST00000548180	-5.79	-3.16
LTF	Known DG	ENST00000493056	7.89	2.41
RAB4B	Novel DG	ENST00000378307	-7.96	-3.27
PTK2	Known DG	ENST00000523435	7.08	3.39
UPP1	Known DG	ENST00000417464	5.81	2.50
ZFY	Novel DG	ENST00000443793	-8.64	-2.51
A2M	Novel DG	ENST00000495709	7.05	2.89
FGF13	Known DG	ENST00000305414	-7.63	-4.65
YEATS2-AS1	Novel DG	ENST00000609195	6.97	4.44
ZNF627	Novel DG	ENST00000361113	-2.65	-2.41
DDX39B	Novel DG	ENST00000471062	7.33	5.50
WARS	Known DG	ENST00000557135	-7.52	-4.80
CDC25B	Known DG	ENST00000468979	7.26	3.18
TESPA1	Novel DG	ENST00000532757	2.89	2.35
SOD2	Novel DG	ENST00000535561	7.31	2.89
TRAPPC11	Known DG	ENST00000513600	-7.61	-2.63
OR2H2	Novel DG	ENST00000439874	-7.38	-4.59
SATB2	Known DG	ENST00000484124	6.77	3.30
SLC39A1	Novel DG	ENST00000537590	-3.87	-2.62
ZNF337-AS1	Novel DG	ENST00000455791	3.03	2.39
SH3YL1	Known DG	ENST00000454318	-7.75	-2.61

METTL25	Novel DG	ENST00000550058	-4.63	-2.75
ZNF682	Novel DG	ENST00000593468	7.28	2.92
SEZ6	Novel DG	ENST00000360295	-7.26	-6.19
RHOBTB3	Known DG	ENST00000511558	7.69	2.68
TLDC1	Novel DG	ENST00000565765	-7.03	-3.02
DCBLD1	Known DG	ENST00000424717	-5.43	-2.64
RPP21	Novel DG	ENST00000418043	-8.42	2.28
SFXN5	Known DG	ENST00000495208	-7.01	-3.79
GABRA2	Known DG	ENST00000510861	-7.35	-5.48
CAPN7	Known DG	ENST00000443567	6.90	3.67
ARF4	Known DG	ENST00000486310	6.92	4.86
RNF114	Known DG	ENST00000625177	-7.46	-2.71
USH1C	Known DG	ENST00000318024	-3.81	-2.89
UBA5	Novel DG	ENST00000464068	7.18	3.10
CSRP1	Known DG	ENST00000529975	7.03	2.86
RARB	Novel DG	ENST00000383772	6.88	4.55
CTNND2	Known DG	ENST00000504499	-8.02	-3.84
TCP11L2	Novel DG	ENST00000553098	7.13	3.73
MSL3	Novel DG	ENST00000421368	-7.50	-3.20
FAM114A2	Novel DG	ENST00000522395	-7.44	-3.07
EXOSC5	Known DG	ENST00000596905	7.31	3.27
DRG1	Known DG	ENST00000433341	-7.56	-3.01
OPTN	Known DG	ENST00000486862	2.92	2.30
KIF16B	Known DG	ENST00000355755	7.30	2.82
CDK2AP1	Known DG	ENST00000544658	1.85	2.79
CCT8	Known DG	ENST00000480359	5.28	2.54
PPP2R3C	Known DG	ENST00000553282	2.69	2.04
IL12RB2	Novel DG	ENST00000371000	-2.21	-2.03
ATP6V1B2	Known DG	ENST00000523478	6.03	2.27
WDR86	Novel DG	ENST00000621812	-6.33	-2.70
OSBPL8	Novel DG	ENST00000551927	7.31	2.48
MUM1	Known DG	ENST00000586996	2.34	2.03
SPHK2	Known DG	ENST00000593308	-2.31	-2.05
NOL8	Novel DG	ENST00000411621	-7.05	-2.88
UGP2	Novel DG	ENST00000494536	6.09	3.19
MRPL55	Novel DG	ENST00000476267	8.14	3.03
CADM1	Known DG	ENST00000542447	-6.93	-6.46
M6PR	Known DG	ENST00000543845	-4.81	-2.45
HLA-DQA1	Novel DG	ENST00000422863	5.10	3.04
PAQR3	Known DG	ENST00000515541	6.01	3.64
S100A6	Known DG	ENST00000496817	-6.82	-3.32
RPP21	Novel DG	ENST00000415583	7.41	2.28
NDE1	Novel DG	ENST00000574163	3.23	2.26
VPS51	Novel DG	ENST00000526856	7.31	2.77
SORT1	Known DG	ENST00000485149	-6.80	-2.30
LRRC37B	Novel DG	ENST00000582117	5.16	2.20
SLC4A3	Known DG	ENST00000373760	-7.13	-5.62

MFSD1	Known DG	ENST00000622669	-7.02	3.97
LONP1	Known DG	ENST00000590206	4.59	2.32
CPSF7	Novel DG	ENST00000489728	6.57	2.48
ZBTB7C	Novel DG	ENST00000588028	-7.19	-2.46
SYNE1	Known DG	ENST00000481502	-1.41	-3.06
DNAJC19	Novel DG	ENST00000469657	6.71	4.23
RBM17	Novel DG	ENST00000437845	-7.79	-2.55
VPS41	Known DG	ENST00000418457	2.33	-6.57
TNNT2	Known DG	ENST00000422165	5.83	2.38
ANAPC15	Known DG	ENST00000543015	6.50	2.60
CHMP1A	Known DG	ENST00000547614	7.12	2.60
AARSD1	Novel DG	ENST00000450475	-7.51	-2.38
CNTFR	Novel DG	ENST00000378980	4.04	2.54
EBPL	Novel DG	ENST00000378270	-5.83	-2.75
HNRNPH1	Known DG	ENST00000505811	6.58	4.20
DPF1	Known DG	ENST00000355526	-7.19	-3.98
ELMO1	Known DG	ENST00000455879	4.74	2.60
ABCF1	Novel DG	ENST00000475993	5.88	2.98
ABCF1	Novel DG	ENST00000494413	5.88	2.98
ABCF1	Novel DG	ENST00000486105	5.88	2.98
DDX3Y	Known DG	ENST00000495478	-5.60	-2.66
ST3GAL6	Novel DG	ENST00000460774	6.83	-2.85
B3GALNT1	Known DG	ENST00000392781	3.60	2.77
SPAG5	Known DG	ENST00000584206	2.59	2.16
SEMA4A	Novel DG	ENST00000435124	-6.67	-4.13
SHKBP1	Novel DG	ENST00000602239	-4.25	-2.43
SLC50A1	Known DG	ENST00000368405	7.28	3.07
PPP2R1B	Known DG	ENST00000531890	6.58	3.04
COG6	Novel DG	ENST00000356576	3.36	2.67
INTS7	Known DG	ENST00000366993	7.04	4.78
XRCC1	Known DG	ENST00000598165	-7.23	-3.00
KPTN	Known DG	ENST00000595554	6.65	3.35
DDX3Y	Known DG	ENST00000360160	-4.35	-2.66
CHKB	Novel DG	ENST00000471515	-6.76	-3.05
TAC1	Known DG	ENST00000350485	-2.58	-2.16
QARS	Known DG	ENST00000634425	6.84	2.36
CUX1	Novel DG	ENST00000556210	6.47	6.13
SRSF11	Known DG	ENST00000469170	6.98	2.67
LMO4	Known DG	ENST00000489303	3.74	2.51
DOCK6	Known DG	ENST00000592403	3.33	2.26
MDM1	Known DG	ENST00000541686	5.91	3.12
ZFAND1	Novel DG	ENST00000522032	7.58	2.36
POSTN	Novel DG	ENST00000473823	-3.00	-3.12
RMND5B	Novel DG	ENST00000313386	-6.83	-4.59
CDK5RAP3	Novel DG	ENST00000584042	7.00	2.46
NBL1	Known DG	ENST00000615215	-6.68	-5.02
NDUFAF3	Novel DG	ENST00000451378	-7.01	-2.79

TAF1C	Novel DG	ENST00000561955	6.42	2.74
TUBB4A	Known DG	ENST00000600216	-2.90	-2.15
DDX3Y	Known DG	ENST00000440554	-6.93	-2.66
RSL1D1	Known DG	ENST00000355674	-7.65	-2.72
TNNC2	Known DG	ENST00000372555	-6.94	-2.73
DEF8	Known DG	ENST00000570182	-7.48	-3.85
KDELR2	Known DG	ENST00000490996	6.70	2.79
TSPAN17	Novel DG	ENST00000504168	-7.10	-2.83
GNG4	Novel DG	ENST00000484517	6.89	2.55
MRPL55	Novel DG	ENST00000336300	-6.72	3.03
CEP112	Novel DG	ENST00000581739	-6.54	-2.26
TRABD2A	Known DG	ENST00000460991	6.47	2.46
MAPKAPK3	Known DG	ENST00000430409	-6.67	-3.90
KARS	Known DG	ENST00000568378	-6.02	-2.26
DDX3Y	Known DG	ENST00000463199	-7.70	-2.66
PLOD2	Novel DG	ENST00000469350	-3.28	-2.28
MFSD3	Novel DG	ENST00000528047	6.45	4.28
CCND3	Novel DG	ENST00000513956	3.22	2.11
ABLIM1	Known DG	ENST00000440467	5.55	2.77
IGF2BP3	Known DG	ENST00000495009	5.88	2.46
PEX19	Novel DG	ENST00000472750	-2.99	-2.73
EAF1-AS1	Novel DG	ENST00000599742	5.40	2.84
PSEN1	Known DG	ENST00000559361	6.40	3.17
TTC39A	Novel DG	ENST00000439482	-6.72	-3.11
MRPL49	Novel DG	ENST00000531705	3.88	3.18
LRRC37A	Novel DG	ENST00000625784	6.47	5.85
NAPA	Known DG	ENST00000601927	6.56	-4.37
SPINT2	Novel DG	ENST00000587516	-6.65	-2.96
H3F3B	Novel DG	ENST00000593254	-6.57	-3.42
RPS6KL1	Novel DG	ENST00000553315	6.51	2.60
GAD1	Known DG	ENST00000429023	6.90	2.68
NTM	Known DG	ENST00000498764	6.45	2.48
KDM4B	Known DG	ENST00000588166	4.57	2.39

Supplementary Table S6: Hippocampus specific functional module analysis

MODULE	TOP TERMS	Q VAL	GENES	TERMS
M1	pri-miRNA transcription by RNA polymerase II	0.00396721	206	275
	N-terminal peptidyl-lysine acetylation	0.00481550		
	cellular response to nitrogen compound	0.00542073		
	regulation of angiogenesis	0.00720272		
	negative regulation of centriole replication	0.00720272		
	regulation of vasculature development	0.00820990		
	intracellular receptor signaling pathway	0.00820990		
	cellular response to UV	0.00820990		
	cellular response to light stimulus	0.00820990		
	cellular response to organic cyclic compound	0.00876711		
M2	response to peptide	0.00481550	6	31
	cellular response to peptide	0.00481550		
	response to interleukin-6	0.00542073		
	cellular response to interleukin-6	0.00542073		
	cellular response to organonitrogen compound	0.00776988		
	cellular response to nitrogen compound	0.00932345		
	response to lipid	0.01058727		
	response to organonitrogen compound	0.01058727		
	cellular response to lipopolysaccharide	0.01461036		
	cellular response to peptide hormone stimulus	0.01461036		
M3	endocrine system development	0.00542073	205	197
	sensory organ development	0.00587629		
	striated muscle contraction	0.00820990		
	muscle contraction	0.00820990		
	muscle system process	0.01058727		
	branching involved in ureteric bud morphogenesis	0.01058727		
	iris morphogenesis	0.01058727		
	ureteric bud morphogenesis	0.01208289		
	mesonephric tubule morphogenesis	0.01208289		
	embryonic morphogenesis	0.01408814		

MODULE	TOP TERMS	Q VAL	GENES	TERMS
M4	regulation of lipid transport by negative regulation of transcription from RNA polymerase II promoter	0.00542073	78	86
	DNA-templated transcription, initiation	0.00977262		
	regulation of lipid transport by regulation of transcription from RNA polymerase II promoter	0.01058727		
	response to endoplasmic reticulum stress	0.01509087		
	RNA polymerase I transcriptional preinitiation complex assembly	0.01560842		
	transcription initiation from RNA polymerase I promoter	0.01736905		
	negative regulation of ERAD pathway	0.02409167		
	negative regulation of cellular protein catabolic process	0.02584921		
	DNA-templated transcriptional preinitiation complex assembly	0.02749498		
	regulation of protein binding	0.03027071		
M5	response to virus	0.00878487	8	10
	cellular response to mechanical stimulus	0.01173156		
	response to mechanical stimulus	0.01522546		
	defense response to virus	0.02394245		
	I-kappaB kinase/NF-kappaB signaling	0.02650456		
	cellular response to external stimulus	0.02691787		
	cellular response to environmental stimulus	0.03027071		
	cellular response to abiotic stimulus	0.03027071		
	defense response to other organism	0.03852634		
	immune effector process	0.04605383		
M6	gene silencing	0.01058727	25	32
	positive regulation of DNA metabolic process	0.01522546		
	positive regulation of DNA repair	0.02083722		
	regulation of gene silencing	0.02209560		
	gene silencing by miRNA	0.02633943		
	posttranscriptional gene silencing by RNA	0.02650456		
	gene silencing by RNA	0.02650456		
	posttranscriptional gene silencing	0.02650456		
	positive regulation of response to DNA damage stimulus	0.02749498		
	DNA repair	0.03027071		

MODULE	TOP TERMS	Q VAL	GENES	TERMS
M7	DNA methylation or demethylation	0.01208289	15	14
	DNA modification	0.01461036		
	peptide metabolic process	0.02628624		
	gene silencing	0.02650456		
	regulation of gene expression, epigenetic	0.03059711		
	regulation of mRNA metabolic process	0.03533313		
	nucleotide metabolic process	0.06078681		
	nucleoside phosphate metabolic process	0.06162109		
	posttranscriptional regulation of gene expression	0.06202261		
	translation	0.06449846		
M8	epithelium development	0.01461036	2	1
M9	negative regulation of protein phosphorylation	0.01559786	3	2
	negative regulation of phosphorylation	0.01651564		
M10	cellular response to hexose stimulus	0.01826775	29	49
	cellular response to glucose stimulus	0.01826775		
	cellular response to monosaccharide stimulus	0.01826775		
	cellular glucose homeostasis	0.01849225		
	glucose import	0.01849225		
	cellular response to carbohydrate stimulus	0.01936591		
	cellular response to growth factor stimulus	0.02394245		
	response to glucose	0.02409167		
	response to hexose	0.02439405		
	response to monosaccharide	0.02439405		
M11	endocytosis	0.02650456	59	29
	Ras protein signal transduction	0.02749498		
	regulation of Rho protein signal transduction	0.02749498		
	import into cell	0.03027071		
	small GTPase mediated signal transduction	0.03059711		
	lymphocyte migration	0.03318096		
	endothelial cell migration	0.03665687		
	positive regulation of GTPase activity	0.03770258		
	Rho protein signal transduction	0.03920565		
	regulation of GTPase activity	0.04696612		

MODULE	TOP TERMS	Q VAL	GENES	TERMS
M12	regulation of mitochondrial translation	0.02812692	91	55
	defense response to virus	0.02906631		
	peptide metabolic process	0.03360217		
	defense response to other organism	0.03781772		
	regulation of TOR signaling	0.03852634		
	translation	0.04048259		
	ATP metabolic process	0.04399610		
	peptide biosynthetic process	0.04399610		
	negative regulation of stress-activated MAPK cascade	0.04423612		
	negative regulation of stress-activated protein kinase signaling cascade	0.04423612		
M13	negative regulation of proteolysis	0.05322028	18	1

Supplementary Table S7: Functional enrichment study of predicted variants, TF and differentially expressed genes

Gene name	Gene expression	Type	Enriched biological function
APEX1	DOWN	Known DG	aging
APP	UP	variant	aging
ATF7IP	UP	Known DG	aging
BCL2	UP	TF	aging
CACNA1C	UP	Novel DG	aging
CANX	DOWN	Known DG	aging
CASP7	UP	Novel DG	aging
CASP9	UP	Novel DG	aging
CDK2	DOWN	TF	aging
CHEK2	UP	Known DG	aging
DMD	UP	Novel	aging
EIF2A	UP	Novel DG	aging
ERCC1	UP	Known DG	aging
ILK	DOWN	variant	aging
JUN	DOWN	TF	aging
LONP1	UP	Known DG	aging
MAP3K3	DOWN	Novel DG	aging
MORC3	DOWN	Known DG	aging
NFKB2	DOWN	TF	aging
PAX5	DOWN	TF	aging
PICALM	UP	variant	aging
POLD1	DOWN	Known DG	aging
PSEN1	UP	Known DG	aging
RSL1D1	DOWN	Known DG	aging
SIRT1	UP	Known DG	aging
SNAP91	UP	variant	aging
SOD2	UP	Novel DG	aging
SRF	DOWN	TF	aging
STAT3	DOWN	TF	aging
ULK3	DOWN	Novel DG	aging
ATF2	UP	Known DG	AKT signaling
BCL2	UP	TF	AKT signaling
BRCA1	DOWN	TF	AKT signaling
CASP9	UP	Novel DG	AKT signaling
CCND3	UP	Novel DG	AKT signaling
CDK2	DOWN	TF	AKT signaling
FGF1	UP	Known DG	AKT signaling
GNB2	DOWN	Novel DG	AKT signaling
GNG4	UP	Novel DG	AKT signaling
GNGT2	DOWN	Novel DG	AKT signaling
IFNAR2	DOWN	Novel DG	AKT signaling

LAMA5	DOWN	variant	AKT signaling
LAMB2	DOWN	Novel DG	AKT signaling
MAGI2	UP	variant	AKT signaling
MDM2	DOWN	Known DG	AKT signaling
MLST8	DOWN	Novel DG	AKT signaling
MYC	DOWN	TF	AKT signaling
NFKB1	DOWN	TF	AKT signaling
NTRK2	UP	variant	AKT signaling
PIK3R2	UP	Novel DG	AKT signaling
PPP2R1B	UP	Known DG	AKT signaling
PPP2R3C	UP	Known DG	AKT signaling
PTK2	UP	Known DG	AKT signaling
RXRA	DOWN	TF	AKT signaling
TNN	UP	Novel DG	AKT signaling
TSC2	UP	Novel DG	AKT signaling
APP	UP	variant	amyloid
CDK2	DOWN	TF	amyloid
MDM2	DOWN	Known DG	amyloid
PSEN1	UP	Known DG	amyloid
ABCA7	DOWN	Known DG	amyloid
APEX1	DOWN	Known DG	amyloid
NFYA	DOWN	TF	amyloid
PICALM	UP	variant	amyloid
SNAP91	UP	variant	amyloid
ACE	DOWN	variant	amyloid
DCP1B	UP	Novel DG	amyloid
ADAM8	UP	Known DG	apoptosis
AES	DOWN	Novel DG	apoptosis
AIPL1	DOWN	Novel DG	apoptosis
APEX1	DOWN	Known DG	apoptosis
APP	UP	variant	apoptosis
ARF4	UP	Known DG	apoptosis
ARHGEF3	UP	variant	apoptosis
ATF2	UP	Known DG	apoptosis
ATF7IP	UP	Known DG	apoptosis
BAG6	UP	Novel DG	apoptosis
BCL11B	UP	variant	apoptosis
BCL2	UP	TF	apoptosis
BCL3	DOWN	TF	apoptosis
BFAR	DOWN	Known DG	apoptosis
BRCA1	DOWN	TF	apoptosis
BRF1	UP	TF	apoptosis
BRMS1	UP	Known DG	apoptosis
BTK	DOWN	Known DG	apoptosis
CADM1	DOWN	Known DG	apoptosis
CAPN10	DOWN	Novel DG	apoptosis
CARD9	DOWN	Novel DG	apoptosis

CASP4	UP	Known DG	apoptosis
CASP7	UP	Novel DG	apoptosis
CASP9	UP	Novel DG	apoptosis
CCAR2	UP	Novel DG	apoptosis
CD46	UP	Known DG	apoptosis
CDK2	DOWN	TF	apoptosis
CDK5RAP3	UP	Novel DG	apoptosis
CEP57	UP	Known DG	apoptosis
CHEK2	UP	Known DG	apoptosis
CNTFR	UP	Novel DG	apoptosis
CREBBP	UP	TF	apoptosis
CRIP2	UP	Novel DG	apoptosis
CSRP1	UP	Known DG	apoptosis
CYLD	DOWN	Known DG	apoptosis
DAB2IP	UP	Novel DG	apoptosis
DDX3X	DOWN	Known DG	apoptosis
DIDO1	UP	TF-Novel	apoptosis
DIP2A	UP	Known DG	apoptosis
DNMT1	UP	TF	apoptosis
DPF1	DOWN	Known DG	apoptosis
DRG1	DOWN	Known DG	apoptosis
DYNLL1	UP	variant	apoptosis
E2F1	DOWN	TF	apoptosis
E2F3	DOWN	TF	apoptosis
EIF2A	UP	Novel DG	apoptosis
ELMO1	UP	Known DG	apoptosis
ESR1	DOWN	TF	apoptosis
EYA3	UP	Novel DG	apoptosis
FASTK	UP	Novel DG	apoptosis
FXR1	DOWN	TF	apoptosis
GATA2	DOWN	TF	apoptosis
GATA3	DOWN	TF	apoptosis
GATA6	DOWN	TF	apoptosis
GRIK2	UP	variant	apoptosis
GSTP1	UP	Known DG	apoptosis
HDAC1	DOWN	TF	apoptosis
HOXA5	DOWN	TF	apoptosis
HSF1	DOWN	TF	apoptosis
IER3	UP	Novel DG	apoptosis
ILK	DOWN	variant	apoptosis
IP6K2	DOWN	Novel DG	apoptosis
IRF1	DOWN	TF	apoptosis
IRF3	DOWN	TF	apoptosis
IRF5	DOWN	TF	apoptosis
IRF7	UP	TF-Novel	apoptosis
JAG2	UP	Novel DG	apoptosis
JUN	DOWN	TF	apoptosis

LONP1	UP	Known DG	apoptosis
LTF	UP	Known DG	apoptosis
MAG	UP	variant	apoptosis
MAGI2	UP	variant	apoptosis
MDM2	DOWN	Known DG	apoptosis
MEF2A	UP	TF	apoptosis
MEN1	UP	Novel DG	apoptosis
MPP1	UP	Novel DG	apoptosis
MTCH1	UP	variant	apoptosis
MXI1	UP	TF	apoptosis
MYC	DOWN	TF	apoptosis
NECAB3	DOWN	Known DG	apoptosis
NF1	DOWN	TF	apoptosis
NFKB1	DOWN	TF	apoptosis
NR2C2	DOWN	TF	apoptosis
NR3C1	UP	TF	apoptosis
NTRK2	UP	variant	apoptosis
NTSR1	DOWN	Novel DG	apoptosis
OPTN	UP	Known DG	apoptosis
PAX3	DOWN	TF	apoptosis
PAX8	DOWN	TF	apoptosis
PDCD6IP	UP	Known DG	apoptosis
PGAP2	DOWN	Novel DG	apoptosis
POLD1	DOWN	Known DG	apoptosis
PPP2R1B	UP	Known DG	apoptosis
PRKACA	UP	Known DG	apoptosis
PRKCE	DOWN	variant	apoptosis
PRKRA	UP	Novel DG	apoptosis
PSEN1	UP	Known DG	apoptosis
PTK2	UP	Known DG	apoptosis
PUF60	UP	Novel DG	apoptosis
QARS	UP	Known DG	apoptosis
RABGGTA	UP	Novel DG	apoptosis
RAD21	DOWN	TF	apoptosis
RARB	UP	Novel DG	apoptosis
RBBP8	UP	Known DG	apoptosis
REST	DOWN	TF	apoptosis
RHOT1	UP	Known DG	apoptosis
RHOT2	UP	Known DG	apoptosis
RORA	UP	TF-Novel	apoptosis
RPS6KA2	UP	variant	apoptosis
RSL1D1	DOWN	Known DG	apoptosis
SFTPA1	DOWN	Novel DG	apoptosis
SIRT1	UP	Known DG	apoptosis
SLC39A10	UP	Known DG	apoptosis
SOD2	UP	Novel DG	apoptosis
SORT1	DOWN	Known DG	apoptosis

SOX2	UP	TF	apoptosis
SOX4	DOWN	TF	apoptosis
SPHK2	DOWN	Known DG	apoptosis
SPRY2	UP	variant	apoptosis
STAT1	DOWN	TF	apoptosis
STAT3	DOWN	TF	apoptosis
STAT5A	DOWN	TF	apoptosis
TAF1	DOWN	TF	apoptosis
TAF6	UP	Novel DG	apoptosis
TCF4	UP	TF	apoptosis
TFDP2	UP	Known DG	apoptosis
TMEM161A	UP	Known DG	apoptosis
TNFAIP3	DOWN	Known DG	apoptosis
TSC2	UP	Novel DG	apoptosis
UNC13B	DOWN	Novel DG	apoptosis
VPS51	UP	Novel DG	apoptosis
VPS53	DOWN	Known DG	apoptosis
WT1	DOWN	TF	apoptosis
ZBTB16	UP	variant	apoptosis
ZBTB7A	UP	TF	apoptosis
ZBTB7C	DOWN	Novel DG	apoptosis
AP2B1	DOWN	Known DG	apoptosis
PICALM	UP	variant	apoptosis
SNAP91	UP	variant	apoptosis
CNN3	UP	variant	ATPase activity
DNAJC19	UP	Novel DG	ATPase activity
DYNLL1	UP	variant	ATPase activity
HSPA8	UP	Novel DG	ATPase activity
LTF	UP	Known DG	ATPase activity
NAPA	DOWN	Known DG	ATPase activity
TMEM64	UP	Novel DG	ATPase activity
TNNT2	UP	Known DG	ATPase activity
APEX1	DOWN	Known DG	Autophagy
ATG2A	DOWN	Novel DG	Autophagy
ATP6V0B	UP	Novel DG	Autophagy
ATP6V1B2	UP	Known DG	Autophagy
BCL2	UP	TF	Autophagy
CAMKK2	UP	Known DG	Autophagy
CEP57	UP	Known DG	Autophagy
DYNLL1	UP	variant	Autophagy
HARS	UP	Novel DG	Autophagy
HDLBP	UP	Known DG	Autophagy
HSPA8	UP	Novel DG	Autophagy
LEPR	DOWN	Novel DG	Autophagy
MLST8	DOWN	Novel DG	Autophagy
MTCH1	UP	variant	Autophagy
MTCL1	DOWN	Novel DG	Autophagy

MTERF3	DOWN	Novel DG	Autophagy
NFYA	DOWN	TF	Autophagy
NPRL2	UP	Novel DG	Autophagy
NR2C2	DOWN	TF	Autophagy
OPTN	UP	Known DG	Autophagy
PIK3R2	UP	Novel DG	Autophagy
PRKACA	UP	Known DG	Autophagy
PSEN1	UP	Known DG	Autophagy
PTK2	UP	Known DG	Autophagy
RAB33B	UP	variant	Autophagy
RNASEH2C	DOWN	Novel DG	Autophagy
SFTPA1	DOWN	Novel DG	Autophagy
SIRT1	UP	Known DG	Autophagy
SREBF2	UP	variant	Autophagy
TSC2	UP	Novel DG	Autophagy
ULK3	DOWN	Novel DG	Autophagy
USP33	DOWN	Known DG	Autophagy
VPS41	DOWN	Known DG	Autophagy
VPS51	UP	Novel DG	Autophagy
APP	UP	variant	axonogenesis
ARHGAP4	DOWN	Known DG	axonogenesis
BAIAP2	UP	variant	axonogenesis
BCL11B	UP	variant	axonogenesis
BCL2	UP	TF	axonogenesis
CD46	UP	Known DG	axonogenesis
DCX	UP	Known DG	axonogenesis
FGF13	DOWN	Known DG	axonogenesis
FLOT1	DOWN	Novel DG	axonogenesis
GATA3	DOWN	TF	axonogenesis
GBX2	DOWN	TF	axonogenesis
GRB10	DOWN	Known DG	axonogenesis
IER3	UP	Novel DG	axonogenesis
ILK	DOWN	variant	axonogenesis
LAMB2	DOWN	Novel DG	axonogenesis
MAG	UP	variant	axonogenesis
NELL2	DOWN	Known DG	axonogenesis
NPTX1	UP	variant	axonogenesis
NRXN1	UP	variant	axonogenesis
NTRK2	UP	variant	axonogenesis
OPTN	UP	Known DG	axonogenesis
PAX6	UP	TF	axonogenesis
PICALM	UP	variant	axonogenesis
PLOD2	DOWN	Novel DG	axonogenesis
PRKACA	UP	Known DG	axonogenesis
PSEN1	UP	Known DG	axonogenesis
PTK2	UP	Known DG	axonogenesis
RGMA	DOWN	Novel DG	axonogenesis

S100A6	DOWN	Known DG	axonogenesis
SEMA4A	DOWN	Novel DG	axonogenesis
SNAP91	UP	variant	axonogenesis
SRF	DOWN	TF	axonogenesis
TNN	UP	Novel DG	axonogenesis
TUBB4A	DOWN	Known DG	axonogenesis
USP33	DOWN	Known DG	axonogenesis
ACE	DOWN	variant	blood pressure/circulation
AKAP9	UP	Novel DG	blood pressure/circulation
ATF7IP	UP	Known DG	blood pressure/circulation
CACNA1C	UP	Novel DG	blood pressure/circulation
CACNA2D1	DOWN	Novel DG	blood pressure/circulation
CELF2	UP	variant	blood pressure/circulation
DCP1B	UP	Novel DG	blood pressure/circulation
DMD	UP	Novel	blood pressure/circulation
FGF13	DOWN	Known DG	blood pressure/circulation
GJC1	UP	Known DG	blood pressure/circulation
KCND3	UP	variant	blood pressure/circulation
MDM2	DOWN	Known DG	blood pressure/circulation
MEF2A	UP	TF	blood pressure/circulation
NPRL2	UP	Novel DG	blood pressure/circulation
NR3C1	UP	TF	blood pressure/circulation
PICALM	UP	variant	blood pressure/circulation
PRKACA	UP	Known DG	blood pressure/circulation
RYR3	DOWN	Known DG	blood pressure/circulation
SLC1A1	UP	Known DG	blood pressure/circulation
SNAP91	UP	variant	blood pressure/circulation

TAC1	DOWN	Known DG	blood pressure/circulation
TNNT2	UP	Known DG	blood pressure/circulation
ACTR1A	DOWN	variant	blood pressure/circulation
CSRP1	UP	Known DG	blood pressure/circulation
NFE2	DOWN	TF	blood pressure/circulation
NTSR1	DOWN	Novel DG	blood pressure/circulation
P2RX2	DOWN	Known DG	blood pressure/circulation
PDE2A	UP	Known DG	blood pressure/circulation
PITX2	DOWN	TF	blood pressure/circulation
POSTN	DOWN	Novel DG	blood pressure/circulation
RPS6KA2	UP	variant	blood pressure/circulation
SCN1A	UP	variant	blood pressure/circulation
SLC2A5	DOWN	Known DG	blood pressure/circulation
SMAD5	DOWN	Known DG	blood pressure/circulation
SOD2	UP	Novel DG	blood pressure/circulation
STAT1	DOWN	TF	blood pressure/circulation
STK39	UP	variant	blood pressure/circulation
ZBTB7C	DOWN	Novel DG	blood pressure/circulation
ACTR1A	DOWN	variant	blood vessel
ADAM8	UP	Known DG	blood vessel
AGO2	DOWN	Novel DG	blood vessel
AP2B1	DOWN	Known DG	blood vessel
ARID2	UP	Novel DG	blood vessel
ATF2	UP	Known DG	blood vessel
ATF7IP	UP	Known DG	blood vessel
BRCA1	DOWN	TF	blood vessel
BRF1	UP	TF	blood vessel
CD46	UP	Known DG	blood vessel
DAB2IP	UP	Novel DG	blood vessel

FGF1	UP	Known DG	blood vessel
FGF13	DOWN	Known DG	blood vessel
GATA2	DOWN	TF	blood vessel
GATA6	DOWN	TF	blood vessel
GBX2	DOWN	TF	blood vessel
GJC1	UP	Known DG	blood vessel
HARS	UP	Novel DG	blood vessel
HDAC9	DOWN	Known DG	blood vessel
HEY1	UP	TF	blood vessel
HOXA1	DOWN	TF	blood vessel
HOXA5	DOWN	TF	blood vessel
JMJD6	UP	TF	blood vessel
JUN	DOWN	TF	blood vessel
KLF5	DOWN	TF	blood vessel
LAMA5	DOWN	variant	blood vessel
LEPR	DOWN	Novel DG	blood vessel
LUZP1	UP	variant	blood vessel
MAGI2	UP	variant	blood vessel
MAP3K3	DOWN	Novel DG	blood vessel
MDM2	DOWN	Known DG	blood vessel
MEN1	UP	Novel DG	blood vessel
MFGE8	DOWN	Known DG	blood vessel
NELL2	DOWN	Known DG	blood vessel
NF1	DOWN	TF	blood vessel
NR3C1	UP	TF	blood vessel
NRXN1	UP	variant	blood vessel
NTRK2	UP	variant	blood vessel
OPTN	UP	Known DG	blood vessel
PAX6	UP	TF	blood vessel
PDCD6IP	UP	Known DG	blood vessel
PITX2	DOWN	TF	blood vessel
PLCD3	UP	Novel DG	blood vessel
PRKACA	UP	Known DG	blood vessel
PSEN1	UP	Known DG	blood vessel
PTK2	UP	Known DG	blood vessel
PTK7	UP	Known DG	blood vessel
RORA	UP	TF-Novel	blood vessel
SAT1	UP	Known DG	blood vessel
SEMA4A	DOWN	Novel DG	blood vessel
SIRT1	UP	Known DG	blood vessel
SOX18	DOWN	TF	blood vessel
SOX4	DOWN	TF	blood vessel
SP1	DOWN	TF	blood vessel
SPHK2	DOWN	Known DG	blood vessel
SPRY2	UP	variant	blood vessel
SRF	DOWN	TF	blood vessel
SRSF11	UP	Known DG	blood vessel

STAT1	DOWN	TF	blood vessel
STAT3	DOWN	TF	blood vessel
TAL1	DOWN	TF	blood vessel
TCF4	UP	TF	blood vessel
TIPARP	UP	Novel DG	blood vessel
TNFAIP3	DOWN	Known DG	blood vessel
VPS51	UP	Novel DG	blood vessel
WARS	DOWN	Known DG	blood vessel
WT1	DOWN	TF	blood vessel
DNMT1	UP	TF	blood vessel
SOD2	UP	Novel DG	blood vessel
A2M	UP	Novel DG	Defense response
ABCF1	UP	Novel DG	Defense response
ADAM8	UP	Known DG	Defense response
ALPK1	DOWN	Known DG	Defense response
APP	UP	variant	Defense response
BCL2	UP	TF	Defense response
BCL3	DOWN	TF	Defense response
BFAR	DOWN	Known DG	Defense response
BTK	DOWN	Known DG	Defense response
C19orf66	DOWN	Known DG	Defense response
C4B	DOWN	Novel DG	Defense response
CADM1	DOWN	Known DG	Defense response
CARD9	DOWN	Novel DG	Defense response
CASP4	UP	Known DG	Defense response
CCAR2	UP	Novel DG	Defense response
CCR9	DOWN	Novel DG	Defense response
CD46	UP	Known DG	Defense response
CEBPG	DOWN	TF	Defense response
CREBBP	UP	TF	Defense response
CSRP1	UP	Known DG	Defense response
CYLD	DOWN	Known DG	Defense response
DAB2IP	UP	Novel DG	Defense response
DDX3X	DOWN	Known DG	Defense response
ESR1	DOWN	TF	Defense response
EXOSC5	UP	Known DG	Defense response
FECH	DOWN	Novel DG	Defense response
FERMT2	UP	Known DG	Defense response
FLOT1	DOWN	Novel DG	Defense response
FPR3	UP	Novel DG	Defense response
FUZ	DOWN	Novel DG	Defense response
GATA3	DOWN	TF	Defense response
GRIK2	UP	variant	Defense response
GSTP1	UP	Known DG	Defense response
HDAC9	DOWN	Known DG	Defense response
HLA-DQA1	UP	Novel DG	Defense response
HSF1	DOWN	TF	Defense response

IFNAR2	DOWN	Novel DG	Defense response
IL17RC	UP	Known DG	Defense response
IP6K2	DOWN	Novel DG	Defense response
IRF1	DOWN	TF	Defense response
IRF3	DOWN	TF	Defense response
IRF4	DOWN	TF	Defense response
IRF5	DOWN	TF	Defense response
IRF7	UP	TF-Novel	Defense response
ISCU	UP	Novel DG	Defense response
JUN	DOWN	TF	Defense response
KARS	DOWN	Known DG	Defense response
KIF16B	UP	Known DG	Defense response
LPO	UP	Novel DG	Defense response
LSM14A	UP	Novel DG	Defense response
LTF	UP	Known DG	Defense response
MAGI2	UP	variant	Defense response
MAPKAPK3	DOWN	Known DG	Defense response
MBP	UP	variant	Defense response
MEN1	UP	Novel DG	Defense response
MTCH1	UP	variant	Defense response
MUM1	UP	Known DG	Defense response
MYC	DOWN	TF	Defense response
NFKB1	DOWN	TF	Defense response
NPAS1	DOWN	Known DG	Defense response
NR1H2	UP	Novel DG	Defense response
NR2C2	DOWN	TF	Defense response
NRF1	UP	TF	Defense response
OPRM1	UP	Known DG	Defense response
OPTN	UP	Known DG	Defense response
OR2H2	DOWN	Novel DG	Defense response
PAN2	DOWN	Novel DG	Defense response
PDCD6IP	UP	Known DG	Defense response
PDE2A	UP	Known DG	Defense response
PITX2	DOWN	TF	Defense response
PQBP1	UP	Known DG	Defense response
PRKACA	UP	Known DG	Defense response
PRKCE	DOWN	variant	Defense response
PSEN1	UP	Known DG	Defense response
PTGES	DOWN	Known DG	Defense response
QARS	UP	Known DG	Defense response
RAB27A	DOWN	Known DG	Defense response
RBBP8	UP	Known DG	Defense response
RIOK3	UP	Known DG	Defense response
RORA	UP	TF-Novel	Defense response
RPL30	DOWN	Known DG	Defense response
SEC14L2	UP	Novel DG	Defense response
SEH1L	UP	Known DG	Defense response

SFTPA1	DOWN	Novel DG	Defense response
SHMT2	DOWN	Known DG	Defense response
STAT1	DOWN	TF	Defense response
STAT2	DOWN	TF	Defense response
STAT3	DOWN	TF	Defense response
STK39	UP	variant	Defense response
STXBP2	UP	Known DG	Defense response
TAC1	DOWN	Known DG	Defense response
TMPO	UP	Known DG	Defense response
TNFAIP3	DOWN	Known DG	Defense response
TNIP1	DOWN	Known DG	Defense response
TRIM28	DOWN	TF	Defense response
TUBB	DOWN	Known DG	Defense response
UNC13B	DOWN	Novel DG	Defense response
ZBTB1	DOWN	TF	Defense response
ZBTB7C	DOWN	Novel DG	Defense response
ZYX	DOWN	variant	Defense response
ATF2	UP	Known DG	Dentin
ATF7IP	UP	Known DG	Dentin
BCL11B	UP	variant	Dentin
FAM20A	UP	Novel DG	Dentin
FAM20C	UP	Novel DG	Dentin
HDAC1	DOWN	TF	Dentin
HSF1	DOWN	TF	Dentin
JAG2	UP	Novel DG	Dentin
LAMA5	DOWN	variant	Dentin
POSTN	DOWN	Novel DG	Dentin
ROGDI	DOWN	Known DG	Dentin
ACTR1A	DOWN	variant	Dentin
ATF2	UP	Known DG	Dentin
ATF7IP	UP	Known DG	Dentin
BCL11B	UP	variant	Dentin
FAM20A	UP	Novel DG	Dentin
FAM20C	UP	Novel DG	Dentin
HDAC1	DOWN	TF	Dentin
HSF1	DOWN	TF	Dentin
JAG2	UP	Novel DG	Dentin
LAMA5	DOWN	variant	Dentin
PITX2	DOWN	TF	Dentin
POSTN	DOWN	Novel DG	Dentin
ROGDI	DOWN	Known DG	Dentin
FOXA2	DOWN	TF	energy metabolism
KAT2B	UP	variant	energy metabolism
LEPR	DOWN	Novel DG	energy metabolism
MYC	DOWN	TF	energy metabolism
NCOR1	DOWN	TF	energy metabolism
NFKB1	DOWN	TF	energy metabolism

NTSR1	DOWN	Novel DG	energy metabolism
PRKCE	DOWN	variant	energy metabolism
RORA	UP	TF-Novel	energy metabolism
SIRT1	UP	Known DG	energy metabolism
STAT3	DOWN	TF	energy metabolism
STK39	UP	variant	energy metabolism
ZBTB7A	UP	TF	energy metabolism
ZBTB7C	DOWN	Novel DG	energy metabolism
BFAR	DOWN	Known DG	energy metabolism
CRY1	UP	Known DG	energy metabolism
DYNLL1	UP	variant	energy metabolism
GRIK2	UP	variant	energy metabolism
HMGN3	UP	TF	energy metabolism
HNF4A	DOWN	TF	energy metabolism
MEN1	UP	Novel DG	energy metabolism
PAX6	UP	TF	energy metabolism
PIK3R2	UP	Novel DG	energy metabolism
PRKACA	UP	Known DG	energy metabolism
SOX4	DOWN	TF	energy metabolism
SRF	DOWN	TF	energy metabolism
TCF4	UP	TF	energy metabolism
UNC13B	DOWN	Novel DG	energy metabolism
VSNL1	UP	variant	energy metabolism
SLC16A11	UP	Novel DG	energy metabolism
SLC16A7	DOWN	Known DG	energy metabolism
BRF1	UP	TF	energy metabolism
RPS6KA2	UP	variant	energy metabolism
SLC2A5	DOWN	Known DG	energy metabolism
APEX1	DOWN	Known DG	exocytosis
DAB2IP	UP	Novel DG	exocytosis
DGKI	UP	Known DG	exocytosis
GATA2	DOWN	TF	exocytosis
GRIK2	UP	variant	exocytosis
HARS	UP	Novel DG	exocytosis
MAGI2	UP	variant	exocytosis
NAPA	DOWN	Known DG	exocytosis
NFYA	DOWN	TF	exocytosis
PDCD6IP	UP	Known DG	exocytosis
PICALM	UP	variant	exocytosis
RAB27A	DOWN	Known DG	exocytosis
RAB33B	UP	variant	exocytosis
REST	DOWN	TF	exocytosis
SNAP91	UP	variant	exocytosis
STXBP2	UP	Known DG	exocytosis
STXBP6	UP	Novel DG	exocytosis
UNC13B	DOWN	Novel DG	exocytosis
VSNL1	UP	variant	exocytosis

APP	UP	variant	gliogenesis
DMD	UP	Novel	gliogenesis
DRG1	DOWN	Known DG	gliogenesis
FPR3	UP	Novel DG	gliogenesis
GSTP1	UP	Known DG	gliogenesis
ILK	DOWN	variant	gliogenesis
LAMB2	DOWN	Novel DG	gliogenesis
MAG	UP	variant	gliogenesis
NF1	DOWN	TF	gliogenesis
NTRK2	UP	variant	gliogenesis
PSEN1	UP	Known DG	gliogenesis
RORA	UP	TF-Novel	gliogenesis
SH3TC2	DOWN	Known DG	gliogenesis
SOX4	DOWN	TF	gliogenesis
TRIB1	DOWN	variant	gliogenesis
CD46	UP	Known DG	gliogenesis
CDKN2C	UP	Known DG	gliogenesis
HDAC1	DOWN	TF	gliogenesis
PAX6	UP	TF	gliogenesis
POLD1	DOWN	Known DG	gliogenesis
SOX2	UP	TF	gliogenesis
STAT3	DOWN	TF	gliogenesis
TAL1	DOWN	TF	gliogenesis
DAB2IP	UP	Novel DG	gliogenesis
DCX	UP	Known DG	gliogenesis
E2F1	DOWN	TF	gliogenesis
EZH2	DOWN	TF	gliogenesis
MAGI2	UP	variant	gliogenesis
MYC	DOWN	TF	gliogenesis
PDCD6IP	UP	Known DG	gliogenesis
GRIA1	UP	variant	glutamate signaling
GRIK2	UP	variant	glutamate signaling
GRM2	UP	Known DG	glutamate signaling
GRM7	UP	Known DG	glutamate signaling
CANX	DOWN	Known DG	glutamate signaling
DLG2	UP	variant	glutamate signaling
FLOT1	DOWN	Novel DG	glutamate signaling
HSPA8	UP	Novel DG	glutamate signaling
SHANK2	DOWN	variant	glutamate signaling
APP	UP	variant	glutamate signaling
DAGLA	DOWN	Known DG	glutamate signaling
NRXN1	UP	variant	glutamate signaling
OPRM1	UP	Known DG	glutamate signaling
ATF2	UP	Known DG	Histone acetylation
BRCA1	DOWN	TF	Histone acetylation
CREBBP	UP	TF	Histone acetylation
CTBP1	UP	TF	Histone acetylation

DPF1	DOWN	Known DG	Histone acetylation
GATA2	DOWN	TF	Histone acetylation
GATA3	DOWN	TF	Histone acetylation
IRF4	DOWN	TF	Histone acetylation
KANSL3	DOWN	Novel DG	Histone acetylation
KAT2B	UP	variant	Histone acetylation
MSL3	DOWN	Novel DG	Histone acetylation
MUM1	UP	Known DG	Histone acetylation
NR2C2	DOWN	TF	Histone acetylation
SIRT1	UP	Known DG	Histone acetylation
TAF1	DOWN	TF	Histone acetylation
MEF2A	UP	TF	Histone acetylation
MPP1	UP	Novel DG	Histone acetylation
PAX6	UP	TF	Histone acetylation
SP1	DOWN	TF	Histone acetylation
STAT1	DOWN	TF	Histone acetylation
ZBTB7A	UP	TF	Histone acetylation
ABCA2	DOWN	Known DG	Homeostasis
ACACA	DOWN	Novel DG	Homeostasis
ACADVL	UP	Known DG	Homeostasis
ADAM8	UP	Known DG	Homeostasis
AIPL1	DOWN	Novel DG	Homeostasis
ALDOA	UP	Novel DG	Homeostasis
ALOXE3	UP	Known DG	Homeostasis
APEX1	DOWN	Known DG	Homeostasis
APP	UP	variant	Homeostasis
ARID2	UP	Novel DG	Homeostasis
ATF2	UP	Known DG	Homeostasis
ATF7IP	UP	Known DG	Homeostasis
ATP6V1B2	UP	Known DG	Homeostasis
BBS1	DOWN	Known DG	Homeostasis
BCL2	UP	TF	Homeostasis
BFAR	DOWN	Known DG	Homeostasis
BRF1	UP	TF	Homeostasis
CACNA1C	UP	Novel DG	Homeostasis
CACNA2D1	DOWN	Novel DG	Homeostasis
CCR9	DOWN	Novel DG	Homeostasis
CCT8	UP	Known DG	Homeostasis
CEBPG	DOWN	TF	Homeostasis
CHKB	DOWN	Novel DG	Homeostasis
CREBBP	UP	TF	Homeostasis
CRY1	UP	Known DG	Homeostasis
CYLD	DOWN	Known DG	Homeostasis
DEF8	DOWN	Known DG	Homeostasis
DMD	UP	Novel	Homeostasis
DYNLL1	UP	variant	Homeostasis
ERCC1	UP	Known DG	Homeostasis

ESR1	DOWN	TF	Homeostasis
ESRRG	UP	Known DG	Homeostasis
EZH2	DOWN	TF	Homeostasis
FAM20A	UP	Novel DG	Homeostasis
FGF13	DOWN	Known DG	Homeostasis
FOXA2	DOWN	TF	Homeostasis
FPR3	UP	Novel DG	Homeostasis
GATA2	DOWN	TF	Homeostasis
GATA3	DOWN	TF	Homeostasis
GNL3	DOWN	Known DG	Homeostasis
GRB10	DOWN	Known DG	Homeostasis
GRIA1	UP	variant	Homeostasis
GRIK2	UP	variant	Homeostasis
GRM2	UP	Known DG	Homeostasis
GSTP1	UP	Known DG	Homeostasis
HMGN3	UP	TF	Homeostasis
HNF4A	DOWN	TF	Homeostasis
HOXA5	DOWN	TF	Homeostasis
HSF1	DOWN	TF	Homeostasis
ILDR2	DOWN	Novel DG	Homeostasis
IRF4	DOWN	TF	Homeostasis
ISCU	UP	Novel DG	Homeostasis
JMJD6	UP	TF	Homeostasis
KCNMA1	UP	variant	Homeostasis
LAT2	UP	Novel DG	Homeostasis
LEPR	DOWN	Novel DG	Homeostasis
LTF	UP	Known DG	Homeostasis
MCUB	DOWN	variant	Homeostasis
MDM1	UP	Known DG	Homeostasis
MEN1	UP	Novel DG	Homeostasis
MPV17	UP	Novel DG	Homeostasis
MTCH1	UP	variant	Homeostasis
MUM1	UP	Known DG	Homeostasis
MYC	DOWN	TF	Homeostasis
NAPA	DOWN	Known DG	Homeostasis
NBL1	DOWN	Known DG	Homeostasis
NCAPG2	UP	Known DG	Homeostasis
NELL2	DOWN	Known DG	Homeostasis
NF1	DOWN	TF	Homeostasis
NFYA	DOWN	TF	Homeostasis
NR1H2	UP	Novel DG	Homeostasis
NRF1	UP	TF	Homeostasis
NTSR1	DOWN	Novel DG	Homeostasis
P2RX2	DOWN	Known DG	Homeostasis
PAX6	UP	TF	Homeostasis
PICALM	UP	variant	Homeostasis
PIK3R2	UP	Novel DG	Homeostasis

POLD1	DOWN	Known DG	Homeostasis
PPP2R3C	UP	Known DG	Homeostasis
PRKACA	UP	Known DG	Homeostasis
PRKCE	DOWN	variant	Homeostasis
PSEN1	UP	Known DG	Homeostasis
RALY	UP	Known DG	Homeostasis
RC3H1	UP	Novel DG	Homeostasis
RHOT1	UP	Known DG	Homeostasis
RHOT2	UP	Known DG	Homeostasis
RORA	UP	TF-Novel	Homeostasis
RYR3	DOWN	Known DG	Homeostasis
SFTPA1	DOWN	Novel DG	Homeostasis
SFXN3	DOWN	Known DG	Homeostasis
SFXN5	DOWN	Known DG	Homeostasis
SIRT1	UP	Known DG	Homeostasis
SLC39A10	UP	Known DG	Homeostasis
SLC4A3	DOWN	Known DG	Homeostasis
SMAD5	DOWN	Known DG	Homeostasis
SNAP91	UP	variant	Homeostasis
SOD2	UP	Novel DG	Homeostasis
SOX4	DOWN	TF	Homeostasis
SREBF2	UP	variant	Homeostasis
SRF	DOWN	TF	Homeostasis
STAT1	DOWN	TF	Homeostasis
STAT3	DOWN	TF	Homeostasis
STAT5A	DOWN	TF	Homeostasis
STAT6	DOWN	TF	Homeostasis
STK39	UP	variant	Homeostasis
STON1	UP	Novel DG	Homeostasis
TAC1	DOWN	Known DG	Homeostasis
TAL1	DOWN	TF	Homeostasis
TCF4	UP	TF	Homeostasis
TM9SF4	UP	Known DG	Homeostasis
TMEM64	UP	Novel DG	Homeostasis
TNFAIP3	DOWN	Known DG	Homeostasis
TNIP1	DOWN	Known DG	Homeostasis
TTC7A	UP	Novel DG	Homeostasis
UNC13B	DOWN	Novel DG	Homeostasis
USF2	UP	TF	Homeostasis
USH1C	DOWN	Known DG	Homeostasis
VSNL1	UP	variant	Homeostasis
XRCC1	DOWN	Known DG	Homeostasis
ZBTB7A	UP	TF	Homeostasis
ZBTB7C	DOWN	Novel DG	Homeostasis
ZFX	DOWN	TF	Homeostasis
A2M	UP	Novel DG	immune response
ALPK1	DOWN	Known DG	immune response

ARPC1B	UP	Novel DG	immune response
BAG6	UP	Novel DG	immune response
BAIAP2	UP	variant	immune response
BCL2	UP	TF	immune response
BFAR	DOWN	Known DG	immune response
BTK	DOWN	Known DG	immune response
C4B	DOWN	Novel DG	immune response
CARD9	DOWN	Novel DG	immune response
CD46	UP	Known DG	immune response
CREBBP	UP	TF	immune response
CSRP1	UP	Known DG	immune response
CYLD	DOWN	Known DG	immune response
DAB2IP	UP	Novel DG	immune response
ELF1	DOWN	TF	immune response
ELMO1	UP	Known DG	immune response
ESR1	DOWN	TF	immune response
FERMT2	UP	Known DG	immune response
FLOT1	DOWN	Novel DG	immune response
FPR3	UP	Novel DG	immune response
GATA3	DOWN	TF	immune response
HLA-DQA1	UP	Novel DG	immune response
IRF1	DOWN	TF	immune response
IRF3	DOWN	TF	immune response
IRF4	DOWN	TF	immune response
IRF7	UP	TF-Novel	immune response
ISCU	UP	Novel DG	immune response
LAT2	UP	Novel DG	immune response
LSM14A	UP	Novel DG	immune response
LTF	UP	Known DG	immune response
MAGI2	UP	variant	immune response
MAPKAPK3	DOWN	Known DG	immune response
MRO	UP	Novel DG	immune response
MTCH1	UP	variant	immune response
MUM1	UP	Known DG	immune response
NFKB1	DOWN	TF	immune response
NR2C2	DOWN	TF	immune response
PAX5	DOWN	TF	immune response
PDCD6IP	UP	Known DG	immune response
PHYH	UP	Novel DG	immune response
PIK3R2	UP	Novel DG	immune response
PITX2	DOWN	TF	immune response
PLD2	UP	Novel DG	immune response
PQBP1	UP	Known DG	immune response
PRKACA	UP	Known DG	immune response
PRKCE	DOWN	variant	immune response
PSEN1	UP	Known DG	immune response
PTK2	UP	Known DG	immune response

RC3H1	UP	Novel DG	immune response
RIOK3	UP	Known DG	immune response
SFTPA1	DOWN	Novel DG	immune response
SLC39A10	UP	Known DG	immune response
TESPA1	UP	Novel DG	immune response
TMPO	UP	Known DG	immune response
TNFAIP3	DOWN	Known DG	immune response
TNIP1	DOWN	Known DG	immune response
JUN	DOWN	TF	immune response
PICALM	UP	variant	immune response
SNAP91	UP	variant	immune response
ACE	DOWN	variant	immune response
ACTR1A	DOWN	variant	immune response
ADAM8	UP	Known DG	immune response
APP	UP	variant	immune response
BCL11B	UP	variant	immune response
BCL3	DOWN	TF	immune response
BRF1	UP	TF	immune response
CACNA1C	UP	Novel DG	immune response
CASP9	UP	Novel DG	immune response
CCR9	DOWN	Novel DG	immune response
CDK2	DOWN	TF	immune response
CEBPG	DOWN	TF	immune response
CRIP2	UP	Novel DG	immune response
DCP1B	UP	Novel DG	immune response
ERCC1	UP	Known DG	immune response
ESRRA	DOWN	TF	immune response
FAM20C	UP	Novel DG	immune response
GATA2	DOWN	TF	immune response
H3F3B	DOWN	Novel DG	immune response
HDAC1	DOWN	TF	immune response
HDAC9	DOWN	Known DG	immune response
HOXA5	DOWN	TF	immune response
HOXA9	DOWN	TF	immune response
JAG2	UP	Novel DG	immune response
JMJD6	UP	TF	immune response
KAT2B	UP	variant	immune response
LEPR	DOWN	Novel DG	immune response
LMO2	UP	TF	immune response
LMO4	UP	Known DG	immune response
MPP1	UP	Novel DG	immune response
MYC	DOWN	TF	immune response
NCAPG2	UP	Known DG	immune response
NF1	DOWN	TF	immune response
NFE2	DOWN	TF	immune response
NFKB2	DOWN	TF	immune response
NPAS1	DOWN	Known DG	immune response

NRF1	UP	TF	immune response
PBX1	UP	TF	immune response
PGAP2	DOWN	Novel DG	immune response
POSTN	DOWN	Novel DG	immune response
PPP2R3C	UP	Known DG	immune response
REST	DOWN	TF	immune response
ROGDI	DOWN	Known DG	immune response
RORA	UP	TF-Novel	immune response
SEMA4A	DOWN	Novel DG	immune response
SIRT1	UP	Known DG	immune response
SMAD5	DOWN	Known DG	immune response
SOX4	DOWN	TF	immune response
SRF	DOWN	TF	immune response
STAT1	DOWN	TF	immune response
STAT3	DOWN	TF	immune response
STAT5A	DOWN	TF	immune response
STAT6	DOWN	TF	immune response
TAL1	DOWN	TF	immune response
TCF12	DOWN	TF	immune response
TCF3	UP	Known DG	immune response
TIPARP	UP	Novel DG	immune response
TMEM64	UP	Novel DG	immune response
TRIB1	DOWN	variant	immune response
TTC7A	UP	Novel DG	immune response
ZBTB1	DOWN	TF	immune response
ZBTB16	UP	variant	immune response
ZBTB7A	UP	TF	immune response
ZBTB7C	DOWN	Novel DG	immune response
ZEB1	UP	TF	immune response
C19orf66	DOWN	Known DG	immune response
CADM1	DOWN	Known DG	immune response
CASP4	UP	Known DG	immune response
DDX3X	DOWN	Known DG	immune response
IFNAR2	DOWN	Novel DG	immune response
IP6K2	DOWN	Novel DG	immune response
IRF5	DOWN	TF	immune response
KIF16B	UP	Known DG	immune response
NR1H2	UP	Novel DG	immune response
OPTN	UP	Known DG	immune response
QARS	UP	Known DG	immune response
RAB27A	DOWN	Known DG	immune response
SEC14L2	UP	Novel DG	immune response
SHMT2	DOWN	Known DG	immune response
STAT2	DOWN	TF	immune response
STXBP2	UP	Known DG	immune response
TRIM28	DOWN	TF	immune response
TUBB	DOWN	Known DG	immune response

ZYX	DOWN	variant	immune response
BFAR	DOWN	Known DG	insulin secretion
DYNLL1	UP	variant	insulin secretion
FOXA2	DOWN	TF	insulin secretion
HMGN3	UP	TF	insulin secretion
PRKCE	DOWN	variant	insulin secretion
VSNL1	UP	variant	insulin secretion
CACNA1C	UP	Novel DG	insulin secretion
CAPN10	DOWN	Novel DG	insulin secretion
HNF4A	DOWN	TF	insulin secretion
ICA1	UP	Known DG	insulin secretion
PRKACA	UP	Known DG	insulin secretion
REST	DOWN	TF	insulin secretion
SOX4	DOWN	TF	insulin secretion
TCF4	UP	TF	insulin secretion
APP	UP	variant	learning
ARF4	UP	Known DG	learning
DGKI	UP	Known DG	learning
FGF13	DOWN	Known DG	learning
JUN	DOWN	TF	learning
MPP1	UP	Novel DG	learning
NDRG4	DOWN	Known DG	learning
NF1	DOWN	TF	learning
NRXN1	UP	variant	learning
NTRK2	UP	variant	learning
NTSR1	DOWN	Novel DG	learning
SHANK2	DOWN	variant	learning
SRF	DOWN	TF	learning
TAC1	DOWN	Known DG	learning
ABCA2	DOWN	Known DG	lipid metabolism
ACACA	DOWN	Novel DG	lipid metabolism
ACADVL	UP	Known DG	lipid metabolism
ACSS2	UP	Novel DG	lipid metabolism
ACTR1A	DOWN	variant	lipid metabolism
AGPAT1	UP	Novel DG	lipid metabolism
AGPAT3	UP	Known DG	lipid metabolism
AGPAT4	UP	variant	lipid metabolism
ALAS1	DOWN	Known DG	lipid metabolism
ALG8	UP	Known DG	lipid metabolism
ALOXE3	UP	Known DG	lipid metabolism
APP	UP	variant	lipid metabolism
ATF7IP	UP	Known DG	lipid metabolism
B3GALNT1	UP	Known DG	lipid metabolism
BCL11B	UP	variant	lipid metabolism
BFAR	DOWN	Known DG	lipid metabolism
BRCA1	DOWN	TF	lipid metabolism
CHEK2	UP	Known DG	lipid metabolism

CHKB	DOWN	Novel DG	lipid metabolism
CHRM5	DOWN	Known DG	lipid metabolism
CREBBP	UP	TF	lipid metabolism
CREM	UP	Novel DG	lipid metabolism
CYP2D6	UP	Novel DG	lipid metabolism
CYP2E1	DOWN	Novel DG	lipid metabolism
DAB2IP	UP	Novel DG	lipid metabolism
DAGLA	DOWN	Known DG	lipid metabolism
DGKD	UP	Novel DG	lipid metabolism
DGKI	UP	Known DG	lipid metabolism
E2F1	DOWN	TF	lipid metabolism
EBPL	DOWN	Novel DG	lipid metabolism
ESR1	DOWN	TF	lipid metabolism
FGF1	UP	Known DG	lipid metabolism
FGF13	DOWN	Known DG	lipid metabolism
FPR3	UP	Novel DG	lipid metabolism
GATA6	DOWN	TF	lipid metabolism
GSTP1	UP	Known DG	lipid metabolism
HAO2	UP	Novel DG	lipid metabolism
HDLBP	UP	Known DG	lipid metabolism
HNF4A	DOWN	TF	lipid metabolism
HSF1	DOWN	TF	lipid metabolism
IER3	UP	Novel DG	lipid metabolism
IP6K2	DOWN	Novel DG	lipid metabolism
IVD	UP	Novel DG	lipid metabolism
LEPR	DOWN	Novel DG	lipid metabolism
LONP1	UP	Known DG	lipid metabolism
LPIN2	UP	Novel DG	lipid metabolism
MAGI2	UP	variant	lipid metabolism
MPDU1	UP	Known DG	lipid metabolism
MTCH1	UP	variant	lipid metabolism
NCOR1	DOWN	TF	lipid metabolism
NFKB1	DOWN	TF	lipid metabolism
NFYA	DOWN	TF	lipid metabolism
NFYB	UP	TF	lipid metabolism
NFYC	DOWN	variant	lipid metabolism
NR1H2	UP	Novel DG	lipid metabolism
NRF1	UP	TF	lipid metabolism
OSBPL8	UP	Novel DG	lipid metabolism
PAN2	DOWN	Novel DG	lipid metabolism
PBX1	UP	TF	lipid metabolism
PDCD6IP	UP	Known DG	lipid metabolism
PEX19	DOWN	Novel DG	lipid metabolism
PGAP2	DOWN	Novel DG	lipid metabolism
PHYH	UP	Novel DG	lipid metabolism
PIK3R2	UP	Novel DG	lipid metabolism
PISD	UP	Novel DG	lipid metabolism

PITX2	DOWN	TF	lipid metabolism
PLCD3	UP	Novel DG	lipid metabolism
PLD2	UP	Novel DG	lipid metabolism
PNPLA8	UP	Novel DG	lipid metabolism
PRKCE	DOWN	variant	lipid metabolism
PTGES	DOWN	Known DG	lipid metabolism
PTK2	UP	Known DG	lipid metabolism
RAB27A	DOWN	Known DG	lipid metabolism
REST	DOWN	TF	lipid metabolism
RORA	UP	TF-Novel	lipid metabolism
RXRA	DOWN	TF	lipid metabolism
SEC14L2	UP	Novel DG	lipid metabolism
SFTPA1	DOWN	Novel DG	lipid metabolism
SH3YL1	DOWN	variant	lipid metabolism
SIRT1	UP	Known DG	lipid metabolism
SLC16A11	UP	Novel DG	lipid metabolism
SP1	DOWN	TF	lipid metabolism
SPHK2	DOWN	Known DG	lipid metabolism
SQLE	DOWN	Known DG	lipid metabolism
SREBF2	UP	variant	lipid metabolism
ST3GAL6	DOWN	Novel DG	lipid metabolism
STAT5A	DOWN	TF	lipid metabolism
TIPARP	UP	Novel DG	lipid metabolism
TTC7A	UP	Novel DG	lipid metabolism
ZBTB7C	DOWN	Novel DG	lipid metabolism
ABCA7	DOWN	Known DG	lipid metabolism
ESRRA	DOWN	TF	lipid metabolism
CRY1	UP	Known DG	lipid metabolism
NTSR1	DOWN	Novel DG	lipid metabolism
TAC1	DOWN	Known DG	lipid metabolism
ACE	DOWN	variant	lipid metabolism
DCP1B	UP	Novel DG	lipid metabolism
MSH3	DOWN	variant	lipid metabolism
QARS	UP	Known DG	lipid metabolism
SLCO4A1	UP	Novel DG	lipid metabolism
SYTL1	DOWN	Novel DG	lipid metabolism
VPS51	UP	Novel DG	lipid metabolism
ATF2	UP	Known DG	membrane permeability
BCL2	UP	TF	membrane permeability
DAB2IP	UP	Novel DG	membrane permeability
E2F1	DOWN	TF	membrane permeability
MAGI2	UP	variant	membrane permeability

PDCD6IP	UP	Known DG	membrane permeability
RHOT1	UP	Known DG	membrane permeability
RHOT2	UP	Known DG	membrane permeability
STAT3	DOWN	TF	membrane permeability
TFDP2	UP	Known DG	membrane permeability
AKAP9	UP	Novel DG	membrane potential
CACNA1C	UP	Novel DG	membrane potential
CACNA2D1	DOWN	Novel DG	membrane potential
DMD	UP	Novel	membrane potential
FGF13	DOWN	Known DG	membrane potential
GJC1	UP	Known DG	membrane potential
GRIK2	UP	variant	membrane potential
KCND3	UP	variant	membrane potential
MYH14	UP	Known DG	membrane potential
NTRK2	UP	variant	membrane potential
NTSR1	DOWN	Novel DG	membrane potential
SCN1A	UP	variant	membrane potential
TAC1	DOWN	Known DG	membrane potential
APP	UP	variant	membrane potential
BAIAP2	UP	variant	membrane potential
BCL2	UP	TF	membrane potential
DGKI	UP	Known DG	membrane potential
DLG2	UP	variant	membrane potential
GABRA2	DOWN	Known DG	membrane potential
GRIA1	UP	variant	membrane potential
GRM7	UP	Known DG	membrane potential
HTR3B	UP	Known DG	membrane potential
JUN	DOWN	TF	membrane potential
KCNMA1	UP	variant	membrane potential
MLST8	DOWN	Novel DG	membrane potential
MYC	DOWN	TF	membrane potential
NRXN1	UP	variant	membrane potential
OPRM1	UP	Known DG	membrane potential
P2RX2	DOWN	Known DG	membrane potential
PPP2R3C	UP	Known DG	membrane potential
PSEN1	UP	Known DG	membrane potential
SAT1	UP	Known DG	membrane potential
SEZ6	DOWN	Novel DG	membrane potential
SHANK2	DOWN	variant	membrane potential
UNC13B	DOWN	Novel DG	membrane potential
ACSS2	UP	Novel DG	mitochondria biogenesis/ regulation

ALAS1	DOWN	Known DG	mitochondria biogenesis/ regulation
ATF2	UP	Known DG	mitochondria biogenesis/ regulation
CREBBP	UP	TF	mitochondria biogenesis/ regulation
ESRRA	DOWN	TF	mitochondria biogenesis/ regulation
MINOS1	DOWN	Novel DG	mitochondria biogenesis/ regulation
NCOR1	DOWN	TF	mitochondria biogenesis/ regulation
NRF1	UP	TF	mitochondria biogenesis/ regulation
RXRA	DOWN	TF	mitochondria biogenesis/ regulation
SOD2	UP	Novel DG	mitochondria biogenesis/ regulation
E2F1	DOWN	TF	mitochondria biogenesis/ regulation
JUN	DOWN	TF	mitochondria biogenesis/ regulation
OPTN	UP	Known DG	mitochondria biogenesis/ regulation
RHOT1	UP	Known DG	mitochondria biogenesis/ regulation
RHOT2	UP	Known DG	mitochondria biogenesis/ regulation
SP1	DOWN	TF	mitochondria biogenesis/ regulation
APEX1	DOWN	Known DG	mitochondria biogenesis/ regulation
ATG2A	DOWN	Novel DG	mitochondria biogenesis/ regulation
BCL2	UP	TF	mitochondria biogenesis/ regulation
CAMKK2	UP	Known DG	mitochondria biogenesis/ regulation
CCAR2	UP	Novel DG	mitochondria biogenesis/ regulation
CDK2	DOWN	TF	mitochondria biogenesis/ regulation
DNAJC19	UP	Novel DG	mitochondria biogenesis/ regulation
LEPR	DOWN	Novel DG	mitochondria biogenesis/ regulation

LONP1	UP	Known DG	mitochondria biogenesis/ regulation
MEF2A	UP	TF	mitochondria biogenesis/ regulation
MPV17	UP	Novel DG	mitochondria biogenesis/ regulation
MYC	DOWN	TF	mitochondria biogenesis/ regulation
MYH14	UP	Known DG	mitochondria biogenesis/ regulation
NDUFAF3	DOWN	Novel DG	mitochondria biogenesis/ regulation
NDUFC1	DOWN	variant	mitochondria biogenesis/ regulation
NDUFS2	UP	Known DG	mitochondria biogenesis/ regulation
NFYA	DOWN	TF	mitochondria biogenesis/ regulation
PDE2A	UP	Known DG	mitochondria biogenesis/ regulation
PICALM	UP	variant	mitochondria biogenesis/ regulation
SNAP91	UP	variant	mitochondria biogenesis/ regulation
SREBF2	UP	variant	mitochondria biogenesis/ regulation
STAT2	DOWN	TF	mitochondria biogenesis/ regulation
STAT3	DOWN	TF	mitochondria biogenesis/ regulation
TFDP2	UP	Known DG	mitochondria biogenesis/ regulation
TIMM44	DOWN	variant	mitochondria biogenesis/ regulation
TIMMDC1	UP	Novel DG	mitochondria biogenesis/ regulation
TSC2	UP	Novel DG	mitochondria biogenesis/ regulation
ACTR1A	DOWN	variant	Neurogenesis
APEX1	DOWN	Known DG	Neurogenesis
APP	UP	variant	Neurogenesis
ARF4	UP	Known DG	Neurogenesis
ARFGEF1	DOWN	variant	Neurogenesis
ARHGAP4	DOWN	Known DG	Neurogenesis
ATF2	UP	Known DG	Neurogenesis
ATF7IP	UP	Known DG	Neurogenesis
BAIAP2	UP	variant	Neurogenesis

BCL11A	UP	TF	Neurogenesis
BCL11B	UP	variant	Neurogenesis
BCL2	UP	TF	Neurogenesis
CAPRIN1	DOWN	Known DG	Neurogenesis
CCAR2	UP	Novel DG	Neurogenesis
CD46	UP	Known DG	Neurogenesis
CDK5RAP3	UP	Novel DG	Neurogenesis
CDKN2C	UP	Known DG	Neurogenesis
CTNND2	DOWN	Known DG	Neurogenesis
CUX1	UP	TF-Novel	Neurogenesis
DAB2IP	UP	Novel DG	Neurogenesis
DAGLA	DOWN	Known DG	Neurogenesis
DCX	UP	Known DG	Neurogenesis
DMD	UP	Novel	Neurogenesis
DRG1	DOWN	Known DG	Neurogenesis
DTNA	UP	Known DG	Neurogenesis
DVL3	UP	Novel DG	Neurogenesis
E2F1	DOWN	TF	Neurogenesis
EZH2	DOWN	TF	Neurogenesis
FGF13	DOWN	Known DG	Neurogenesis
FLOT1	DOWN	Novel DG	Neurogenesis
FOXA2	DOWN	TF	Neurogenesis
FPR3	UP	Novel DG	Neurogenesis
GATA2	DOWN	TF	Neurogenesis
GATA3	DOWN	TF	Neurogenesis
GBX2	DOWN	TF	Neurogenesis
GLDN	DOWN	Novel DG	Neurogenesis
GRB10	DOWN	Known DG	Neurogenesis
GSTP1	UP	Known DG	Neurogenesis
HDAC1	DOWN	TF	Neurogenesis
HDAC9	DOWN	Known DG	Neurogenesis
HEY1	UP	TF	Neurogenesis
HSPA8	UP	Novel DG	Neurogenesis
IER3	UP	Novel DG	Neurogenesis
ILK	DOWN	variant	Neurogenesis
JAG2	UP	Novel DG	Neurogenesis
JUN	DOWN	TF	Neurogenesis
LAMB2	DOWN	Novel DG	Neurogenesis
LMO4	UP	Known DG	Neurogenesis
MAG	UP	variant	Neurogenesis
MAGI2	UP	variant	Neurogenesis
MDM2	DOWN	Known DG	Neurogenesis
MEF2A	UP	TF	Neurogenesis
MTCH1	UP	variant	Neurogenesis
MYC	DOWN	TF	Neurogenesis
MYT1L	UP	variant	Neurogenesis
NAPA	DOWN	Known DG	Neurogenesis

NBL1	DOWN	Known DG	Neurogenesis
NDE1	UP	Novel DG	Neurogenesis
NDRG4	DOWN	Known DG	Neurogenesis
NELL2	DOWN	Known DG	Neurogenesis
NF1	DOWN	TF	Neurogenesis
NFYA	DOWN	TF	Neurogenesis
NPTX1	UP	variant	Neurogenesis
NR3C1	UP	TF	Neurogenesis
NRXN1	UP	variant	Neurogenesis
NTM	UP	Known DG	Neurogenesis
NTRK2	UP	variant	Neurogenesis
OPCML	UP	variant	Neurogenesis
OPRM1	UP	Known DG	Neurogenesis
OPTN	UP	Known DG	Neurogenesis
PAQR3	UP	Known DG	Neurogenesis
PAX6	UP	TF	Neurogenesis
PBX1	UP	TF	Neurogenesis
PDCD6IP	UP	Known DG	Neurogenesis
PDZD7	DOWN	Known DG	Neurogenesis
PICALM	UP	variant	Neurogenesis
PISD	UP	Novel DG	Neurogenesis
PITX2	DOWN	TF	Neurogenesis
PLOD2	DOWN	Novel DG	Neurogenesis
POLD1	DOWN	Known DG	Neurogenesis
POSTN	DOWN	Novel DG	Neurogenesis
PQBP1	UP	Known DG	Neurogenesis
PRKACA	UP	Known DG	Neurogenesis
PSEN1	UP	Known DG	Neurogenesis
PTK2	UP	Known DG	Neurogenesis
PTK7	UP	Known DG	Neurogenesis
PTPRD	UP	variant	Neurogenesis
RARB	UP	Novel DG	Neurogenesis
REST	DOWN	TF	Neurogenesis
RGMA	DOWN	Novel DG	Neurogenesis
ROGDI	DOWN	Known DG	Neurogenesis
RORA	UP	TF-Novel	Neurogenesis
S100A6	DOWN	Known DG	Neurogenesis
SATB2	UP	Known DG	Neurogenesis
SDCCAG8	DOWN	Novel DG	Neurogenesis
SEMA4A	DOWN	Novel DG	Neurogenesis
SEZ6	DOWN	Novel DG	Neurogenesis
SFTPA1	DOWN	Novel DG	Neurogenesis
SH3TC2	DOWN	Known DG	Neurogenesis
SH3YL1	DOWN	variant	Neurogenesis
SHANK2	DOWN	variant	Neurogenesis
SMARCA1	UP	Novel	Neurogenesis
SNAP91	UP	variant	Neurogenesis

SOX1	DOWN	TF	Neurogenesis
SOX14	DOWN	TF	Neurogenesis
SOX2	UP	TF	Neurogenesis
SOX4	DOWN	TF	Neurogenesis
SRF	DOWN	TF	Neurogenesis
STAT3	DOWN	TF	Neurogenesis
STRC	UP	Novel DG	Neurogenesis
TAL1	DOWN	TF	Neurogenesis
TCF12	DOWN	TF	Neurogenesis
TCF3	UP	Known DG	Neurogenesis
TCF4	UP	TF	Neurogenesis
TNN	UP	Novel DG	Neurogenesis
TRIB1	DOWN	variant	Neurogenesis
TUBB4A	DOWN	Known DG	Neurogenesis
USH1C	DOWN	Known DG	Neurogenesis
USP33	DOWN	Known DG	Neurogenesis
ZEB1	UP	TF	Neurogenesis
ALPK1	DOWN	Known DG	NFKB signaling
BFAR	DOWN	Known DG	NFKB signaling
CARD9	DOWN	Novel DG	NFKB signaling
DAB2IP	UP	Novel DG	NFKB signaling
ESR1	DOWN	TF	NFKB signaling
GSTP1	UP	Known DG	NFKB signaling
HDAC1	DOWN	TF	NFKB signaling
IRF3	DOWN	TF	NFKB signaling
LTF	UP	Known DG	NFKB signaling
MAGI2	UP	variant	NFKB signaling
MAP3K3	DOWN	Novel DG	NFKB signaling
NR2C2	DOWN	TF	NFKB signaling
OPTN	UP	Known DG	NFKB signaling
PDCD6IP	UP	Known DG	NFKB signaling
PRKCE	DOWN	variant	NFKB signaling
RIOK3	UP	Known DG	NFKB signaling
RORA	UP	TF-Novel	NFKB signaling
SIRT1	UP	Known DG	NFKB signaling
STAT1	DOWN	TF	NFKB signaling
TNFAIP3	DOWN	Known DG	NFKB signaling
TNIP1	DOWN	Known DG	NFKB signaling
ZBTB7C	DOWN	Novel DG	NFKB signaling
BCL3	DOWN	TF	NFKB signaling
BTK	DOWN	Known DG	NFKB signaling
AAK1	UP	variant	NOTCH signaling
CD46	UP	Known DG	NOTCH signaling
CREBBP	UP	TF	NOTCH signaling
GATA2	DOWN	TF	NOTCH signaling
HEY1	UP	TF	NOTCH signaling
JAG2	UP	Novel DG	NOTCH signaling

KAT2B	UP	variant	NOTCH signaling
NR3C1	UP	TF	NOTCH signaling
POSTN	DOWN	Novel DG	NOTCH signaling
STAT3	DOWN	TF	NOTCH signaling
ZBTB7A	UP	TF	NOTCH signaling
ADAM8	UP	Known DG	proteolysis
APP	UP	variant	proteolysis
BAG6	UP	Novel DG	proteolysis
CASP4	UP	Known DG	proteolysis
CASP9	UP	Novel DG	proteolysis
CDK2	DOWN	TF	proteolysis
DAB2IP	UP	Novel DG	proteolysis
DDX3X	DOWN	Known DG	proteolysis
DYNLL1	UP	variant	proteolysis
HSF1	DOWN	TF	proteolysis
KARS	DOWN	Known DG	proteolysis
MAGI2	UP	variant	proteolysis
MBP	UP	variant	proteolysis
MDM2	DOWN	Known DG	proteolysis
MTCH1	UP	variant	proteolysis
MYC	DOWN	TF	proteolysis
PDCD6IP	UP	Known DG	proteolysis
PICALM	UP	variant	proteolysis
PSEN1	UP	Known DG	proteolysis
PSMC2	DOWN	Known DG	proteolysis
PTK2	UP	Known DG	proteolysis
REST	DOWN	TF	proteolysis
RGMA	DOWN	Novel DG	proteolysis
SFTPA1	DOWN	Novel DG	proteolysis
SIRT1	UP	Known DG	proteolysis
SNAP91	UP	variant	proteolysis
STAT3	DOWN	TF	proteolysis
TAF1	DOWN	TF	proteolysis
TNIP1	DOWN	Known DG	proteolysis
TRIB1	DOWN	variant	proteolysis
VPS53	DOWN	Known DG	proteolysis
DDX39B	UP	Novel DG	regulation of vascular smooth muscle cells
DNMT1	UP	TF	regulation of vascular smooth muscle cells
FGF13	DOWN	Known DG	regulation of vascular smooth muscle cells
GSTP1	UP	Known DG	regulation of vascular smooth muscle cells
JUN	DOWN	TF	regulation of vascular smooth muscle cells

MDM2	DOWN	Known DG	regulation of vascular smooth muscle cells
SOD2	UP	Novel DG	regulation of vascular smooth muscle cells
ZBTB7C	DOWN	Novel DG	regulation of vascular smooth muscle cells
ATF7IP	UP	Known DG	regulation of vascular smooth muscle cells
GATA6	DOWN	TF	regulation of vascular smooth muscle cells
NR3C1	UP	TF	regulation of vascular smooth muscle cells
SRF	DOWN	TF	regulation of vascular smooth muscle cells
BAIAP2	UP	variant	vascular endothelial pathway
DAB2IP	UP	Novel DG	vascular endothelial pathway
ELMO1	UP	Known DG	vascular endothelial pathway
GRB10	DOWN	Known DG	vascular endothelial pathway
HARS	UP	Novel DG	vascular endothelial pathway
MAGI2	UP	variant	vascular endothelial pathway
MAPKAPK3	DOWN	Known DG	vascular endothelial pathway
NELL2	DOWN	Known DG	vascular endothelial pathway
OPTN	UP	Known DG	vascular endothelial pathway
PDCD6IP	UP	Known DG	vascular endothelial pathway
PIK3R2	UP	Novel DG	vascular endothelial pathway
PTK2	UP	Known DG	vascular endothelial pathway
HEY1	UP	TF	vascular endothelial pathway
NR3C1	UP	TF	vascular endothelial pathway
CD46	UP	Known DG	vascular endothelial pathway
FGF13	DOWN	Known DG	vascular endothelial pathway

GATA2	DOWN	TF	vascular endothelial pathway
HDAC9	DOWN	Known DG	vascular endothelial pathway
MAP3K3	DOWN	Novel DG	vascular endothelial pathway
NF1	DOWN	TF	vascular endothelial pathway
PRKACA	UP	Known DG	vascular endothelial pathway
SIRT1	UP	Known DG	vascular endothelial pathway
SOX18	DOWN	TF	vascular endothelial pathway
SP1	DOWN	TF	vascular endothelial pathway
SRF	DOWN	TF	vascular endothelial pathway
STAT5A	DOWN	TF	vascular endothelial pathway
VPS51	UP	Novel DG	vascular endothelial pathway
SPRY2	UP	variant	vascular endothelial pathway
TCF4	UP	TF	vascular endothelial pathway
AAK1	UP	variant	vesicle transport
ABCA7	DOWN	Known DG	vesicle transport
AP2B1	DOWN	Known DG	vesicle transport
APEX1	DOWN	Known DG	vesicle transport
C2CD5	DOWN	Known DG	vesicle transport
C4B	DOWN	Novel DG	vesicle transport
DAB2IP	UP	Novel DG	vesicle transport
DGKI	UP	Known DG	vesicle transport
FLOT1	DOWN	Novel DG	vesicle transport
GATA2	DOWN	TF	vesicle transport
GRIK2	UP	variant	vesicle transport
HARS	UP	Novel DG	vesicle transport
LRRN2	UP	Novel	vesicle transport
MAGI2	UP	variant	vesicle transport
MFGE8	DOWN	Known DG	vesicle transport
MTCH1	UP	variant	vesicle transport
NAPA	DOWN	Known DG	vesicle transport
NDRG4	DOWN	Known DG	vesicle transport
NFYA	DOWN	TF	vesicle transport
NR1H2	UP	Novel DG	vesicle transport
OPTN	UP	Known DG	vesicle transport

PDCD6IP	UP	Known DG	vesicle transport
PICALM	UP	variant	vesicle transport
RAB27A	DOWN	Known DG	vesicle transport
RAB33B	UP	variant	vesicle transport
RAB4B	DOWN	Novel DG	vesicle transport
REST	DOWN	TF	vesicle transport
SFTPA1	DOWN	Novel DG	vesicle transport
SNAP91	UP	variant	vesicle transport
STON1	UP	Novel DG	vesicle transport
STXBP2	UP	Known DG	vesicle transport
STXBP6	UP	Novel DG	vesicle transport
TBC1D4	UP	Novel DG	vesicle transport
TSC2	UP	Novel DG	vesicle transport
UNC13B	DOWN	Novel DG	vesicle transport
VPS41	DOWN	Known DG	vesicle transport
VSNL1	UP	variant	vesicle transport
ZBTB7C	DOWN	Novel DG	vesicle transport
AES	DOWN	Novel DG	WNT signaling
APP	UP	variant	WNT signaling
CCAR2	UP	Novel DG	WNT signaling
CTNND2	DOWN	Known DG	WNT signaling
CYLD	DOWN	Known DG	WNT signaling
DAB2IP	UP	Novel DG	WNT signaling
DDX3X	DOWN	Known DG	WNT signaling
DVL3	UP	Novel DG	WNT signaling
ESR1	DOWN	TF	WNT signaling
FUZ	DOWN	Novel DG	WNT signaling
GLI1	UP	TF	WNT signaling
GRB10	DOWN	Known DG	WNT signaling
HDAC1	DOWN	TF	WNT signaling
ILK	DOWN	variant	WNT signaling
MAGI2	UP	variant	WNT signaling
NFKB1	DOWN	TF	WNT signaling
PDCD6IP	UP	Known DG	WNT signaling
PSEN1	UP	Known DG	WNT signaling
PTK7	UP	Known DG	WNT signaling
RORA	UP	TF-Novel	WNT signaling
SOX2	UP	TF	WNT signaling
SOX4	DOWN	TF	WNT signaling
TCF3	UP	Known DG	WNT signaling
TCF4	UP	TF	WNT signaling
TLE4	DOWN	Known DG	WNT signaling
TMEM64	UP	Novel DG	WNT signaling
TNFAIP3	DOWN	Known DG	WNT signaling
TNN	UP	Novel DG	WNT signaling
TRABD2A	UP	Known DG	WNT signaling
TSC2	UP	Novel DG	WNT signaling

APP	UP	variant	chemical transmission
BAIAP2	UP	variant	chemical transmission
DGKI	UP	Known DG	chemical transmission
DLG2	UP	variant	chemical transmission
GRIK2	UP	variant	chemical transmission
NRXN1	UP	variant	chemical transmission
NTSR1	DOWN	Novel DG	chemical transmission
OPRM1	UP	Known DG	chemical transmission
P2RX2	DOWN	Known DG	chemical transmission
SEZ6	DOWN	Novel DG	chemical transmission
SHANK2	DOWN	variant	chemical transmission
UNC13B	DOWN	Novel DG	chemical transmission
AKAP9	UP	Novel DG	chemical transmission
AP2B1	DOWN	Known DG	chemical transmission
GABRA2	DOWN	Known DG	chemical transmission
GNB2	DOWN	Novel DG	chemical transmission
GNG4	UP	Novel DG	chemical transmission
GNGT2	DOWN	Novel DG	chemical transmission
GRIA1	UP	variant	chemical transmission
HTR3B	UP	Known DG	chemical transmission
KCNJ6	UP	variant	chemical transmission
MDM2	DOWN	Known DG	chemical transmission
PRKACA	UP	Known DG	chemical transmission
RPS6KA2	UP	variant	chemical transmission
TSPAN7	UP	Novel	chemical transmission
GAD1	UP	Known DG	chemical transmission
HSPA8	UP	Novel DG	chemical transmission
PPFIA4	UP	variant	chemical transmission
SLC1A1	UP	Known DG	chemical transmission
BTK	DOWN	Known DG	chemical transmission
CAPS	DOWN	Novel DG	chemical transmission
FLOT1	DOWN	Novel DG	chemical transmission
ICA1	UP	Known DG	chemical transmission
NAPA	DOWN	Known DG	chemical transmission
NF1	DOWN	TF	chemical transmission
PICALM	UP	variant	chemical transmission
PSEN1	UP	Known DG	chemical transmission
RHOT1	UP	Known DG	chemical transmission
SAT1	UP	Known DG	chemical transmission
SLC6A8	UP	Known DG	chemical transmission
SNAP91	UP	variant	chemical transmission
APEX1	DOWN	Known DG	chemical transmission
CD46	UP	Known DG	chemical transmission
CHRM5	DOWN	Known DG	chemical transmission
CNTNAP4	UP	Novel DG	chemical transmission
DTNA	UP	Known DG	chemical transmission
GJC1	UP	Known DG	chemical transmission

GRM2	UP	Known DG	chemical transmission
GRM7	UP	Known DG	chemical transmission
LRRN2	UP	Novel	chemical transmission
MBP	UP	variant	chemical transmission
NFYA	DOWN	TF	chemical transmission
NPTX1	UP	variant	chemical transmission
NTRK2	UP	variant	chemical transmission
PRKCE	DOWN	variant	chemical transmission
RORA	UP	TF-Novel	chemical transmission
SRF	DOWN	TF	chemical transmission
STAT3	DOWN	TF	chemical transmission
SYNPR	DOWN	Novel DG	chemical transmission
TAC1	DOWN	Known DG	chemical transmission
ZBTB7C	DOWN	Novel DG	chemical transmission
CDK2	DOWN	TF	ROS response
STAT6	DOWN	TF	ROS response
CDKN2C	UP	Known DG	ROS response
E2F1	DOWN	TF	ROS response
E2F3	DOWN	TF	ROS response
EZH2	DOWN	TF	ROS response
H3F3B	DOWN	Novel DG	ROS response
JUN	DOWN	TF	ROS response
MAPKAPK3	DOWN	Known DG	ROS response
MDM2	DOWN	Known DG	ROS response
SCMH1	DOWN	Novel DG	ROS response
TFDP2	UP	Known DG	ROS response
APEX1	DOWN	Known DG	ROS response
APP	UP	variant	ROS response
ATF2	UP	Known DG	ROS response
BCL2	UP	TF	ROS response
BTK	DOWN	Known DG	ROS response
CAMKK2	UP	Known DG	ROS response
CYP2E1	DOWN	Novel DG	ROS response
EIF2A	UP	Novel DG	ROS response
ERCC1	UP	Known DG	ROS response
FECH	DOWN	Novel DG	ROS response
GSTP1	UP	Known DG	ROS response
HSF1	DOWN	TF	ROS response
LONP1	UP	Known DG	ROS response
LPO	UP	Novel DG	ROS response
MPV17	UP	Novel DG	ROS response
MSRA	UP	variant	ROS response
MTCH1	UP	variant	ROS response
NDUFS2	UP	Known DG	ROS response
NRF1	UP	TF	ROS response
POLD1	DOWN	Known DG	ROS response
PRKRA	UP	Novel DG	ROS response

PSEN1	UP	Known DG	ROS response
RAD52	DOWN	Known DG	ROS response
SFTPA1	DOWN	Novel DG	ROS response
SIRT1	UP	Known DG	ROS response
SOD2	UP	Novel DG	ROS response
SP1	DOWN	TF	ROS response
STAT1	DOWN	TF	ROS response
TMEM161A	UP	Known DG	ROS response
TNFAIP3	DOWN	Known DG	ROS response
XRCC1	DOWN	Known DG	ROS response
ZBTB7C	DOWN	Novel DG	ROS response

Supplementary Table S8: Biological function of differentially expressed genes (novel and known genes)

**Biological function**	**Novel DG (Fold change)**	**Known AD-associated DG (Fold change)**
**Vascular endothelial pathway**	PIK3R2 (+2.7) , DAB2IP (+5)	PRKACA (+3), SIRT1 (+3.6), OPTN (+2.3),CD46 (+2.7), PTK2 (+3.4), HDAC9 (-6.2), NR3C1(+2.9)
**Regulation of vascular smooth muscle cell**	SOD2 (+2.9), ZBTB7C(-2.5)	MDM2 (-5.3), SIRT1 (+3.6), GSTP1 (+4.3), NR3C1(+2.9), DNMT1(+2.9)
**Regulation of blood vessel (Angiogenesis)**	RORA(+3.2), SOD2 (+2.9), LEPR (-2.3), MEN1 (+2.8)	PRKACA (+3), SIRT1 (+3.6), OPTN (+2.3), HDAC9 (-6.2), MDM2 (-5.3), PSEN1(+3.2), NR3C1(+2.9), DNMT1(+2.9)
**Blood pressure regulation**	SOD2 (+2.9), POSTN (-3.1), NTSR1 (-2.5), CACNA1C (+4), CACNA2D (-2.2)	PRKACA (+3), MDM2 (-5.3), SIRT1(+3.6), TAC1(-2.2)
**Insulin regulation**	CACNA1C (+4)	PRKACA (+3)
**Energy metabolism**	LEPR (-2.3), RORA(+3.2), PIK3R2 (+2.7) , ZBTB7C(-2.5)	SIRT1(+3.6), PRKACA (+3)
**Gliogenesis**	RORA(+3.2), DAB2IP (+5)	PSEN1(+3.2), GSTP1 (+4.3), CD46 (+2.7)
**Glutamate signaling**	FLOT1 (-4.9)	GRM2 (+3), GRM7 (+3.3)
**ROS response**	SOD2 (+2.9), ZBTB7C(-2.5), SFTPA1 (-2.5)	PSEN1(+3.2), MDM2 (-5.3), SIRT1(+3.6), CAMKK2 (+2.5)
**Cytokine/immune response**	IRF7 (+2.8), RORA(+3.2), POSTN (-3.1), SFTPA1 (-2.5), ZBTB7C(-2.5), DAB2IP (+5)	MUM1(+2), PSEN1(+3.2), HDAC9 (-6.2), PPP2R3C (+2),
**Mitochondria morphology regulation**	LEPR (-2.3), SOD2 (+2.9), CCAR2 (+6), DNAJC19 (+4.2), TSC2(+4.9), MINOS1(-2.7)	OPTN (+2.3), CAMKK2 (+2.5)
**Proteolysis**	DAB2IP (+5), SFTPA1 (-2.5)	PSEN1(+3.2), MDM2 (-5.3), SIRT1 (+3.6), PSMC2(-3.6), ABCA7 (-2.7)
**Defense response**	IRF7 (+2.8), RORA(+3.2), CCAR2 (+6), SFTPA1 (-2.5), FLOT1 (-4.9), HLA-DQA1 (+3)	MDM2 (-5.3), OPTN (+2.3), GSTP1 (+4.3), HDAC9 (-6.2),MUM1(+2)
**Vesicle transport**	ZBTB7C(-2.5), SFTPA1 (-2.5), FLOT1 (-4.9), DAB2IP (+5), TSC2(+4.9)	ABCA7 (-2.7), OPTN (+2.3), VPS41 (-6.6)
**Regulation of membrane potential**	CACNA1C (+4), CACNA2D (-2.2),	PPP2R3C (+2), PSEN1(+3.2), GRM7 (+3.3), TAC1(-2.2)
**Homeostasis**	RORA(+3.2), SOD2 (+2.9), ZBTB7C(-2.5), SFTPA1 (-2.5)	PPP2R3C (+2), PSEN1(+3.2), MDM2 (-5.3), PRKACA (+3)
**Apoptosis**	DIDO1(+6.3), IRF7 (+2.8), RORA(+3.2), TSC2(+4.9), ZBTB7C(-2.5), SFTPA1 (-2.5)	PSEN1(+3.2), SIRT1 (+3.6), OPTN (+2.3), MDM2 (-5.3), PRKACA (+3)

+ Upregulation, **-** Downregulation
